# A Phosphotriester‐Masked Dideoxy‐cGAMP Derivative as a Cell‐Permeable STING Agonist

**DOI:** 10.1002/anie.202416353

**Published:** 2024-11-25

**Authors:** Anna‐Lena J. Halbritter, Yasmin V. Gärtner, Jahongir Nabiev, Fabian Hernichel, Giacomo Ganazzoli, Dilara Özdemir, Aikaterini Pappa, Simon Veth, Samuele Stazzoni, Markus Müller, Veit Hornung, Thomas Carell

**Affiliations:** ^1^ Department of Chemistry Institute for Chemical Epigenetics Ludwig-Maximilians-Universität München Butenandtstr. 5–13 81377 Munich Germany; ^2^ Gene Center and Department of Biochemistry Ludwig-Maximilians-Universität München Feodor-Lynen-Str. 25 81377 Munich Germany

**Keywords:** cyclic dinucleotides, immuno-oncology, prodrugs, STING agonists, thioesters

## Abstract

2′,3′‐Cyclic GMP‐AMP (cGAMP) is a cyclic dinucleotide second messenger in which guanosine and adenosine are connected by one 3′‐5′ and one 2′‐5′ phosphodiester linkage. It is formed in the cytosol upon detection of pathogenic DNA by the enzyme guanosine‐monophosphate‐adenosine monophosphate synthase (cGAS). cGAMP subsequently binds to the adaptor protein *stimulator of interferon genes* (STING) to elicit an innate immune response leading to the production of type I interferons and cytokines. STING agonists are a highly promising avenue for an immuno‐oncological anticancer therapy. A particular challenge with cyclic dinucleotide STING agonists are the two negative charges of the phosphodiester linkages, which strongly reduce the ability of such compounds to penetrate cell membranes. The development of cell‐permeable STING agonists that can stimulate the immune system enhancing their anticancer potency is currently of utmost importance in the field. Herein, we report the development of a dideoxy derivative of cGAMP as a phosphotriester prodrug, where the negative charge of the phosphate backbone has been masked with a thioester. We found that this thioester‐protected compound features a dramatic increase in its cellular potency that rises from EC_50_=5 μM to 25 nM. The new compound is envisioned to enable an efficient STING‐agonist‐based anticancer therapy.

## Graphical abstract

A dideoxy analog of cGAS/STING second messenger 2′,3′‐cyclic GMP‐AMP (cGAMP) was prepared using a new and concise synthetic combination of phosphoramidite and phosphotriester chemistry. By masking the negative charges of the phosphate backbones, the cellular uptake of cGAMP was highly increased from EC_50_=5 μM to 25 nM.

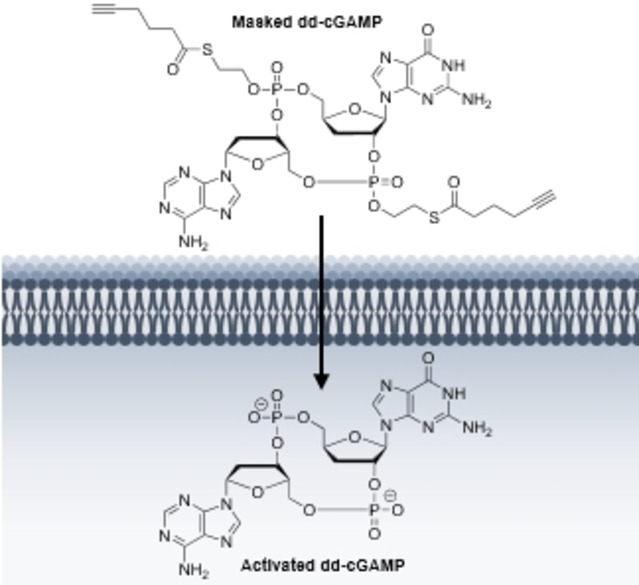

Cyclic dinucleotides, which were initially discovered in bacteria are potent secondary messengers meanwhile identified in both prokaryotic and eukaryotic cells.[^1^, ^2^] Recently it was observed that the detection of pathogenic DNA in the cytosol, either in response to a viral infection or because of the release of nuclear or mitochondrial DNA, leads to the formation of the cyclic dinucleotide 2′,3′‐cyclic guanosine monophosphate‐adenosine monophosphate (cGAMP) (**1**, Figure 1A).^[3]^ The molecule is formed by the enzyme guanosine monophosphate‐adenosine monophosphate synthase (cGAS) upon binding to DNA. cGAS cyclizes one adenosine triphosphate and one guanosine triphosphate to give a cyclic dinucleotide with one 2′‐5′ and one 3′‐5′ phosphodiester linkage. This structure, formed in the cytosol, contains two negative charges. It binds tightly to the endoplasmic reticulum transmembrane protein *stimulator of interferon genes* (STING) to initiate an innate immune response reacting to the challenged state of the cell.[^4^, ^5^, ^6^]

**Figure 1:**
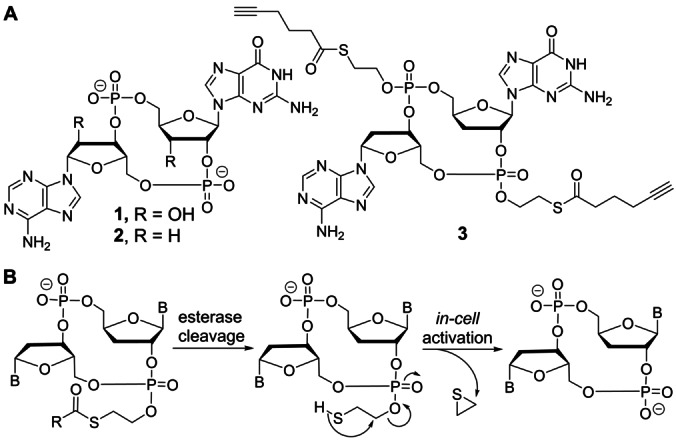
A) cGAMP **1**, dd‐cGAMP **2** and the thioester‐protected cyclic dinucleotide **3** prepared for this study. B) Mechanism of cleavage of the thioester protecting group.

STING agonists that are able to cross cell membranes would offer the possibility to stimulate the immune system from the outside.^[7]^ This in turn would allow to establish a powerful immuno‐oncological treatment, for example as part of an anti‐cancer therapy. Nevertheless, attempts to use cGAMP for this purpose or its derivatives in which the 2′‐ and 3′‐hydroxy groups have been replaced by fluorine atoms or the phosphodiesters by phosphothioates, have thus far not been successful.[^8^, ^9^, ^10^, ^11^, ^12^, ^13^, ^14^]

An alternative approach is to change the two phosphodiesters into triester‐prodrugs. This eliminates the charge impeding cell penetration.^[15]^ If the triester could be cleaved inside the cell to a diester, it would allow the liberation of cGAMP or a close analog to stimulate the STING receptor. A well‐established concept to mask phosphodiesters is the conversion into a thioester‐containing phosphotriester (Figure 1).[^16^, ^17^] Upon cleavage of the thioester, a thioate is generated just four atoms away from the phosphotriester, which leads to the specific cleavage of the desired P−O bond (Figure 1B). This concept has the caveat that the 3′‐ and 2′‐hydroxy groups within cGAMP would quickly attack the phosphotriesters in their close proximity, which leads to an intramolecular cleavage of the triesters and hence instability of the prodrug. To circumvent this problem, the 2′‐ and 3′‐hydroxy groups must be either changed, for example into F‐atoms, or they need to be removed. We decided in a first attempt to investigate the concept of removing these internal nucleophiles, which led to the target compound dideoxy (dd)‐cGAMP (**2**, Figure 1A) **3** (Figure 1A) with both phosphodiesters masked as phosphotriesters. To facilitate potential late‐stage functionalization of the triester‐protecting group, for example, for the attachment of targeting units by click chemistry to enhance the delivery efficiency, we decided to equip the protecting group with an additional terminal alkyne functionality by addition of a 5‐hexynoic thioester (HTE) (Figure 1A) to the phosphodiesters. This gives a new thioester‐protecting group that can be click‐functionalized. We showed previously that click reactions proceed on oligonucleotides with extraordinary efficiency.[^18^, ^19^, ^20^] Synthetically, we first prepared the reference compound dd‐cGAMP **2** that has already been reported by us.^[21]^ The synthesis of the doubly HTE‐protected dd‐cGAMP **3** began with nucleoside **4** (Scheme 1A) which was synthesized according to published procedures (see precursors in the Supporting Information).[^21^, ^22^, ^23^, ^24^] As we wanted to use a thioester to shield the negative charge of the phosphates, we had to come up with a protecting group strategy for the exocyclic amines of guanosine and adenosine that is compatible with the thioesters. We decided to use a photolabile protecting group (PPG), a 4,5‐dimethoxy‐2‐nitrobenzyl derivative (Scheme 1C), for the N2 position of guanosine and N6 position of adenosine. Firstly, this PPG is orthogonal to other protecting groups used for the synthesis of **3**, secondly, it is stable throughout the synthesis of **3** and thirdly it is easily removed by light. We used the commercially available 4,5‐dimethoxy‐2‐nitrobenzyl (Scheme 1C, **5**) as a starting point and prepared first the activated PPG **6**. PPG **6** was then incorporated onto the N2 position of **4** with a crown‐ether under basic conditions to yield the photolabile‐protected 3′‐deoxyguanosine **7**. The TBS groups of **7** were removed, the 5′‐hydroxy was protected as 4,4′‐dimethoxytrityl ether, and the 3′‐hydroxy was phosphitylated to give phosphoramidite **8**. The HTE protecting group (Scheme 1D, **9**) is prepared with 5‐hexynoic acid **10** and 2‐mercaptoethanol **11** and coupled onto phosphoramidite **8** (Scheme 1A) with the 5‐(benzylthio)‐1*H*‐tetrazole (BTT) activator. The phosphoramidite was oxidized and finally the dimethoxytrityl (DMTr) group at the 5′‐position was removed under acidic conditions yielding in phosphotriester **12**. Next, we moved onto the 2′‐deoxyadenosine building block and decided to use the same PPG as we used for the N2 position of guanosine to protect the N6 position of adenosine. The activated PPG **6** was incorporated onto the N6 of TBS protected adenosine **13** (Scheme 1B) in a similar fashion as for the 3′‐deoxyguanosine. The TBS groups were subsequently removed, and the 5′‐hydroxy group was protected as a 4,4′‐dimethoxytrityl ether to give adenosine **14**. The nucleoside was phosphitylated with the HTE‐phosphor reagent **15**, (prepared from **9**), giving HTE‐phosphoramidite **16**. The guanosine building block **12** and adenosine building block **16** were coupled together with BTT at the phospho‐backbone of adenosine and at the 5′‐hydroxy group of guanosine. Oxidation of the phosphoramidite and removal of the DMTr group on the 5′‐hydroxy group yielded in the linearly coupled dinucleotide **17**. The cyanoethyl group was removed, followed by cyclization which resulted in cyclic dinucleotide **18**. Finally, the PPGs were removed to give bis‐HTE‐dd‐cGAMP **3**.

**Scheme 1:**
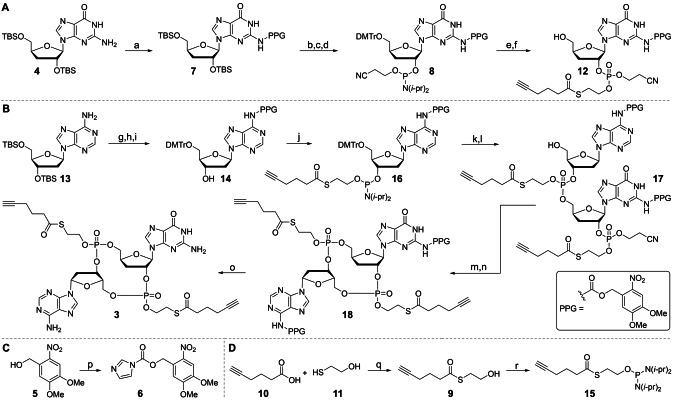
A) Synthesis of 3’‐deoxy guanosine **12**. B) Synthesis of bis‐HTE‐dd‐cGAMP **3**. C) Synthesis of the photolabile protecting group **6**. C) Synthesis of the HTE‐protecting reagents **9** and **15**. Conditions: a) **6**, 18‐crown‐6, NaH, THF, 0 °C—rt, 14 h, 61 % yield; b) TBAF, THF, rt, 14 h, 84 % yield; c) DMTrCl, DMAP, pyridine, rt, 2 d, 77 % yield; d) 2‐Cyanoethyl *N,N,N’,N’*‐tetraisopropylphosphorodiamidite, DIPAT, CH_2_Cl_2_, rt, overnight; e) 1. BTT, CH_3_CN, rt, 2 h. 2. TBHP, rt, 30 min; f) 3 % *v*/*v* DCA, CH_2_Cl_2_, rt, 10 min, 58 % yield; g) **7**, 18‐crown‐6, NaH, THF, 0 °C–rt, 24 h, 85 % yield; h) TBAF, THF, rt, overnight, 62 % yield; i) DMTrCl, DMAP, pyridine rt, 2 d, 82 % yield; j) **16**, DIPAT, CH_2_Cl_2_, rt, overnight; k) 1. **12**, **16**, BTT, CH_3_CN, rt, 2 h. 2. TBHP, rt, 30 min; l) 3 % *v*/*v* DCA, CH_2_Cl_2_, rt, 10 min, 75 % yield; m) *t*BuNH_2_, CH_3_CN rt, 30 min, 71 % yield; n) 2,4,6‐triisopropylbenzenesulfonyl chloride, NMI, THF, rt, 2 d, 58 % yield; q) hν (365 nm), CH_3_CN, rt, 12 min, 63 % yield p) CDI, THF, 0 °C, 1 h, rt, 30 min, 71 % yield; q) DCC, CH_3_CN, 0 °C–rt, overnight, 62 % yield; r) bis(diisopropylamino)chlorophosphine, Et_3_N, Et_2_O, rt, 18 h, 92 % yield. PPG: photolabile protecting group.

We then evaluated whether the newly developed HTE phosphotriester protecting groups on **3** are cleaved by carboxylesterase‐1 (CES1), an enzyme expressed at elevated levels in cells with high metabolic activity such as liver cells, monocytes, macrophages, lung cells and is even overexpressed in certain types of cancer cells such as gallbladder and liver cancer.[^25^, ^26^, ^27^] For the experiment, **3** was treated with the purified CES1 enzyme and the reaction was monitored by HPLC (Figure 2A). To our delight, we could see that the HTE groups were indeed efficiently cleaved (Figure 2A). The enzyme cleaves the thioester, followed by the anticipated cleavage of the correct P−O bond (Figure 1B). Clearly visible is the time‐dependent depletion of bis‐HTE‐dd‐cGAMP **3** (orange dotted‐line box) and simultaneous formation of first the mono‐protected intermediate (black dotted‐line box), where one HTE group has been cleaved, and secondly dd‐cGAMP **2** (blue dotted‐line box) with its two negative charges (Figures 2A and S1). All three detected species found in the HPLC were collected during the measurement and identified by LC–MS analysis (Figure S2). We noted no other reaction product, showing that the cleavage is a clean process without the formation of linearized dinucleotide side‐products. Such compounds would indicate unwanted P−O bond cleavage of the 2′‐ or 3′‐deoxyribose moieties. We next studied the intracellular cleavage of the HTE‐groups. For this experiment, we treated THP1 cells with bis‐HTE‐dd‐cGAMP **3** and harvested them after different time points. We subsequently extracted the cellular metabolites, prepared a small‐molecule enriched fraction and analyzed it by HPLC‐MS (Figure 2B) using the corresponding mass filters. After 30 min (Figure 2B, top) a peak for **3** (Figure 2B, left) and a small peak of the cleaved product **2** (Figure 2B, top) can be detected. After 4 h (Figure 2B, bottom) a significant amount of **3** was already fully converted into the deprotected compound dd‐cGAMP **2**. A small amount of the initial compound, however, could still be detected likely due to ongoing influx into the cells. It should be noted that at this point the intracellularly formed dd‐cGAMP **2** features two negative charges, which hampers its passive escape across the cell membrane. As such dd‐cGAMP **2** is trapped and accumulates inside the cell.

**Figure 2:**
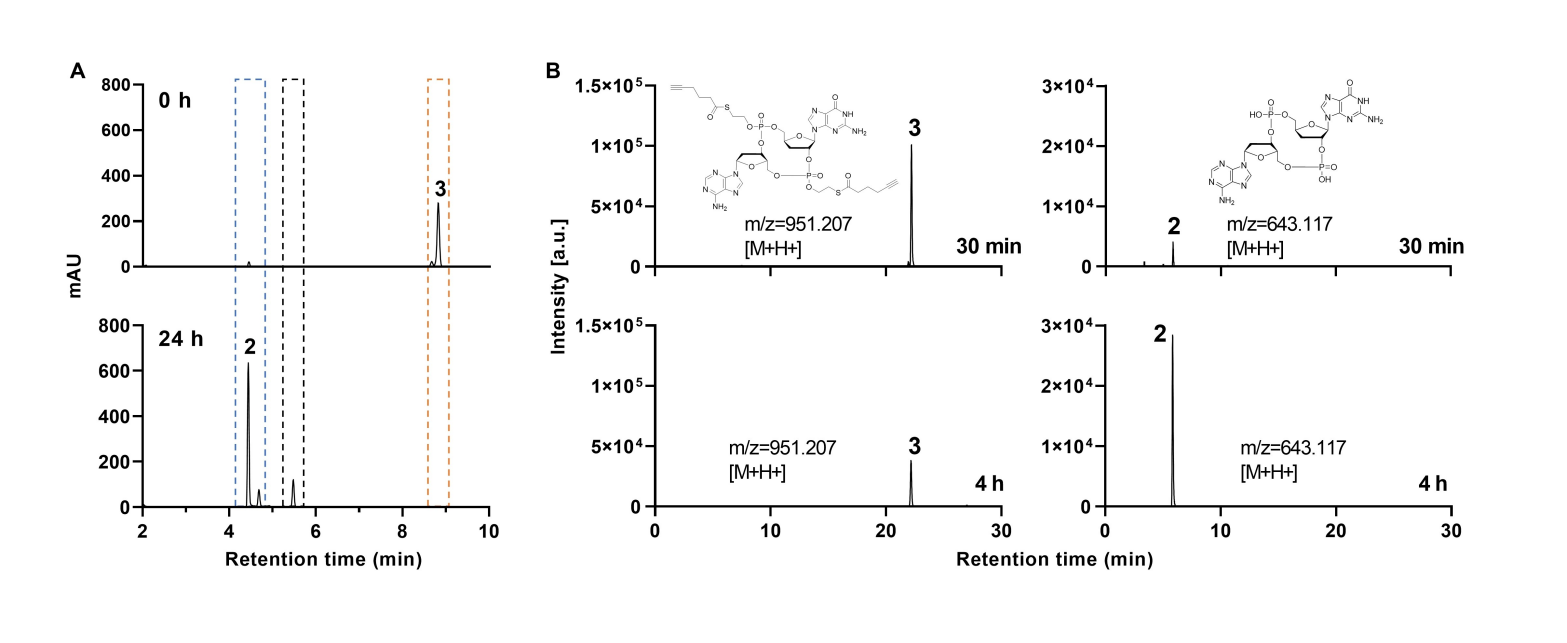
A) HPLC chromatograms of the *in vitro* cleavage of bis‐HTE‐dd‐cGAMP **3** with enzyme CES1 after 0 h (top) and 24 h (bottom). The amount of **3** decreases (orange dotted‐line box) with ongoing incubation time in the presence of CES1. Simultaneously, the amount of unprotected dd‐cGAMP **2** increases (blue dotted‐line box) with incubation time (see Figures S1 for more time‐points). During the cleavage, an intermediate is observed that carries one HTE protecting group (black dotted‐line box). B) Extracted ion chromatograms representing the intracellular cleavage in THP1 cells after 30 min (top) and 4 h (bottom) of **3** (left) to **2** (right). After 30 min, we can see a peak for **3** and a small peak of the cleaved dd‐cGAMP **2**. After 4 h, a small peak is seen for **3** but a larger peak of **2** is observed.

To measure the ability of the compounds to activate STING, we performed a concentration‐dependent study of the interferon (IFN) response. For this purpose, we used the THP1‐Dual^TM^ reporter cell line (InvivoGen). These cells feature a secreted luciferase under the control of an IFN‐responsive promotor, allowing to monitor the STING‐mediated induction of the IRF pathway by a bioluminescence readout. To ensure that the luciferase signal is dependent on the activation of the STING signaling pathway, also THP1‐Dual^TM^ KO‐STING cells (InvivoGen) featuring the same reporter system were treated with bis‐HTE‐dd‐cGAMP **3**, fully confirming the dependence of IFN production on the presence of STING (Figure S4).

The obtained EC_50_ curves and data are depicted in Figure 3 and Table 1. We included the previously reported EC_50_ value of dd‐2′,3′‐cyclic adenosine monophosphate‐adenosine monophosphate (cAAMP), which is 15‐times higher than the deoxy‐version of the natural ligand cGAMP.^[21]^ However, the parent compound dd‐cGAMP **2** still has a poor EC_50_ value of 5 μM which shows that it is barely able to elicit an immune response *in cellulo* and presumably not *in vivo* either. To our delight, however, we found that bis‐HTE‐protected dd‐cGAMP **3** shows an outstanding EC_50_ value of 25 nM. It is a surprise that the large HTE protecting groups do not hamper cellular uptake and further that they are cleaved so efficiently inside the cell.

**Figure 3:**
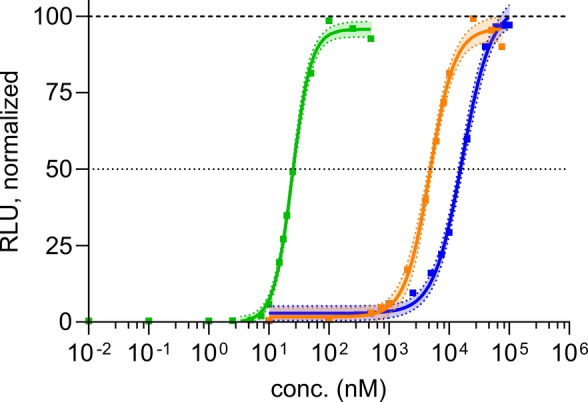
Dose‐dependent response of THP1‐Dual^TM^ cells to dd‐cGAMP **2** (orange) and bis‐HTE‐dd‐cGAMP **3** (green) in comparison to the natural STING ligand cGAMP **1** (blue). Dots represent the mean of at least three biologically independent experiments, the shade represents the 95 % confidence interval (CI).

**Table 1:** EC_50_ values of dinucleotides **1**–**3** and the previously reported cGAMP analog dd‐cAAMP.^[21]^ EC_50_ values represent the mean of at least three biologically independent experiments in THP1‐Dual^TM^ monocytic reporter cells.

Compound	EC50 (nM)
cGAMP (**1**)	16619±2296
dd‐cAAMP	74400±4600^[21]^
dd‐cGAMP (**2**)	4722±492
Bis‐HTE‐dd‐cGAMP (**3**)	24.6±1.2

To gain a more comprehensive understanding of the global effects of our new compound on immune cells, we treated the unmodified parent cell line THP1 for 18 h with bis‐HTE‐protected dd‐cGAMP **3** before conducting a proteomics analysis (Figure 4).

**Figure 4:**
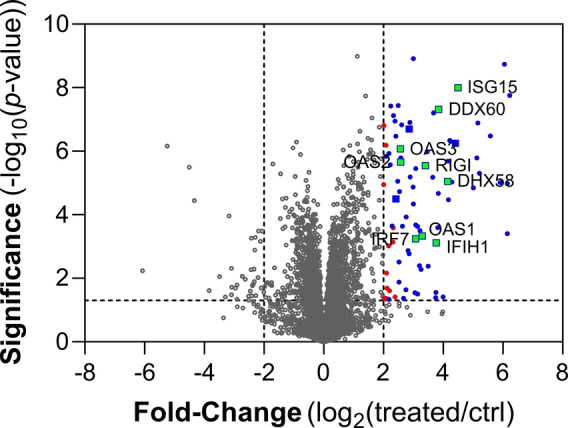
A volcano plot illustrating differentially expressed proteins in THP1 cells treated with bis‐HTE‐dd‐cGAMP **3** for 18 h compared to untreated cells (*n*=4). Proteins with significant upregulation (cut‐off *p*‐value 0.05 and fold‐change >2) are highlighted in red. DAVID analysis identified 3 highly enriched clusters within this group. Cluster‐specific proteins are depicted as follows: proteins in cluster 1 are represented by blue dots, those in both cluster 1 and 2 by blue rectangles, and proteins present in all 3 clusters are labeled and shown as green rectangles with blue border.

The significant upregulation of proteins, highlighted in red, demonstrates the overall impact of our treatment on the cellular proteome. To further investigate the biological processes affected by the treatment, we performed a functional annotation clustering using the Database for Annotation, Visualization and Integrated Discovery (DAVID).^[28]^ The analysis revealed that the upregulated proteins can be categorized mainly into three clusters, all of which are associated with immunological processes. Notably, with few exceptions, all upregulated proteins are linked to at least one of these clusters, highlighting the significant impact of the treatment with bis‐HTE‐protected dd‐cGAMP **3** on immune‐related pathways. A comprehensive list of the proteins within these clusters is provided in Table S1.

In summary, we report the synthesis and biological evaluation of dd‐cGAMP in which the negative charges of the linking phosphodiesters are removed by conversion into HTE‐protected phosphotriesters. This neutral compound features a strongly improved EC_50_ value, which makes it suitable for the development of immune‐stimulating agents that can help in our fight against cancer using immune‐oncological approaches. Studies to click targeting ligands to compound **3** for efficient delivery are under way.

## Conflict of Interests

The authors declare no conflict of interest.

## Supporting information

As a service to our authors and readers, this journal provides supporting information supplied by the authors. Such materials are peer reviewed and may be re‐organized for online delivery, but are not copy‐edited or typeset. Technical support issues arising from supporting information (other than missing files) should be addressed to the authors.

Supporting Information

## Data Availability

The data discussed in this publication is provided in the Supporting Information. The proteomic data has been deposited to the ProteomeXchange Consortium on PRIDE^[29]^ with the data set identifier PXD057395.
