# Supplementary material for: A Phosphotriester‐Masked Dideoxy‐cGAMP Derivative as a Cell‐Permeable STING Agonist
Source: Angew Chem Int Ed Engl. 2024 Nov 25;63(52):e202416353. doi: 10.1002/anie.202416353 (PMC11656140; doi:10.1002/anie.202416353)
Supplement: Supplementary file 1 — Supporting Information [file ANIE-63-e202416353-s001.pdf]

Angewandte Chemie
Eine Zeitschrift der Gesellschaft Deutscher Chemiker

Supporting Information

# A Phosphotriester-Masked Dideoxy-cGAMP Derivative as a Cell-Permeable STING Agonist

*A.-L. J. Halbritter, Y. V. Gärtner, J. Nabiev, F. Hernichel, G. Ganazzoli, D. Özdemir, A. Pappa, S. Veth, S. Stazzoni, M. Müller, V. Hornung, T. Carell**

# Supporting Information

# A Phosphotriester-Masked Dideoxy-cGAMP Derivative as a Cell-Permeable STING Agonist

*Anna-Lena J. Halbritter+[a], Yasmin V. Gärtner+[a], Jahongir Nabiev[a], Fabian Hernichel[a], Giacomo Ganazzoli[a], Dilara Özdemir[a], Aikaterini Pappa[a], Simon Veth[a], Samuele Stazzoni[a], Markus Müller[a], Veit Hornung[b] and Thomas Carell[a]**

[a] Dr. A.J. Halbritter, M.Sc. Y.V. Gärtner, M.Sc. J. Nabiev, M.Sc. F. Hernichel, Dr. G. Ganazzoli, Dr. D. Özdemir, Dr. A. Pappa, Dr. S. Veth, Dr. S. Stazzoni, Dr. M. Müller, Prof. Dr. T. Carell
Department of Chemistry, Institute for Chemical Epigenetics
Ludwig-Maximilians-Universität München
Butenandtstr. 5-13, 81377 Munich, Germany
http://www.carellgroup.de

[b] Prof. Dr. V. Hornung
Gene Center and Department of Biochemistry
Ludwig-Maximilians-Universität München
Feodor-Lynen-Str. 25, 81377 Munich, Germany

* Corresponding authors: Thomas.Carell@lmu.de

+ These authors contributed equally

# Table of Contents

1 Abbreviations .......... 3

2 General information and instruments .......... 4

3 Synthesis and characterization .......... 5

4 Cell culture and biological assays .......... 15

Cell culture .......... 15

*In vitro* CES1 cleavage assay .......... 15

*In cellulo* HTE cleavage .......... 17

IFN Induction Assay .......... 19

Proteomics .......... 20

5 NMR spectra of synthesized compounds .......... 25

6 Reference .......... 43

# 1 Abbreviations

| | |
|---|---|
| Aq. | aqueous |
| BTT | 5-benzylthio-1*H*-tetrazole |
| CDI | 1,1‘-carbonyldiimidazole |
| COSY | correlation spectroscopy |
| DCA | dichloroacetic acid |
| DCC | *N*,*N′*-dicyclohexylcarbodiimide |
| DIPAT | diisopropylammonium tetrazolide |
| DMAP | 4-dimethylaminopyridine |
| DMF | dimethylformamide |
| DMTrCl | 4,4'-dimethoxytritylchlorid |
| EtOAc | ethyl acetate |
| $Et_3N$ | triethylamine |
| h | hours |
| HMBC | heteronuclear multiple-bond correlation spectroscopy |
| HRMS (ESI) | high resolution mass spectrometry (electron spray ionization) |
| HSQC | heteronuclear single-quantum correlation spectroscopy |
| HTE | 5-hexynoic thioester |
| NMI | *N*-methylimidazole |
| NMR | nuclear magnetic resonance |
| Satd. | saturated |
| t | time |
| TBAF | tetra-*n*-butylammonium fluoride |
| TBHP | tert-butyl hydroperoxide |
| TBSCl | tert-butyldimethylsilyl chloride |
| THF | tetrahydrofuran |
| TLC | thin layer chromatography |
| UV | ultraviolet |

## 2 General information and instruments

Chemicals and anhydrous solvents were purchased from TCI, Sigma-Aldrich, ABCR, Carbosynth, Acros organics, Fluka and Roth without further purification. Pyridine, $CH_3CN$, $Et_3N$, $CH_2Cl_2$ THF, $Et_2O$ and DMF where freshly dried over molecular sieves (3 Å) before use. All moisture- and air-sensitive reactions were carried out in oven-dried glassware under an inert atmosphere of argon. Routine $^1H$, $^{13}C$ and $^{31}P$ NMR spectra were recorded on a Bruker Ascend 400 spectrometer (400 MHz for $^1H$ NMR, 100 MHz for $^{13}C$ NMR and 162 MHz for $^{31}P$ NMR), Bruker Ascend 500 spectrometer (500 MHz for $^1H$ NMR, 125 MHz for $^{13}C$ NMR and 202 MHz for $^{31}P$ NMR), or a Bruker Avance III HD spectrometer (800 MHz for $^1H$ NMR, 201 MHz for $^{13}C$ NMR). Chemical shifts are reported in parts per million (ppm) relative to the partially deuterated NMR solvents $CDCl_3$ (7.26 ppm for $^1H$ NMR and 77.16 ppm for $^{13}C$), $CD_2Cl_2$ (5.32 ppm for $^1H$ NMR and 53.84 ppm for $^{13}C$), and DMSO-$d_6$ (2.50 ppm $^1H$ NMR and 39.52 ppm for $^{13}C$). All coupling constants were reported in Hertz (Hz). COSY, HMQC and HMBC NMR experiments were recorded to help with the assignment of $^1H$ and $^{13}C$ signals. NMR spectra were analyzed using MestReNova version 10.0. High Resolution Mass Spectra (HRMS) were measured on a Thermo Finnigan LTQ-FT with ESI as ionization mode. Column chromatography was performed with technical grade silica gel, 40-63 µm particle size. Reaction progress was monitored by thin layer chromatography (TLC) analysis on silica gel 60 F254 and compounds were visualized under UV light and by *p*-anisaldehyde staining. The photolabile protecting group was removed with a Photoreactor M2 from Acceled (Penn Photon Devices, LLC) with a 365 nm wavelength LED module. The photoreactor was used with 100% light-power and maximum fan speed. All reactions involving light-sensitive molecules, were performed in aluminum foil wrapped glassware. Analytical RP-HPLC was performed on an Agilent 1260 Infinity II LC system with a G7165A detector equipped with a Nucleodur 100-3 C18ec column from Macherey-Nagel. A flowrate of 1 mL/min was applied. Preparative RP-HPLC was performed on an Agilent 1260 Infinity II Manual Preparative LC system with a G7114A detector equipped with a Nucleodur 100-5 C18ec column from Macherey-Nagel. A flowrate of 5 mL/min was applied.

# 3 Synthesis and characterization

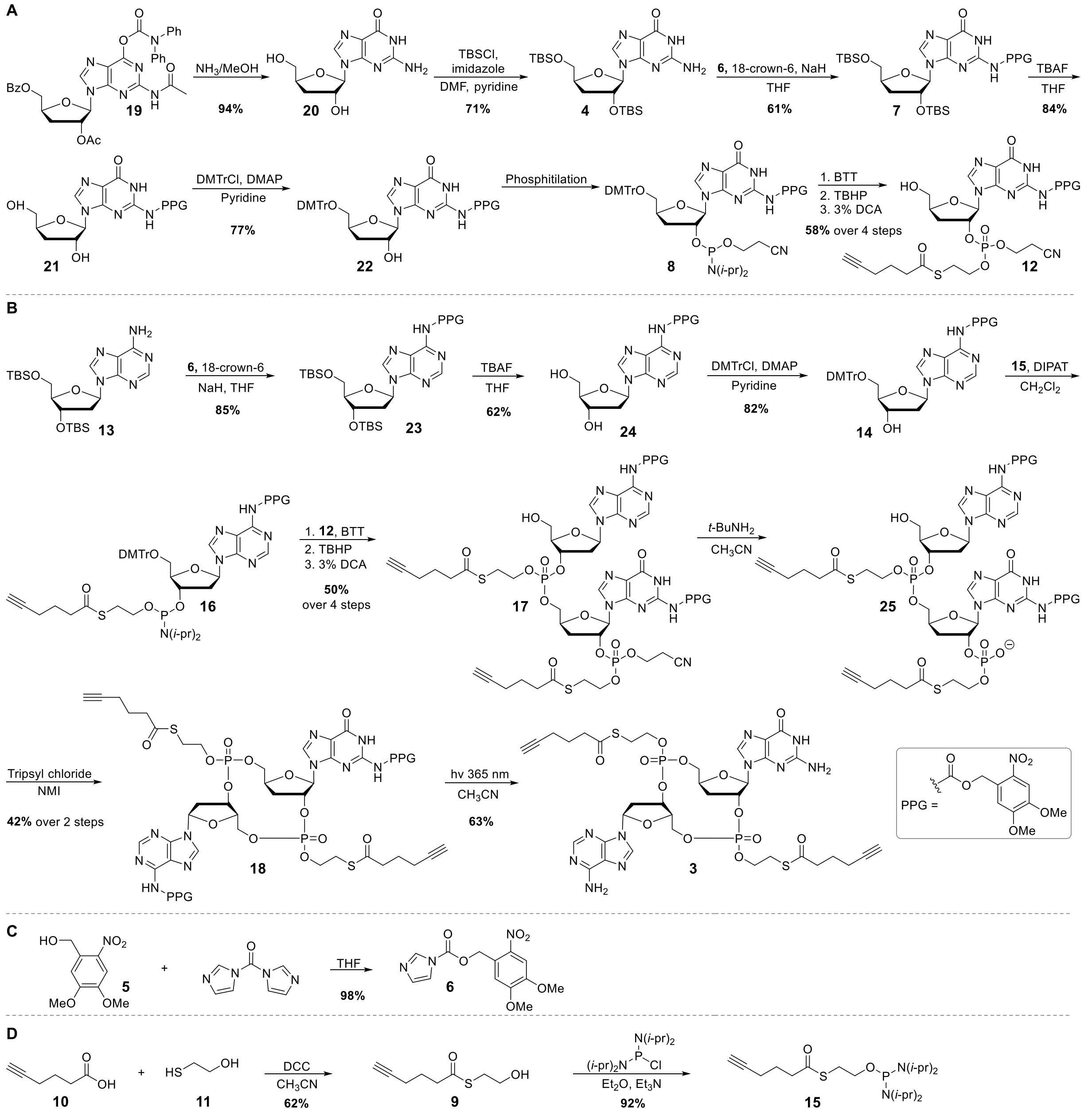


**Scheme S1**. A) Synthesis of 3'-deoxyguanosine triphosphoester **12**. B) Synthesis of bis-HTE-dd-cGAMP **3**. C) Synthesis of the photolabile protecting group **6**. D) Synthesis of the HTE-protecting reagents **9** and **15**. PPG = photolabile protecting group. Tripsyl chloride = 2,4,6-triisopropylbenzenesulfonyl chloride.

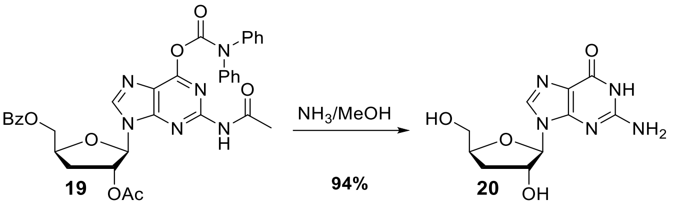

Compound **19** was synthesized according to published literature procedures.[1-4] A solution of compound **19** (2.78 g, 4.27 mmol) in $NH_3$/MeOH (113 mL, 7 N) was prepared and stirred in a pressure tube at 80 °C for 4 h. The solution was cooled to rt and concentrated in *vacuo*. The residue was washed with $CH_2Cl_2$ (60 mL) under sonication for 2 min, after which the solvent was decanted. This procedure was repeated three times to yield compound **20** (1.07 g, 4.02 mmol, 94%) as a white solid. $R_f$=0.10 (MeOH:$CH_2Cl_2$ 20:80); $^1$H NMR (500 MHz, DMSO-d$_6$, ppm) δ=8.90 (bs, 1H), 7.92 (s, 1H), 6.50 (s, 2H), 5.67 (d, *J* = 2.2 Hz, 1H), 5.58 (s, 1H), 5.00 (s, 1H), 4.42 (dt, *J* = 5.4, 2.5 Hz, 1H), 4.28 (ddd, *J* = 9.6, 6.0, 3.2 Hz, 1H), 3.64 (dd, *J* = 11.9, 3.5 Hz, 1H), 3.49 (dd, *J* = 11.9, 4.2 Hz, 1H), 2.20 (ddd, *J* = 13.1, 9.1, 5.7 Hz, 1H), 1.87 (ddd, *J* = 13.1, 6.2, 2.8 Hz, 1H); $^{13}$C NMR (126 MHz, DMSO-d$_6$ ppm) δ=156.99, 153.76, 150.80, 135.20, 116.63, 89.89, 80.54, 74.93, 62.55, 34.38; HRMS (ESI): *m/z* calcd for $C_{10}H_{13}N_5O_4$+$H^+$ 268.1040 $[M+H]^+$; found 268.1037.

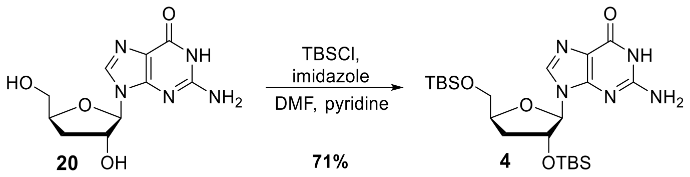

To a solution of **20** (1.07 g, 4.02 mmol) in DMF (38 mL) and pyridine (38 mL) were added TBSCl (2114 mg, 14.07 mmol) and imidazole (958 mg, 14.07 mmol). The resulting solution was stirred at rt for 2 d. $H_2O$ (100 mL) and $CH_2Cl_2$ (100 mL) were added, the organic phase separated and washed with satd. aq. $NaHCO_3$ (4 x 150 mL). The organic phase was dried over $Na_2SO_4$, concentrated *in vacuo* and purified by flash column chromatography using a gradient elution (MeOH:$CH_2Cl_2$; 2:98 to 6:94), yielding **4** (1410 mg, 2.84 mmol, 71%) as a white foam. $R_f$=0.50 (MeOH:$CH_2Cl_2$ 10:90); $^1$H NMR (500 MHz, $CDCl_3$, ppm) δ=12.21 (bs, 1H), 7.99 (s, 1H), 5.74 (s, 1H), 4.56 – 4.52 (m, 1H), 4.44 (s, 1H), 4.16 (s, 1H), 3.77 (dd, *J* = 11.6, 2.4 Hz, 1H), 2.17 – 2.11 (m, 1H), 1.73 – 1.70 (m, 1H), 0.95 (s, 9H), 0.92 (s, 9H), 0.21 (s, 3H), 0.14 (s, 3H), 0.12 (s, 3H), 0.11 (s, 3H); $^{13}$C NMR (126 MHz, $CDCl_3$, ppm) δ=166.55, 159.41, 153.75, 150.62, 135.23, 129.82, 128.65, 116.81, 91.87, 81.53, 77.79, 77.42, 63.41, 33.04, 26.17, 25.88, 18.66, 18.11, -4.33, -4.85, -5.20, -5.35; HRMS (ESI): *m/z* calcd for $C_{22}H_{41}N_5O_4Si_2$+$H^+$: 496.2770 $[M+H]^+$; found: 496.2759.

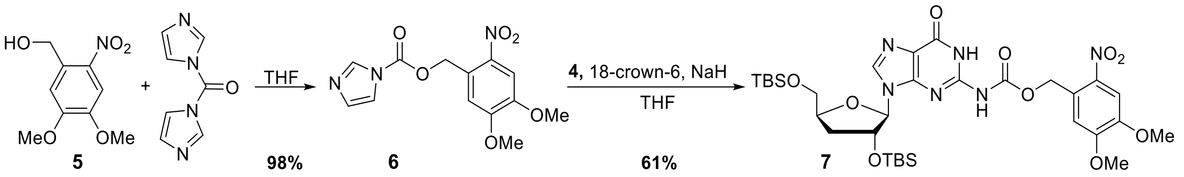

A suspension of 1,1'-carbonyldiimidazole (CDI) (558 mg, 3.44 mmol) and 4,5-dimethoxy-2-nitrobenzyl alcohol **5** (734 mg, 3.44 mmol) in THF (8.1 mL) was stirred at 0 °C for 1 h and further at rt for 30 min. *i*-Hexane (10 mL) was added, the solid was filtered and washed with THF:*i*-hexane (50:50, 50 mL) and dried *in vacuo* to yield **6** (1037 mg, 3.38 mmol, 98%) as a white solid and can be used without further purification. To a solution of compound **4** (325 mg, 0.66 mmol) in dry THF (6.5 mL) was added 18-crown-6 (433 mg, 1.64 mmol), the solution was cooled to 0 °C and NaH (60% in mineral oil, 56 mg, 2.73 mmol) was added. The solution was stirred at 0 °C for 10 min. Compound **6** (565 mg, 1.84 mmol) was added, and the solution stirred for 14 h, allowing it to slowly warm-up to rt. The solution was filtered over celite and diluted with $CH_2Cl_2$ (25 mL). The solution was washed with satd. aq. $NaHCO_3$ (3 x 30 mL), dried over $Na_2SO_4$, concentrated *in vacuo* and purified by flash column chromatography using a gradient elution (MeOH:$CH_2Cl_2$; 0.5:99.5 to 2:98), yielding **7** (290 mg, 0.39 mmol, 61%) as a light-yellow foam. $R_f$=0.50 (MeOH:$CH_2Cl_2$ 5:95); $^1$H NMR (500 MHz, $CDCl_3$, ppm) δ=11.24 (s, 1H), 8.19 (s, 1H), 8.14 (s, 1H), 7.71 (s, 1H), 7.05 (s, 1H), 5.77 (d, *J* = 1.7 Hz, 1H), 5.60 (d, *J* = 2.0 Hz, 2H), 4.48 (ddt, *J* = 8.7, 5.6, 2.6 Hz, 1H), 4.41 (dt, *J* = 4.8, 2.3 Hz, 1H), 4.04 (dd, *J* = 11.6, 2.6 Hz, 1H), 4.00 (s, 3H), 3.96 (s, 3H), 3.73 (dd, *J* = 11.6, 2.7 Hz, 1H), 2.27 (ddd, *J* = 13.0, 9.2, 5.0 Hz, 1H), 1.85 (ddd, *J* = 13.0, 5.9, 2.7 Hz, 1H), 0.91 (s, 9H), 0.84 (s, 9H), 0.10 (s, 3H), 0.09 (s, 3H), 0.02 (s, 6H); $^{13}$C NMR (126 MHz, $CDCl_3$, ppm) δ=155.76, 153.66, 153.32, 149.08, 147.85, 146.18, 140.47, 137.29, 124.83, 121.22, 112.22, 108.49, 91.31, 81.21, 77.86, 65.74, 63.89, 56.82, 56.59, 33.97, 26.12, 25.72, 18.64, 18.03, -4.65, -4.82, -5.24, -5.36; HRMS (ESI): *m/z*: calcd for $C_{32}H_{50}N_6O_{10}Si_2+Na^+$: 757.3019 $[M+Na]^+$; found: 757.3007.

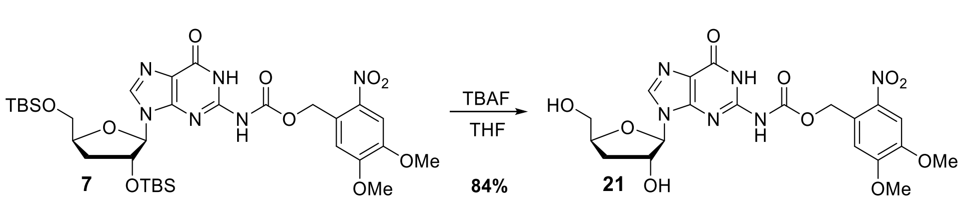

To a solution of **7** (952 mg, 1.30 mmol) in THF (25 mL) was added TBAF (3.9 mL, 3.89 mmol, 1.0 M in THF) and the reaction stirred at rt for 14 h. Dowex-50 (2372 mg) and $CaCO_3$ (791 mg, 7.90 mmol) were added, the mixture was stirred for 15 min, filtered through celite, washed with MeOH (100 mL) and concentrated *in vacuo*. The residue was purified by flash column chromatography using a gradient elution (MeOH:$CH_2Cl_2$; 5:95-10:90), to yield **21** (550 mg, 1.09 mmol, 84%) as a light-yellow solid. $R_f$=0.20 (MeOH:$CH_2Cl_2$ 10:90); $^1$H NMR (500 MHz, DMSO-$d_6$ ,ppm) δ=11.78 (s, 1H), 11.32 (s, 1H), 8.24 (s, 1H), 7.74 (s, 1H), 7.38 (s, 1H), 5.77 (d, *J* = 2.0

Hz, 1H), 5.62 (d, $J$ = 4.2 Hz, 1H), 5.57 (s, 2H), 5.01 (t, $J$ = 5.4 Hz, 1H), 4.49 (ddt, $J$ = 6.1, 4.5, 2.4 Hz, 1H), 4.33 (ddt, $J$ = 9.6, 6.1, 3.8 Hz, 1H), 3.96 (s, 3H), 3.88 (s, 3H), 3.67 (ddd, $J$ = 11.9, 5.5, 3.4 Hz, 1H), 3.54 – 3.50 (m, 1H), 2.25 (ddd, $J$ = 13.3, 9.3, 5.6 Hz, 1H), 1.90 (ddd, $J$ = 13.2, 6.1, 2.6 Hz, 1H); $^{13}$C NMR (126 MHz, DMSO-d$_6$, ppm) δ=155.12, 154.10, 153.59, 148.48, 147.90, 147.22, 139.01, 137.42, 126.16, 119.91, 110.58, 108.15, 90.22, 80.96, 75.14, 64.42, 62.32, 56.52, 56.12, 34.23; HRMS (ESI): *m/z* calcd for $C_{20}H_{21}N_6O_{10}$-H$^-$: 505.1325 (M-H)$^-$; found: 505.1320.

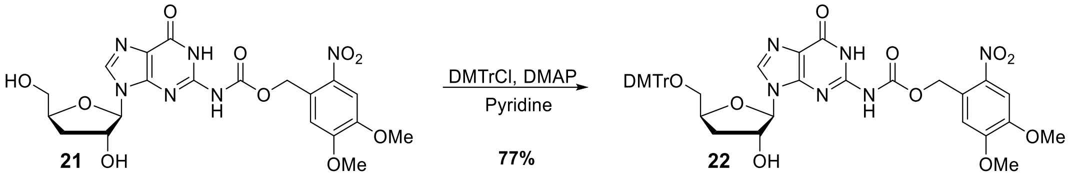


Toluene (3 x 5 mL) was evaporated from **21** (525 mg, 1.04 mmol), followed by sequential addition of pyridine (24 mL), DMTrCl (529 mg, 1.56 mmol) and DMAP (13 mg, 0.10 mmol). The solution was stirred at rt for 2 d, the solvent removed *in vacuo* and the residue was purified by flash column chromatography using a gradient elution (MeOH:$CH_2Cl_2$:$Et_3N$; 0.5:99.5:0.1 to 2.5:97.5:0.1) to yield **22** (347 mg, 0.80 mmol, 77%) as a light-yellow solid. $R_f$=0.50 (MeOH:$CH_2Cl_2$ 5:95); $^1$H NMR (500 MHz, $CD_2Cl_2$, ppm) δ=7.84 (s, 1H), 7.66 (s, 1H), 7.34 (dt, $J$ = 6.3, 1.4 Hz, 3H), 7.24 – 7.21 (m, 6H), 7.19 – 7.15 (m, 1H), 6.78 – 6.75 (m, 4H), 5.71 – 5.70 (m, 1H), 5.60 – 5.54 (m, 2H), 4.80 (td, $J$ = 7.1, 4.1 Hz, 1H), 4.59 (p, $J$ = 4.1 Hz, 1H), 3.87 (d, $J$ = 3.2 Hz, 6H), 3.74 (d, $J$ = 1.0 Hz, 6H), 3.31 (dd, $J$ = 10.4, 3.3 Hz, 1H), 3.14 (dd, $J$ = 10.4, 4.4 Hz, 1H), 2.28 – 2.16 (m, 2H); $^{13}$C NMR (126 MHz, $CD_2Cl_2$, ppm) δ=158.99, 154.68, 151.52, 149.79, 148.51, 145.19, 139.45, 136.36, 136.23, 136.18, 130.36, 128.46, 128.32, 128.14, 127.14, 120.40, 113.39, 110.33, 108.31, 94.02, 86.64, 80.29, 76.41, 66.08, 64.09, 57.24, 56.60, 55.55, 35.16; HRMS (ESI): *m/z* calcd for $C_{41}H_{41}N_6O_{12}$+H$^+$: 809.2777 [M+H]$^+$; found: 809.2777.

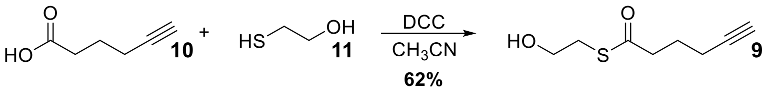


To a solution of 5-hexynoic acid (**10**) (2.95 mL, 26.75 mmol) in $CH_3CN$ (250 mL) at 0 °C was added mercaptoethanol (**11**) (2.82 mL, 40.13 mmol) and *N*,*N′*-dicyclohexylcarbodiimide (DCC) (5520 mg, 26.75 mmol) and the reaction was stirred at rt overnight, allowing the ice-bath to slowly warm-up. The white precipitate that had formed was filtered off and the solution was concentrated *in vacuo*. The residue was purified by flash column chromatography using a gradient elution (EtOAc:*i*-hexane; 10:90-20:80), to yield **9** (2850 mg, 16.54 mmol, 62%) as an oil. $R_f$=0.50 (EtOAc:*i*-hexane 20:80); $^1$H NMR (500 MHz, $CDCl_3$, ppm) δ=3.76 (q, $J$ = 5.8 Hz, 2H), 3.09 (t, $J$ = 6.1 Hz, 2H), 2.73 (t, $J$ = 7.5 Hz, 2H), 2.26 (td, $J$ = 6.9, 2.6 Hz, 2H), 2.03 (t, $J$ =

5.8 Hz, 1H), 1.98 (t, $J$ = 2.6 Hz, 1H), 1.88 (p, $J$ = 7.1 Hz, 2H); $^{13}C$ NMR (126 MHz, $CDCl_3$, ppm) δ=199.36, 83.08, 69.57, 61.93, 42.71, 31.96, 24.20, 17.86; HRMS (ESI): *m/z* calcd for $C_8H_{12}O_2S+H^+$: 173.0631 $[M+H]^+$; found: 173.0630.

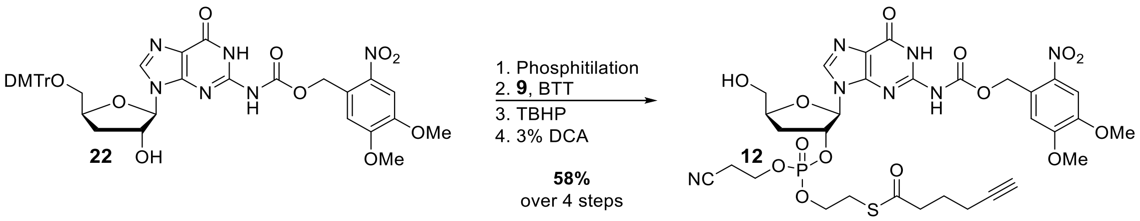


A solution of **22** (381 mg, 0.47 mmol) in $CH_2Cl_2$ (5.2 mL) was treated with diisopropyl ammonium tetrazolide (DIPAT) (105 mg, 0.61 mmol) and 2-cyanoethyl *N,N,N',N'*-tetraisopropylphosphorodiamidite (195 µL, 0.61 mmol). The reaction was stirred at rt overnight. The solution was diluted with $CH_2Cl_2$ (20 mL) and washed with satd. aq. $NaHCO_3$ (3 x 50 mL), dried over $Na_2SO_4$ and concentrated *in vacuo*. The product (**8**) was used directly for the next step without further purification.

To a solution of 5-(benzylthio)-1*H*-tetrazole (BTT) (226 mg, 1.18 mmol) in $CH_3CN$ (2 mL) was added compound **9** (163 mg, 1.18 mmol) and **8** in $CH_3CN$ (2 mL) and the solution was stirred at rt for 2 h. *t*-Butyl hydroperoxide (TBHP) (271 µL, 1.63 mmol, 6.0 M in decane) was added and the solution stirred for 30 min. The solution was diluted with EtOAc (50 mL), washed with satd. aq. $NaHCO_3$ (3 x 50 mL) and brine (50 mL), dried over $Na_2SO_4$ and concentrated *in vacuo*. The residue was dissolved in $CH_2Cl_2$ (17.9 mL), DCA (544 µL, 6.60 mmol) was added dropwise and the solution stirred for 10 min. The solution was neutralized to pH 7 by addition of satd. aq. $NaHCO_3$. The solution was diluted with $CH_2Cl_2$ (20 mL) and the phases separated. The organic phase was washed with satd. aq. $NaHCO_3$ (3 x 50 mL), dried over $Na_2SO_4$, concentrated *in vacuo* and purified by flash column chromatography using a gradient elution ($MeOH:CH_2Cl_2$; 1:99 to 4:96) yielding **12** (217 mg, 0.27 mmol, 58%) as an inseparable mixture of two diastereomers. $R_f$=0.20 ($MeOH:CH_2Cl_2$ 5:95); $^1H$ NMR (500 MHz, $CD_2Cl_2$, ppm) δ=11.30 (s, 2H), 10.34 (d, $J$ = 54.3 Hz, 2H), 8.12 (d, $J$ = 8.0 Hz, 2H), 7.67 (d, $J$ = 2.0 Hz, 2H), 7.11 (d, $J$ = 5.9 Hz, 2H), 6.01 (s, 2H), 5.57 (s, 4H), 4.67 (s, 2H), 4.50 (ddd, $J$ = 9.1, 5.9, 2.7 Hz, 2H), 4.30 – 4.23 (m, 4H), 4.17 – 4.12 (m, 4H), 4.05 – 4.01 (m, 2H), 3.94 (d, $J$ = 5.3 Hz, 6H), 3.90 (s, 6H), 3.74 – 3.70 (m, 2H), 3.19 – 3.11 (m, 4H), 2.83 – 2.79 (m, 4H), 2.68 (t, $J$ = 7.4 Hz, 4H), 2.62 – 2.55 (m, 2H), 2.26 – 2.19 (m, 6H), 2.04 – 2.02 (m, 2H), 1.84 – 1.78 (m, 4H); $^{13}C$ NMR (126 MHz, $CD_2Cl_2$, ppm) δ=198.58, 198.22, 155.86, 154.35, 154.31, 154.26, 154.22, 148.99, 148.95, 148.27, 147.54, 140.04, 139.96, 138.33, 126.02, 125.92, 121.02, 121.00, 117.44, 117.30, 111.33, 111.16, 108.50, 90.34, 90.28, 90.21, 83.35, 83.33, 82.39, 82.36, 82.01, 69.63, 67.08, 67.03, 65.60, 63.19, 63.15, 63.12, 63.08, 62.35, 56.99, 56.97, 56.68, 42.87, 42.85, 32.48, 29.10, 29.06, 29.04, 29.01, 24.42, 24.40, 20.09, 20.07, 20.03, 20.01, 17.90; $^{31}P$ NMR

(202 MHz, $CD_2Cl_2$, ppm) δ=0.05, 0.00; HRMS (ESI): *m/z* calcd for $C_{31}H_{36}N_7O_{14}PS+Na^+$: 816.1676 $[M+Na]^+$; found: 816.1671.

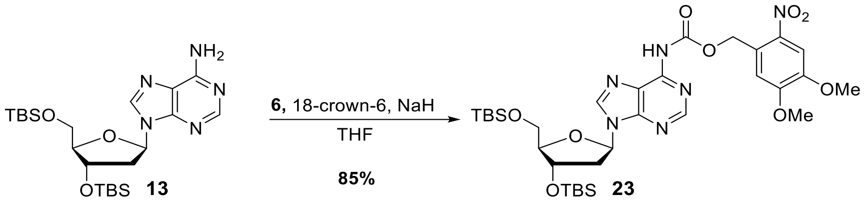


To a solution of compound **13** (200 mg, 0.42 mmol) in dry THF (4.2 mL) was added 18-crown-6 (253 mg, 0.96 mmol), the solution was cooled to 0 °C and NaH (60% in mineral oil, 39 mg, 1.60 mmol) was added. The solution was stirred at 0 °C for 10 min. Compound **6** (333 mg, 1.84 mmol) was added, and the solution stirred for 24 h, allowing it to slowly warm-up to rt. The solution was filtered over celite and diluted with $CH_2Cl_2$ (20 mL). The solution was washed with satd. aq. $NaHCO_3$ (3 x 30 mL), dried over $Na_2SO_4$, concentrated *in vacuo* and purified by flash column chromatography using a gradient elution ($MeOH:CH_2Cl_2$; 0.5:99.5 to 2:98), yielding **23** (254 mg, 0.35 mmol, 85%) as a light-yellow foam. $R_f$=0.60 ($MeOH:CH_2Cl_2$ 5:95); $^1H$ NMR (500 MHz, $CDCl_3$, ppm) δ=8.74 (s, 1H), 8.66 (s, 1H), 8.33 (s, 1H), 7.72 (s, 1H), 7.24 (s, 1H), 6.49 (t, *J* = 6.4 Hz, 1H), 5.72 (s, 2H), 4.61 (dt, *J* = 5.8, 3.6 Hz, 1H), 4.03 (q, *J* = 3.4 Hz, 1H), 3.98 (s, 3H), 3.96 (s, 3H), 3.87 (dd, *J* = 11.2, 4.1 Hz, 1H), 3.77 (dd, *J* = 11.2, 3.1 Hz, 1H), 2.65 (ddd, *J* = 12.7, 6.7, 5.8 Hz, 1H), 2.47 (ddd, *J* = 13.1, 6.2, 3.9 Hz, 1H), 0.91 (s, 9H), 0.89 (s, 9H), 0.10 (s, 6H), 0.07 (d, *J* = 2.8 Hz, 6H); $^{13}C$ NMR (126 MHz, $CDCl_3$, ppm) δ=153.71, 152.72, 151.02, 150.98, 149.17, 148.51, 141.70, 140.09, 126.82, 122.56, 111.23, 108.33, 88.22, 84.78, 71.98, 68.10, 64.91, 62.85, 56.72, 56.56, 41.52, 26.06, 25.87, 18.54, 18.13, -4.53, -4.68, -5.28, -5.36; HRMS (ESI): *m/z* calcd for $C_{32}H_{50}N_6O_9Si_2+H^+$: 719.3251 $[M+H]^+$; found: 719.3241.

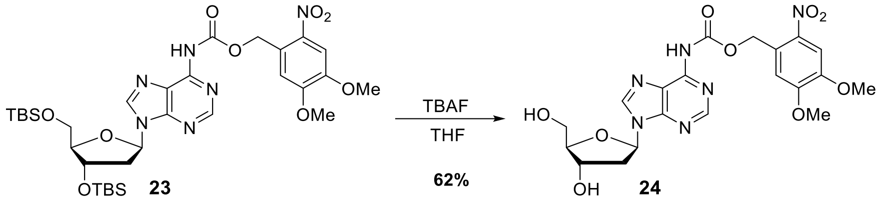


To a solution of **23** (1760 mg, 2.45 mmol) in THF (47 mL) was added TBAF (7.4 mL, 7.34 mmol, 1.0 M in THF) and the reaction stirred at rt for overnight. Dowex-50 (4484 mg) and $CaCO_3$ (1495 mg, 14.93 mmol) were added, the mixture was stirred for 15 min, filtered through celite, washed with MeOH (200 mL) and concentrated *in vacuo*. The residue was purified by flash column chromatography using a gradient elution ($MeOH:CH_2Cl_2$; 5:95-6:94), to yield **24** (927 mg, 1.51 mmol, 62%) as a light-yellow solid. $R_f$=0.20 ($MeOH:CH_2Cl_2$ 5:95); $^1H$ NMR (500 MHz, DMSO-$d_6$, ppm) δ=10.91 (s, 1H), 8.68 (s, 1H), 8.66 (s, 1H), 7.73 (s, 1H), 7.48 (s, 1H), 6.45 (t,

$J$ = 6.8 Hz,1H), 5.54 (s, 2H), 5.36 (d, $J$ = 4.2 Hz, 1H), 5.02 (t, $J$ = 5.6 Hz, 1H), 4.46 – 4.43 (m, 1H), 3.95 (s, 3H), 3.91 – 3.89 (m, 1H), 3.88 (s, 3H), 3.63 (dt, $J$ = 11.8, 5.0 Hz, 1H), 3.53 (ddd, $J$ = 11.7, 5.9, 4.5 Hz, 1H), 2.80 – 2.75 (m, 1H), 2.35 (ddd, $J$ = 13.3, 6.3, 3.4 Hz, 1H); $^{13}$C NMR (126 MHz, DMSO-d$_6$, ppm) δ=153.61, 151.70, 151.68, 151.52, 149.53, 147.75, 142.85, 139.03, 127.28, 123.73, 110.61, 108.12, 88.02, 83.79, 70.70, 63.63, 61.62, 56.52, 56.11; HRMS (ESI): *m/z* calcd for $C_{20}H_{22}N_6O_9$+Na$^+$: 513.1346 [M+Na]$^+$; found: 513.1334.

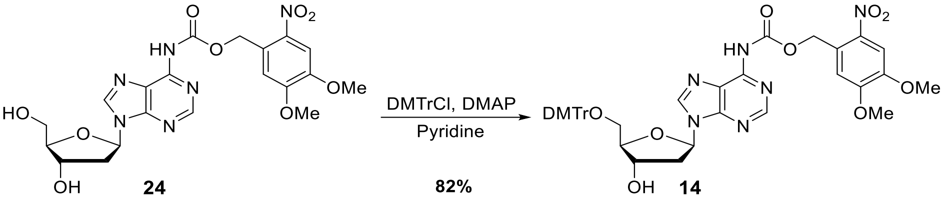


Toluene (3 x 5 mL) was evaporated from **24** (648 mg, 1.32 mmol), followed by sequential addition of pyridine (30 mL), DMTrCl (671 mg, 1.98 mmol) and DMAP (16 mg, 0.13 mmol). The solution was stirred at rt for 2 d, the solvent removed *in vacuo* and the residue was purified by flash column chromatography using a gradient elution (MeOH:$CH_2Cl_2$:$Et_3N$; 0.5:99.5:0.1 to 1.3:98.7:0.1) to yield **14** (855 mg, 1.08 mmol, 82%) as a light-yellow solid. $R_f$=0.50 (MeOH:$CH_2Cl_2$ 5:95); $^1$H NMR (500 MHz, $CD_2Cl_2$, ppm) δ=8.83 (s, 1H), 8.60 (s, 1H), 8.14 (s, 1H), 7.71 (s, 1H), 7.39 – 7.36 (m, 2H), 7.28 – 7.21 (m, 7H), 7.20 – 7.16 (m, 1H), 6.79 – 6.75 (m, 4H), 6.46 (t, $J$ = 6.5 Hz, 1H), 5.66 (s, 2H), 4.72 (dt, $J$ = 6.1, 4.0 Hz, 1H), 4.16 (td, $J$ = 4.6, 3.5 Hz, 1H), 3.92 (s, 3H), 3.91 (s, 3H), 3.74 (s, 3H), 3.74 (s, 3H), 3.40 – 3.34 (m, 2H), 2.88 (dt, $J$ = 13.6, 6.3 Hz, 1H), 2.55 (ddd, $J$ = 13.5, 6.4, 4.1 Hz, 1H); $^{13}$C NMR (126 MHz, $CD_2Cl_2$, ppm) δ=159.03, 154.30, 152.70, 151.22, 151.14, 149.51, 148.81, 145.10, 142.07, 140.06, 136.00, 135.99, 130.36, 130.33, 128.38, 128.20, 127.25, 127.23, 122.91, 113.44, 111.02, 108.56, 86.86, 86.73, 85.10, 72.63, 65.01, 64.10, 56.87, 56.68, 55.57, 40.46; HRMS (ESI): *m/z* calcd for $C_{41}H_{40}N_6O_{11}$+K$^+$: 831.2387 [M+K]$^+$; found: 831.2368.

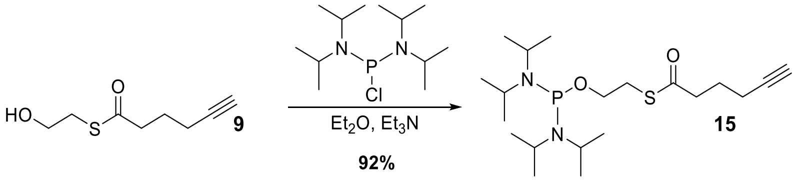


To a solution of **9** (500 mg, 2.91 mmol) in $Et_2O$ (11 mL) was added at 0 °C $Et_3N$ (810 µL, 5.81 mmol) and bis(diisopropylamino)chlorophosphine (846 mg, 3.18 mmol) and the solution was stirred at rt for 18 h. The suspension was diluted with $Et_3N$:$Et_2O$ (4:96, 40 mL), the precipitate filtered away and the solution concentrated *in vacuo*. The residue was purified by flash column chromatography ($Et_3N$: *i*-hexane, 6:94) to yield phosphor reagent **15** (1072 mg, 2.66 mmol, 92%) as a colorless oil. $R_f$=0.40 (EtOAc:*i*-hexane 40:60); $^1$H NMR (500 MHz, $CD_2Cl_2$, ppm) δ=3.68 – 3.61 (m, 2H), 3.57 – 3.46 (m, 4H), 3.11 (t, $J$ = 6.4 Hz, 2H), 2.68 (t, $J$ = 7.4 Hz, 2H),

2.24 (td, $J$ = 7.0, 2.7 Hz, 2H), 2.01 (t, $J$ = 2.6 Hz, 1H), 1.85 (p, $J$ = 7.2 Hz, 2H), 1.15 (dd, $J$ = 6.9, 4.8 Hz, 24H); $^{13}$C NMR (126 MHz, $CD_2Cl_2$, ppm) δ=198.70, 83.57, 69.35, 63.38, 63.20, 44.85, 44.75, 42.96, 31.13, 31.06, 24.78, 24.72, 24.70, 24.01, 23.97, 18.06; $^{31}$P NMR (202 MHz, $CD_2Cl_2$, ppm) δ=124.10.

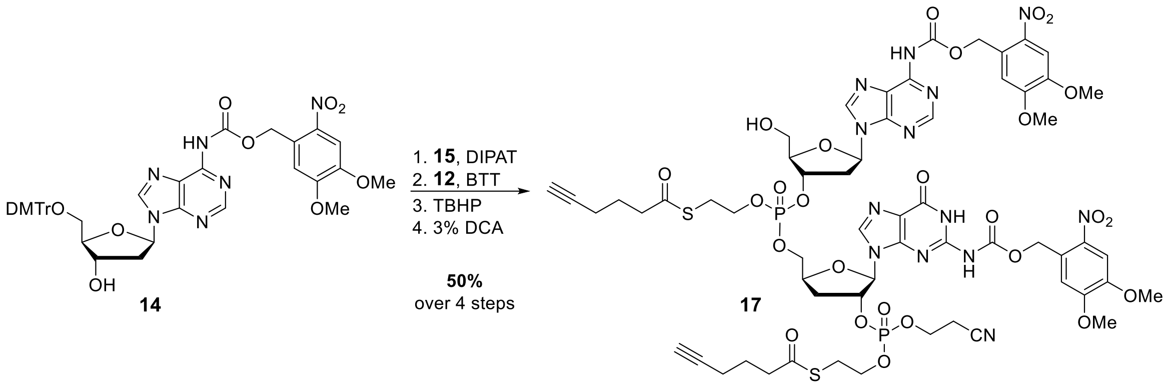


A solution of **14** (110 mg, 0.14 mmol) in $CH_2Cl_2$ (1.4 mL) was treated with DIPAT (24 mg, 1.39 mmol) and phosphor reagent **15** (95 mg, 0.24 mmol). The reaction was stirred at rt overnight. The solution was diluted with $CH_2Cl_2$ (20 mL) and washed with satd. aq. $NaHCO_3$ (3 x 30 mL), dried over $Na_2SO_4$ and concentrated *in vacuo*. The product (**16**) was used directly for the next synthetic step without further purification.

To a solution of 5-(benzylthio)-1*H*-tetrazole (BTT) (80 mg, 0.42 mmol) in $CH_3CN$ (1 mL) was added **16** in $CH_3CN$ (1 mL) and compound **12** (50 mg, 0.06 mmol) and the solution was stirred at rt for 2 h. TBHP (76 µL, 0.46 mmol, 6.0 M in decane) was added and the solution stirred for 30 min. The solution was diluted with EtOAc (20 mL), washed with satd. aq. $NaHCO_3$ (3 x 20 mL) and brine (20 mL), dried over $Na_2SO_4$ and concentrated *in vacuo*. The residue was dissolved in $CH_2Cl_2$ (4.3 mL), DCA (133 µL, 1.61 mmol) was added dropwise and the solution stirred for 10 min. The solution was neutralized to pH 7 by addition of satd. aq. $NaHCO_3$. The solution was diluted with $CH_2Cl_2$ (20 mL) and the phases separated. The organic phase was washed with satd. aq. $NaHCO_3$ (3 x 25 mL), dried over $Na_2SO_4$, concentrated *in vacuo* and purified by flash column chromatography using a gradient elution (MeOH:$CH_2Cl_2$; 2:98 to 4.5:95.5) yielding **17** (71 mg, 0.05 mmol, 75%) as an inseparable mixture of 4 diastereomers. $R_f$=0.30 (MeOH:$CH_2Cl_2$ 6:94); $^1$H NMR (500 MHz, $CDCl_3$, ppm) δ=11.37 (s, 1H), 10.16 (d, $J$ = 44.6 Hz, 1H), 9.00 (d, $J$ = 26.2 Hz, 1H), 8.71 – 8.64 (m, 1H), 8.55 – 8.50 (m, 1H), 7.89 – 7.87 (m, 1H), 7.72 – 7.69 (m, 2H), 7.28 (m, 1H), 7.09 (s, 1H), 6.64 – 6.59 (m, 1H), 6.09 – 6.07 (m, 1H), 5.97 – 5.91 (s, 1H), 5.71 – 5.70 (m, 2H), 5.62 – 5.58 (m, 2H), 5.44 – 5.43 (m, 1H), 5.31 – 5.28 (m, 1H), 4.73 – 4.71 (m, 1H), 4.57 – 4.54 (m, 1H), 4.39 – 4.34 (m, 2H), 4.31 – 4.26 (m, 2H), 4.21 – 4.10 (m, 4H), 4.03 – 3.98 (m, 6H), 3.97 – 3.94 (m, 6H), 3.90 – 3.84 (m, 1H), 3.80 – 3.76 (m, 1H), 3.23 – 3.12 (m, 4H), 3.06 – 3.01 (m, 1H), 2.82 – 2.78 (m, 2H), 2.76 – 2.61 (m, 5H), 2.55 – 2.49 (m, 1H), 2.46 – 4.37 (m, 1H), 2.27 – 2.21 (m, 4H), 2.00 – 1.97 (m, 2H), 1.90 – 1.85 (m, 4H); $^{13}$C NMR (126 MHz, $CDCl_3$, ppm) δ=198.04, 197.73, 197.58, 155.41, 153.79,

153.61, 151.85, 149.92, 148.60, 148.27, 147.54, 147.10, 143.56, 139.71, 126.61, 123.32, 116.50, 116.39, 110.86, 108.10, 87.42, 86.98, 82.72, 82.66, 77.16, 76.90, 76.65, 69.58, 69.56, 69.53, 66.63, 66.54, 65.31, 64.76, 62.78, 62.54, 56.66, 56.58, 56.34, 56.32, 42.45, 28.69, 28.51, 23.85, 23.81, 19.67, 17.58, 17.55; $^{31}$P NMR (202 MHz, $CDCl_3$, ppm) δ=0.13, 0.12, 0.02, -0.00, -0.82, -0.94, -1.15, -1.26; HRMS (ESI): *m/z* calcd for $C_{59}H_{67}N_{13}O_{26}P_2S_2$+$Na^+$: 1522.3125 $[M+Na]^+$; found: 1522.3090.

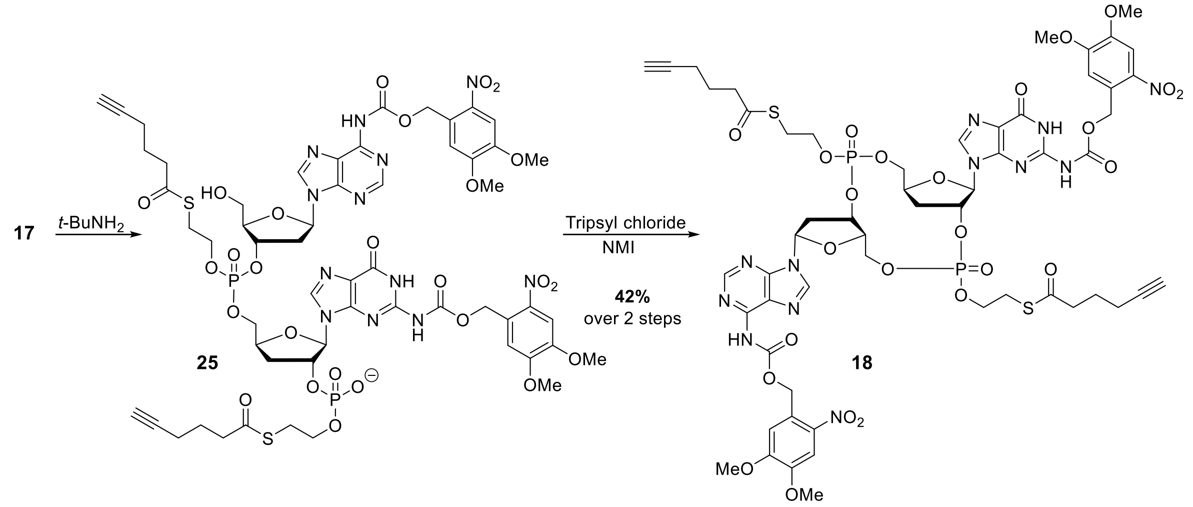


To a solution of **17** (175 mg, 117 µmol) in 14 mL of $CH_3CN$ *t*-$BuNH_2$ (3 mL) was added at 0 °C and the solution stirred at 0 °C for 20 min. Toluene (15 mL) was added and the solvents were removed *in vacuo*. The residue was co-evaporated with toluene (2 x 25 mL) and purified by flash column chromatography using a gradient elution (MeOH:$CH_2Cl_2$, 5:95, 15:85, 20:80) to yield **25** (120 mg, 83 µmol, 71%) as a yellow solid.

To a solution of **25** (28 mg, 19 µmol) in THF (3.9 mL) was added 3 Å molecular sieves, followed by addition of 2,4,6-triisopropylbenzenesulfonyl chloride (tripsyl chloride) (89 mg, 295 µmol) and *N*-methylimidazole (NMI) (24 uL, 292 µmol). The solution was stirred at rt for 2 d. The product was extracted with EtOAc (3 x 15 mL), the combined organic phases washed with satd. aq. $NaHCO_3$ (3 x 20 mL) and brine (20 mL), dried over $Na_2SO_4$ and concentrated *in vacuo*. The residue was purified by flash column chromatography (MeOH:$CH_2Cl_2$, 1:99 to 5:95) to yield the cyclic dinucleotide **18** (16 mg, 11 umol, 58%) as a mixture of four diastereomers. The residue was further purified by C18-HPLC using the following gradient: Solvent A, 0.1% TFA in $H_2O$; solvent B, 0.1% TFA in $CH_3CN$; 0-30 min gradient 40-100% B, 30-45 min isocratic 100% B, 45-50 min 100-40% B. One pure isomer was used for the characterization by NMR and MS. $R_f$=0.40 (MeOH:$CH_2Cl_2$ 5:95); $^1$H NMR (500 MHz, $CD_2Cl_2$, ppm) δ=11.55 (s, 1H), 11.35 (s, 1H), 8.65 (s, 1H), 8.44 (s, 1H), 8.18 (s, 1H), 7.68 (s, 1H), 7.55 (s, 1H), 7.27 (s, 1H), 7.06 (s, 1H), 6.59 (t, *J* = 6.5 Hz, 1H), 5.95 – 5.91 (m, 1H), 5.80 (d, *J* = 7.4 Hz, 1H), 5.66 (m, 2H), 5.50 – 5.30 (m, *J* = 6.9 Hz, 3H), 4.75 – 4.68 (m, *J* = 17.6, 9.1, 3.8 Hz, 2H), 4.49 – 4.45

(m, 2H), 4.27 – 4.22 (m, 3H), 4.15 – 4.10 (m, 3H), 3.96 (s, 3H), 3.94 (s, 3H), 3.91 (s, 3H), 3.88 (s, 3H), 3.36 (dt, *J* = 13.3, 6.1 Hz, 1H), 3.25 (td, *J* = 6.9, 3.7 Hz, 2H), 3.14 (t, *J* = 6.6 Hz, 2H), 3.04 – 3.01 (m, 1H), 2.76 (t, *J* = 7.4 Hz, 2H), 2.70 – 2.65 (m, 3H), 2.62 – 2.56 (m, 1H), 2.24 (dtd, *J* = 22.5, 7.0, 2.7 Hz, 4H), 2.03 (dt, *J* = 16.2, 2.6 Hz, 2H), 1.85 (dp, *J* = 23.4, 7.1 Hz, 4H); $^{13}$C NMR (126 MHz, $CD_2Cl_2$, ppm) δ=198.30, 198.16, 155.04, 154.62, 154.59, 154.09, 151.81, 151.28, 149.26, 148.88, 148.71, 140.90, 140.30, 139.70, 126.65, 125.09, 112.37, 110.59, 108.68, 108.47, 89.06, 86.19, 83.33, 83.27, 79.84, 78.85, 78.35, 75.48, 74.28, 70.14, 69.70, 69.59, 67.79, 67.75, 67.63, 67.58, 66.02, 65.85, 65.68, 57.07, 56.89, 56.73, 56.69, 42.96, 42.89, 28.93, 28.90, 28.88, 28.85, 24.46, 24.43, 17.97, 17.92; $^{31}$P NMR (202 MHz, $CD_2Cl_2$, ppm) δ=1.08, -0.00; HRMS (ESI): *m/z* calcd for $C_{56}H_{62}N_{12}O_{25}P_2S_2$+$H^+$: 1429.2938 $[M+H]^+$; found: 1429.2991.

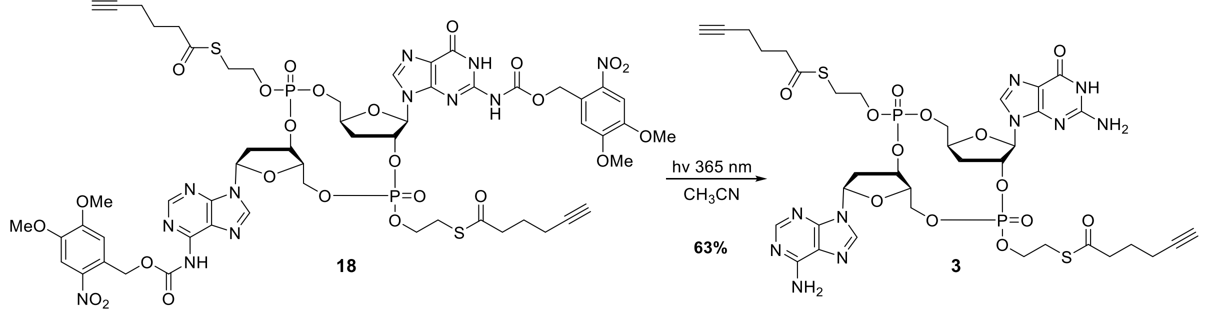


A solution of **18** (2.9 mg, 0.0020 mmol) in $CH_3CN$ (2 mL) was irradiated at 365 nm for 12 min. The solution was diluted with $CH_2Cl_2$ (20 mL), the organic phase washed with satd. aq. $NaHCO_3$ (3 x 20 mL), dried over $Na_2SO_4$ and concentrated *in vacuo*. The residue was purified by C18-HPLC using the following gradient: Solvent A, 0.1% TFA in $H_2O$; solvent B, 0.1% TFA in $CH_3CN$; 0-35 min gradient 20-50% B, 35-40 min gradient 50-100% B, 40-45 min isocratic 100% B, 45-50 min 100-20% B. **3** (1.2 mg, 0.0013 mmol, 63%) was obtained as a white powder. One pure isomer was used for the characterization by $^{1}$H, $^{31}$P, COSY HSQS and HMBC NMR and MS. $R_f$=0.50 (MeOH:$CH_2Cl_2$ 10:90); $^{1}$H NMR (800 MHz, DMSO-$d_6$, ppm) δ=10.70 (s, 1H), 8.35 (s, 1H), 8.15 (s, 1H), 7.85 (s, 1H), 7.33 (s, 2H), 6.53 (s, 2H), 6.45 – 6.40 (m, 1H), 5.89 (d, *J* = 4.8 Hz, 1H), 5.30 (s, 1H), 5.19 – 5.15 (m, 1H), 4.48 (s, 1H), 4.47 – 4.38 (m, 3H), 4.22 (dt, *J* = 10.8, 5.1 Hz, 1H), 4.19 – 4.17 (m, 2H), 4.12 – 4.07 (m, 3H), 3.41 (dd, *J* = 14.4, 8.1 Hz, 1H), 3.24 – 3.20 (m, 2H), 3.19 – 3.14 (m, 2H), 2.78 (dt, *J* = 13.2, 2.5 Hz, 2H), 2.75 – 2.71 (m, 1H), 2.70 – 2.65 (m, 5H), 2.57 – 2.55 (m, 1H), 2.14 (qd, *J* = 7.1, 2.6 Hz, 4H), 1.71 – 1.67 (m, 4H); $^{13}$C NMR (126 MHz, DMSO-$d_6$, ppm) δ=198.22, 181.69, 157.61, 156.02, 154.74, 153.19, 129.99, 127.76, 117.01, 116.25, 99.85, 83.40, 72.33, 42.14, 42.10, 33.65, 31.28, 29.00, 28.88, 28.69, 28.35, 27.92, 24.45, 23.86, 23.79, 22.08, 16.90, 13.94; $^{31}$P NMR (162 MHz, DMSO-$d_6$, ppm) δ=-3.32, -3.52; HRMS (ESI): *m/z* calcd for $C_{36}H_{44}N_{10}O_{13}P_2S_2$+$H^+$: 951.2079 $[M+H]^+$; found: 951.2107.

# 4 Cell culture and biological assays

## Cell culture

THP1 monocytic cells (male) were purchased from Cell Lines Service (CLS, catalog number 300356). They were cultured according to the manufacturer's instructions using RPMI-1640 (Sigma-Aldrich, R0883) supplemented with 10% (*v*/*v*) FBS (Gibco, 10500-064), 2 mM Alanyl-glutamine (Sigma-Aldrich, G8541), and 1 mM Sodium pyruvate solution (Sigma-Aldrich, S8636). The cells were kept between $0.1x10^6$/mL and $1x10^6$/mL either by addition of fresh medium or complete medium replacement every 2 to 3 d.

THP1-Dual™ (InvivoGen, Cat. Code thpd-nfis) and THP1-Dual™ KO-STING (InvivoGen, Cat. Code thpd-kostg) cells carrying two inducible reporter constructs were purchased from InvivoGen and cultured according to the manufacturer's instructions. Initial cultures were kept in RPMI-1640 (Sigma-Aldrich, R0883) supplemented with 20% (*v*/*v*) FBS (Gibco, 10500-064), 2 mM Alanyl-glutamine (Sigma-Aldrich, G8541), 25 mM HEPES (Sigma-Aldrich, 0887), 100 µg/mL Normocin™ (InvivoGen) and 1% Penicillin-Streptomycin (Sigma-Aldrich, P0781) before reducing the amount of FBS to 10% (*v*/*v*). After 2 to 3 passages, Blasticidin (InvivoGen) and Zeocin® (InvivoGen) were added to the medium upon each passage of the cells. The medium was replaced every 2 to 3 days to maintain a cell density between $0.5x10^6$/mL and $2x10^6$/mL.

## *In vitro* CES1 cleavage assay

The cleavage assay to study the removal of the HTE protecting groups with CES1 was performed in a buffer (20 mM HEPES, 150 mM NaCl, pH 7.5) containing **3** (10 nmol, 0.1 nmol/µL) and human carboxylesterase 1 (CES1) isoform C (5 units, Sigma-Aldrich) at 37 °C under shaking (600 rpm). Simultaneously, an assay in absence of the enzyme was performed. Aliquots of the reaction-solution (10 µL = 1 nmol **3**) were analyzed by RP-HPLC at different time-points: 0, 1, 4 and 24 h (Figure S1A). The reaction mixture was centrifuged and mixed with a pipet before each sample collection. First, one HTE protecting group of **3** is removed, giving an intermediate **i** and later the second protecting group is cleaved yielding in dd-cGAMP **2** (Figure S1A). To test the stability of **3** in the buffer used for the cleavage assay, we performed the same experiment without the CES1 enzyme (Figure S1B). **3** is stable in the buffer system for at least 4 h. After 24 h, most of **3** is still intact, but we could see a small peak for the intermediate **i** and for the fully cleaved compound **2**. This shows that **3** is partially cleaved in the buffer but very slowly and that CES1 is the main component in the cleavage assay to cleave the HTE groups of **3**. The HPLC analyses were performed on a RP-C18 column using the following gradient: Solvent A, 2 mM $NH_4HCOO$ in $ddH_2O$; solvent B, 2 mM $NH_4HCOO$ in 80% $CH_3CN$ in $ddH_2O$; 0-10 min gradient 0-100% B, flow rate of 1 mL/min.

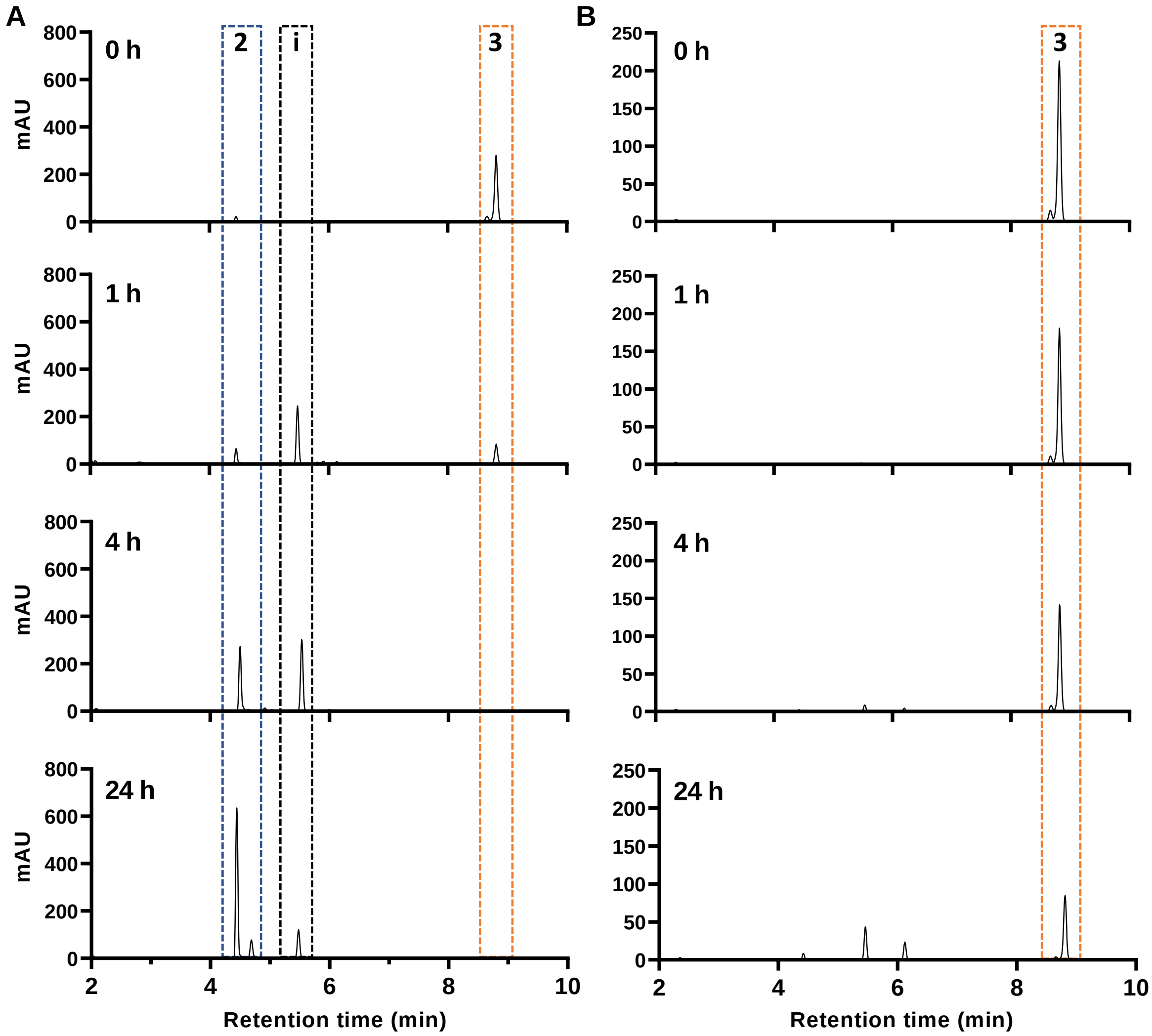


**Figure S1**. A) HPLC chromatograms of the CES1 cleavage assay and monitored at 0, 1, 4 and 24 h incubation times. First, one HTE group of **3** is removed to give an intermediate **i**. Then the second protecting group is cleaved yielding in dd-cGAMP **2**. B) HPLC chromatograms of a control experiment of **3** in the buffer that was used for the CES1 cleavage assay without the CES1 enzyme. The control was monitored at 0, 1, 4 and 24 h incubation times.

The elution peaks at 8.7 min, 5.5 min and 4.5 min in Figure S1A from the *in vitro* CES1 cleavage assay were collected and analyzed by LC-MS (QExactive Orbitrap, ThermoFischer) (Figure S2A). The identified masses (Figure S2B), single and double charged, corresponded to the exact masses of bis-HTE-dd-cGAMP **3**, intermediate **i** where one HTE group has been removed and fully deprotected dd-cGAMP **2**, respectively.

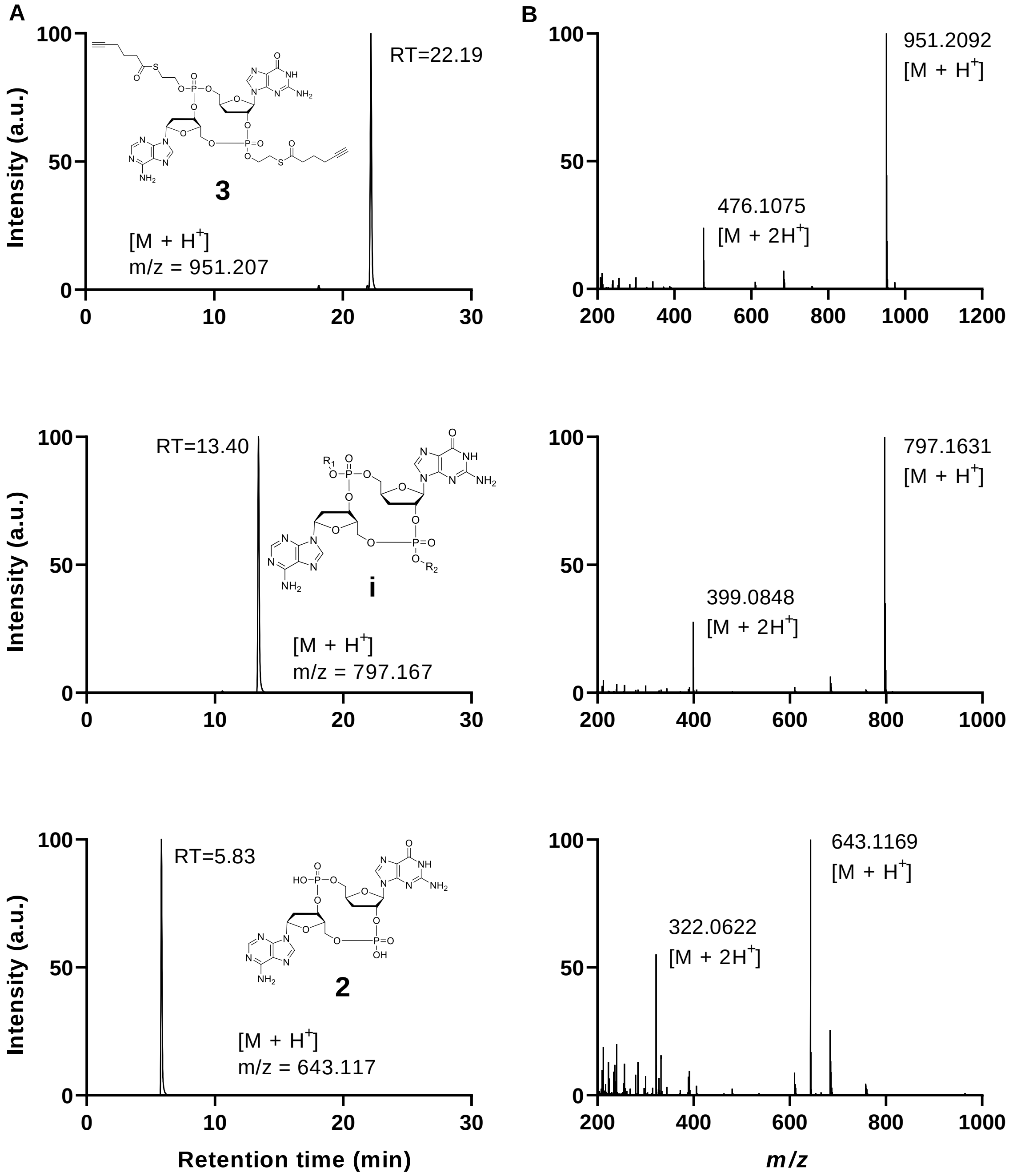


**Figure S2**. A) Extracted ion chromatograms and B) mass spectra (right column) of bis-HTE-dd-cGAMP **3**, HTE-dd-cGAMP with only one HTE protecting group (**i**) and dd-cGAMP **2**. $R_1$ = H or HTE protecting group, $R_2$ = H or HTE protecting group.

## *In cellulo* HTE cleavage

THP1 cells ($5x10^6$, $0.5x10^6$/mL) were treated with bis-HTE-dd-cGAMP **3** (1 µM) and incubated at 37°C for 30 min and 4 h (Figure 2B). The cells were harvested by 5 min centrifugation at 100 x g. Then, the cell pellets were washed with DPBS (900 µL) and centrifuged 3 min at 200 x g. The pellets were dissolved in ice-cold $ddH_2O$:$CH_3CN$ (1:1, 1000 µL) and incubated on ice for 10 min. During the incubation, the samples were vortexed 3 times. After incubation, the cells were centrifuged for 10 min at 10000 x g at 4°C. The supernatant containing the

metabolites was divided into two halves and transferred into two new Eppendorf tubes (0.5 mL in each) and subsequently lyophilized overnight. After lyophilization one half was dissolved in $ddH_2O$ (50 µL) and the other in $ddH_2O$:$CH_3CN$ (1:1, 50 µL). The dissolved metabolites were filtered through AcroPrep Advence 96 Well plates with 0.2 µm pore size (Pall Corporation) at 3220 x g, 4°C. Subsequently, 20 µL were injected and analyzed on LC-MS system (Q Exactive HF Orbitrap, Thermo Scientific). The HPLC analysis was performed on a Uptisphere C18-HDO 3 µm 150 x 2.1mm HPLC column using the following gradient: Solvent A, 2 mM $NH_4HCOO$ in $ddH_2O$, pH 5.5; solvent B, 2 mM $NH_4HCOO$ in 80% $CH_3CN$ in $ddH_2O$, pH 5.5; 0-25 min gradient 0-70% B, 25-26 min gradient 70-100% B, 26-31 min isocratic 100% B, flow rate of 0.2 mL/min, analyzed at 260 nm. The ions were scanned in positive polarity mode over a scan range of *m/z* = 100 - 1000. Bis-HTE-dd-cGAMP **3** was detected at the range *m/z* = 951.1981 – 951.2171, in a single charged state $[M+H]^+$, with the exact mass of *m/z* = 951.207 (found: 951.2092). Deprotected dd-cGAMP **2** was detected at the range *m/z* = 643.1106 – 643.1234, in a single charged state $[M+H]^+$, with the exact mass of *m/z* = 643.117 (found: 643.1169). Data were analyzed using Xcalibur from Thermo Scientific.

To analyze the stability of bis-HTE-dd-cGAMP **3** in THP1 cell medium (with 10% FBS), **3** (12 nmol) was incubated in the medium at 37 °C under shaking (Figure S3). Analyses were performed by HPLC of the cell medium without **3** (top chromatogram Figure S3) and at different time-points (1.2 nmol of **3**); 0, 1, 4, 24h. bis-HTE-dd-cGAMP **3** elutes at 30.2 min (Figure S3). The chromatogram of only the cell-medium without **3** has UV active peaks which is not surprising as the cell medium is a complex mixture of many components. We could see that compound **3** is completely stable up to 4 h in the cell medium and after 24 h we could still see at least 50% of compound **3** fully intact. The HPLC analyses were performed on a RP-C18 column using the following gradient: Solvent A, 2 mM $NH_4HCOO$ in $ddH_2O$; solvent B, 2 mM $NH_4HCOO$ in 80% $CH_3CN$ in $ddH_2O$; 0-45 min gradient 0-100% B, flow rate of 1 mL/min.

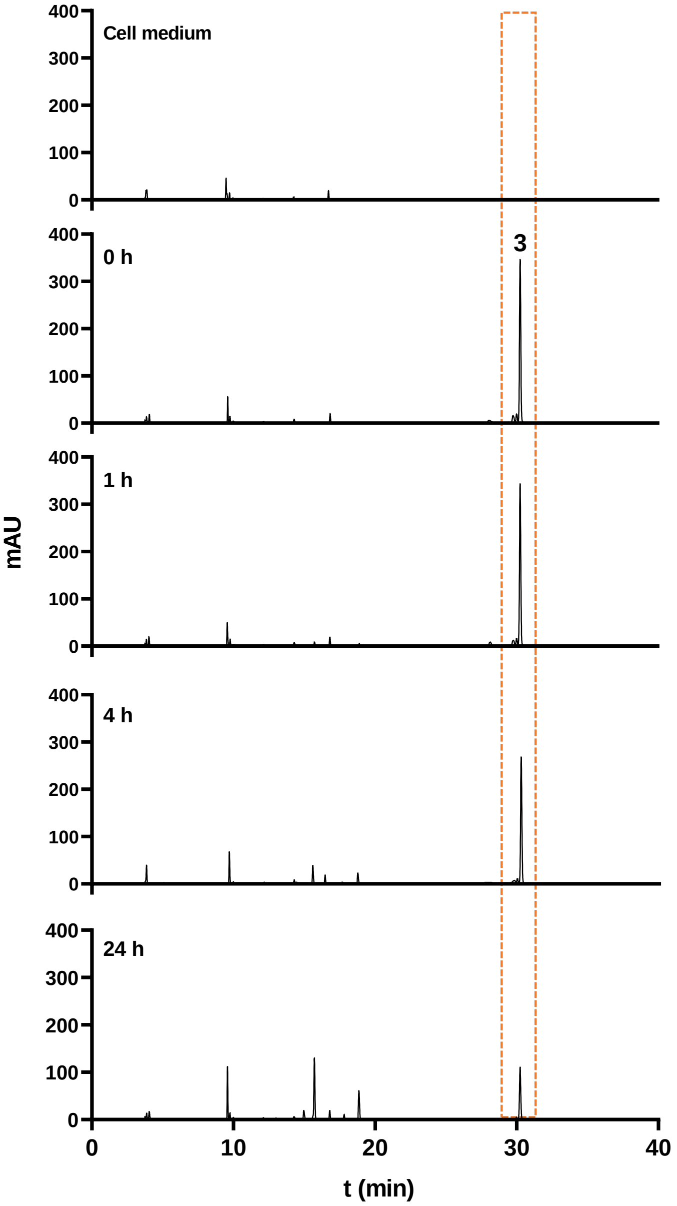


**Figure S3**. HPLC chromatograms of bis-HTE-dd-cGAMP **3** in THP1 cell medium containing 10% (*v*/*v*) FBS (Gibco, 10500-064), monitored without **3** (top chromatogram) and at 0, 1, 4 and 24 h. Bis-HTE-dd-cGAMP **3** was detected at 260 nm with an elution time of 30.2 min.

## IFN Induction Assay

The ability of the compounds to induce STING-mediated IFN signaling was assessed by the luciferase reporter system provided by the THP1-Dual™ cells. Different concentrations of the compounds ranging from 0.01 nM to 100 µM were prepared in flat-bottom 96-well plates (Sarstedt) before adding 0.1x10$^6$/mL cells/well in medium without the selective antibiotics Blasticidin, Zeocin® or Normocin™, leading to a final volume of 200 µL/well. After incubation at 37 °C for 24 h and 5% $CO_2$, 20 µL of the culture medium was transferred to a white (opaque) 96-well plate (Thermo Scientific). Luciferase activity was determined in technical duplicates as end-point reading after automatic injection of 50 µL of QUANTI-LUC™ assay solution

(InvivoGen) per well, 2 sec shaking, and a further 2 sec delay using Cytation5 (Agilent). The data were analyzed using GraphPad Prism 9.5.0.

To confirm that the IFN signaling is fully dependent on the presence of STING, THP1-Dual™ KO-STING cells were treated with bis-HTE-dd-cGAMP **3** in the same concentration range as in THP1-Dual™ cells and luciferase activity was determined (Figure S4). As expected, no signal was detected in these knockout cells, indicating that IFN production could not be triggered in the absence of STING.

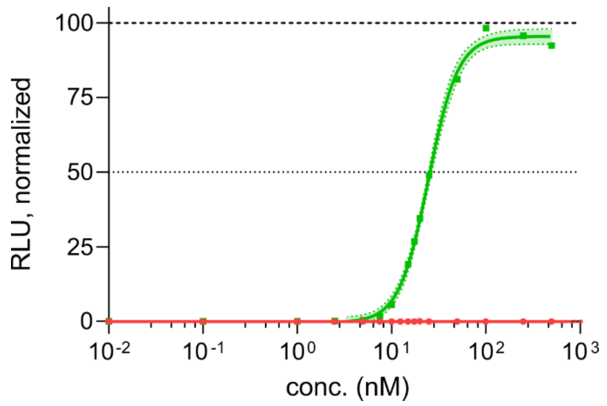


**Figure S4**. Dose-dependent response of both THP1-Dual™ (green rectangles) and THP1-Dual™ KO-STING (red circles) cells to bis-HTE-dd-cGAMP **3**. Individual items represent the mean of at least three biologically independent experiments, shade represents the 95% CI.

## Proteomics

### Sample preparation

For proteomic analysis, 0.6 Mio THP1 cells per mL were treated with 40 nM bis-HTE-dd-cGAMP **3** or left untreated for 18 h in 4 replicates each. After the treatment, cells were harvested at 150 rcf for 4 min and washed with 500 µL cold DPBS (Sigma, D8537) before lysis in RIPA buffer containing 1x cOmplete™ protease inhibitor cocktail (Roche) under gentle agitation for 15 min at 4 °C. Cell debris was removed by centrifugation at 14,000 rcf for 15 min and protein concentration was determined via Bradford analysis. To 20 µg of total proteins 20 µL of a 1:1 mixture of hydrophilic and hydrophobic carboxylate-coated magnetic beads (Cytiva, 65152105050250), 3x pre-washed with 100 µL MS-grade water, were added and mixed in a Thermoshaker (Eppendorf, ThermoMixer C) for 1 min at 850 rpm. 60 µL of absolute ethanol was added and mixed again for 1 min at 850 rpm. The beads were then washed 3x with 100 µL 80% ethanol, each time under mixing for 1 min at 850 rpm. After the last wash, 60 µL of 100 mM ABC buffer and 0.5 µg/µL trypsin (Promega, V5111) were added and the mixture was incubated overnight at 37 °C while shaking at 850 rpm. The peptide mixture was transferred to a fresh tube and the beads were washed with 50 µL and 30 µL 1% formaldehyde

while incubating at 40 °C and 850 rpm for 5 min. 200-300 ng were directly analyzed via LC-MS/MS.

**LC-MS/MS measurement**

MS measurements were carried out on an Orbitrap Eclipse Tribrid Mass Spectrometer (Thermo Fisher Scientific) connected to an UltiMate 3000 Nano-HPLC system (Thermo Fisher Scientific) via a Nanospray Flex and FAIMS interface (Thermo Fisher Scientific). Peptides were initially loaded onto an Acclaim PepMap 100 µ-precolumn cartridge (5 µm, 100 Å; 300 µm ID x 5 mm, Thermo Fisher Scientific) and subsequently separated at 40 °C on a PicoTip emitter (non-coated, 15 cm, 75 µm ID, 8 µm tip, New Objective) packed in-house with ReprosilPur 120 C18-AQ material (1.9 µm, 150 Å, Dr. A. Maisch GmbH). LC buffers included MS-grade water (Buffer A) and acetonitrile (Buffer B), both containing 0.1% formic acid. The separation gradient ranged from 4% to 35.2% Buffer B over a 60-minute run (0–5 min at 4% B, 5–6 min to 7%, 7–36 min to 24.8%, 37–41 min to 35.2%, 42–46 min at 80%, and 47–60 min back to 4%) at a flow rate of 300 nL/min.

For data-independent acquisition (DIA), FAIMS was conducted with one compensation voltage (CV) of -45 V. One DIA cycle included one MS1 scan followed by 30 MS2 scans. The mass spectrometer was operated in DIA mode with the following parameters: polarity set to positive, MS1 Orbitrap resolution at 60k, standard MS1 AGC target, and a maximum MS1 injection time of 50 ms. The MS1 scan range was set from m/z 200–1800, with an RF lens at 30%. The precursor mass range was m/z 500–740 with an isolation window of m/z 4 and a window overlap of m/z 2. MS2 Orbitrap resolution was 30k, MS2 AGC target 200%, automatic maximum MS2 injection time, and HCD collision energy at 35%, while the RF lens was maintained at 30%. The MS2 scan range was set to automatic.

This method is analog to the one previously reported.[5]

**Data analysis**

The resulting *.raw files were converted to *.mzML files using ProteoWizard 3.0[6] using the standard settings and analyzed with DIA-NN 1.9.1[7] against a library previously generated from the Uniprot database for Homo sapiens (taxon identifier: 9606) with the following settings: protease: Trypsin/P, missed cleavages: 1, N-term M excision: enabled, C carbamidomethylation: enabled, Generate spectral library: enabled, Quantities matrices: enabled, Precursor FDR: 1%, scan window: 0, match between runs (MBR): enabled; residual settings were left with default settings.

The statistical analysis was performed in Perseus v2.1.3.0.[8] After categorical annotation of rows to define the two groups treated and control (ctrl), the quantified values were $\log_2$-transformed. The matrix was reduced via filtering the rows to a minimum number of 3 valid values out of 4 in at least one group before replacing missing values from normal distribution. The quality of the resulting reduced matrix was ensured by visualization as histograms with the imputations marked in red (Figure S5).

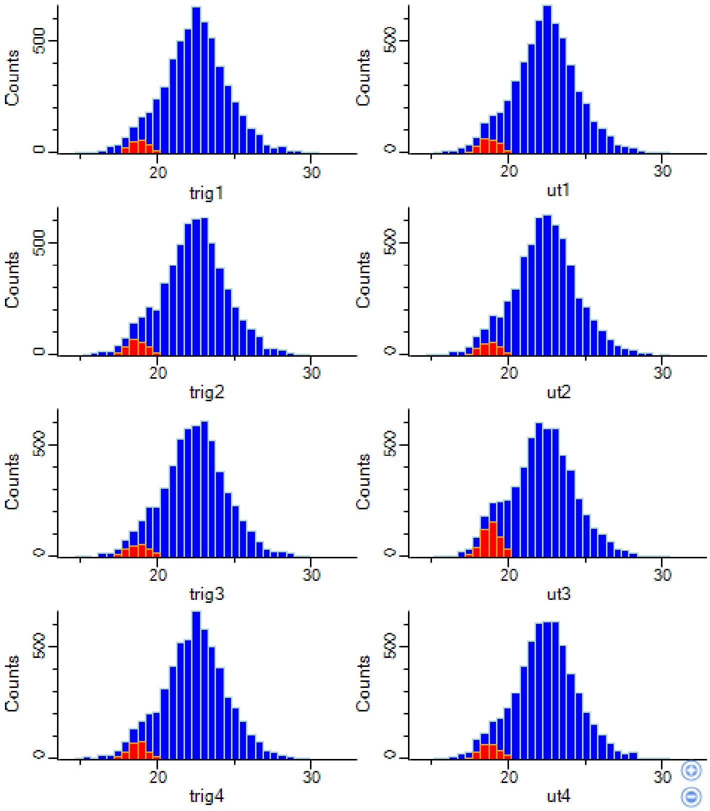


**Figure S5**. Histograms of the matrix that was further used to analyze proteomic samples. Imputation of missing values marked in red. The overall amount of imputations is low, thus ensuring high-quality samples.

After adding the main annotations for Homo sapiens, a two-sided Student's t-test of the two groups was conducted using the default settings and an FDR of 0.05 to obtain $-\log_{10}$($p$-values) using the volcano plot function. GraphPad 9.5.0 was used for visualization of the volcano plot.

To identify biological processes that were significantly upregulated by the treatment, a functional annotation clustering using the Database for Annotation, Visualization, and Integrated Discovery (DAVID)[9] was performed. For this, all proteins within the cut-off $p$-value 0.05 and fold-change 2 were uploaded as Gene list to DAVID. Homo sapiens was chosen as the species and a functional annotation clustering was performed with the following settings enabled: Functional Annotations: UP_KW_BIOLOGICAL_PROCESS, Gene Ontology:

GOTERM_BP_DIRECT. Three clusters with an enrichment score > 2 were identified and listed in Table S1.

**Table S1.** Three most enriched clusters (enrichment score > 2) identified by the functional annotation clustering tool DAVID.

| **Annotation Cluster 1** | **Enrichment Score: 27.44397922354581** | |
|---|---|---|
| **Category** | **Term** | **Genes** |
| **GOTERM_BP_DIRECT** | KW-0051 Antiviral defense | Q9BYX4, O15162, P52630, Q96C10, Q53G44, Q8WXG1, Q8IY21, Q9Y6K5, Q8IXQ6, P29728, Q96AZ6, P20592, Q01628, Q8TDB6, P09913, Q15646, O95786, P09914, Q9UII4, Q53F19, P20591, Q13309, O14879, Q9BYJ4, Q8IYM9, P05161, Q9P2E3, Q9NUL5, P00973, O14730, Q92985 |
| **GOTERM_BP_DIRECT** | GO:0051607 defense response to virus | Q9BYX4, O15162, P52630, Q53G44, Q8WXG1, Q16666, Q8IY21, Q9Y6K5, Q8IXQ6, P29728, Q96AZ6, P20592, Q01628, Q8TDB6, P09913, Q15646, O95786, P09914, Q9UII4, Q53F19, P20591, Q13309, O14879, Q9BYJ4, Q8IYM9, P05161, Q9P2E3, Q9NUL5, P00973, O14730, Q92985 |
| **UP_KW_BIOLOGICAL_PROCESS** | KW-0399 Innate immunity | Q9BYX4, Q96C10, Q8WXG1, Q16666, Q8IY21, Q9Y6K5, Q460N5, Q8IXQ6, Q96CV9, P02748, P29728, Q96AZ6, P20592, P07333, Q01628, Q8TDB6, P09913, Q15646, O95786, P09914, Q9UII4, P20591, Q13309, P30530, O14879, P05161, Q9P2E3, P00973, O14730, Q96PP9, Q92985 |
| **GOTERM_BP_DIRECT** | GO:0045087 innate immune response | Q9BYX4, Q96C10, Q8WXG1, O14791, Q16666, Q8IY21, Q9Y6K5, Q460N5, Q8IXQ6, Q96CV9, P29728, Q96AZ6, P20592, P07333, Q8TDB6, Q15646, O95786, Q9UII4, P20591, Q13309, P30530, P19878, Q9BYJ4, Q8IYM9, P05161, Q9P2E3, Q9NUL5, P00973, O14730, Q92985 |
| **GOTERM_BP_DIRECT** | GO:0009615 response to virus | Q9BYX4, P09913, Q15646, O95786, P09914, Q96C10, Q8WXG1, P20591, Q8IY21, Q9Y6K5, O14879, Q8IYM9, P05161, P29728, Q96AZ6, |

| | | |
|---|---|---|
| | | P20592, P00973, Q01628, Q92985 |
| **GOTERM_BP_DIRECT** | GO:0045071 negative regulation of viral genome replication | Q9BYX4, O15162, Q15646, P09914, Q16666, Q8WXG1, P20591, Q9Y6K5, P05161, Q9NUL5, Q9P2E3, P29728, Q96AZ6, P00973, Q01628 |
| **UP_KW_BIOLOGICAL_PROCESS** | KW-0391 Immunity | Q9BYX4, Q96C10, Q92637, Q8WXG1, Q16666, Q8IY21, Q9Y6K5, Q460N5, Q8IXQ6, Q96CV9, P02748, P29728, Q96RQ9, Q96AZ6, P20592, P07333, Q01628, Q8TDB6, P09913, Q15646, O95786, P09914, Q9UII4, P20591, Q13309, P30530, O14879, P05161, Q9P2E3, P00973, O14730, Q03518, Q96PP9, Q92985 |
| **Annotation Cluster 2** | **Enrichment Score: 5.394263098497642** | |
| **Category** | **Term** | **Genes** |
| **GOTERM_BP_DIRECT** | GO:0032728 positive regulation of interferon-beta production | Q9BYX4, O95786, P05161, P29728, Q9Y6K5, P00973, O14730, Q92985 |
| **GOTERM_BP_DIRECT** | GO:0060700 regulation of ribonuclease activity | Q15646, P29728, Q9Y6K5, P00973 |
| **GOTERM_BP_DIRECT** | GO:0060337 type I interferon-mediated signaling pathway | P52630, Q9BYX4, P29728, P00973, Q01628, Q92985 |
| **GOTERM_BP_DIRECT** | GO:0032760 positive regulation of tumor necrosis factor production | Q9BYX4, O95786, P29728, Q9Y6K5, P00973 |
| **GOTERM_BP_DIRECT** | GO:0042742 defense response to bacterium | P05161, Q9P2E3, P29728, Q9Y6K5, P00973 |
| **Annotation Cluster 3** | **Enrichment Score: 4.463701468920557** | |
| **Category** | **Term** | **Genes** |
| **GOTERM_BP_DIRECT** | GO:0032728 positive regulation of interferon-beta production | Q9BYX4, O95786, P05161, P29728, Q9Y6K5, P00973, O14730, Q92985 |
| **GOTERM_BP_DIRECT** | GO:0002753 cytoplasmic pattern recognition receptor signaling pathway | Q9BYX4, O95786, Q96C10, Q92985 |
| **GOTERM_BP_DIRECT** | GO:0032727 positive regulation of interferon-alpha production | Q9BYX4, O95786, Q92985 |
| **GOTERM_BP_DIRECT** | GO:0002376 immune system process | Q9BYX4, O95786, Q92985 |

# 5 NMR spectra of synthesized compounds

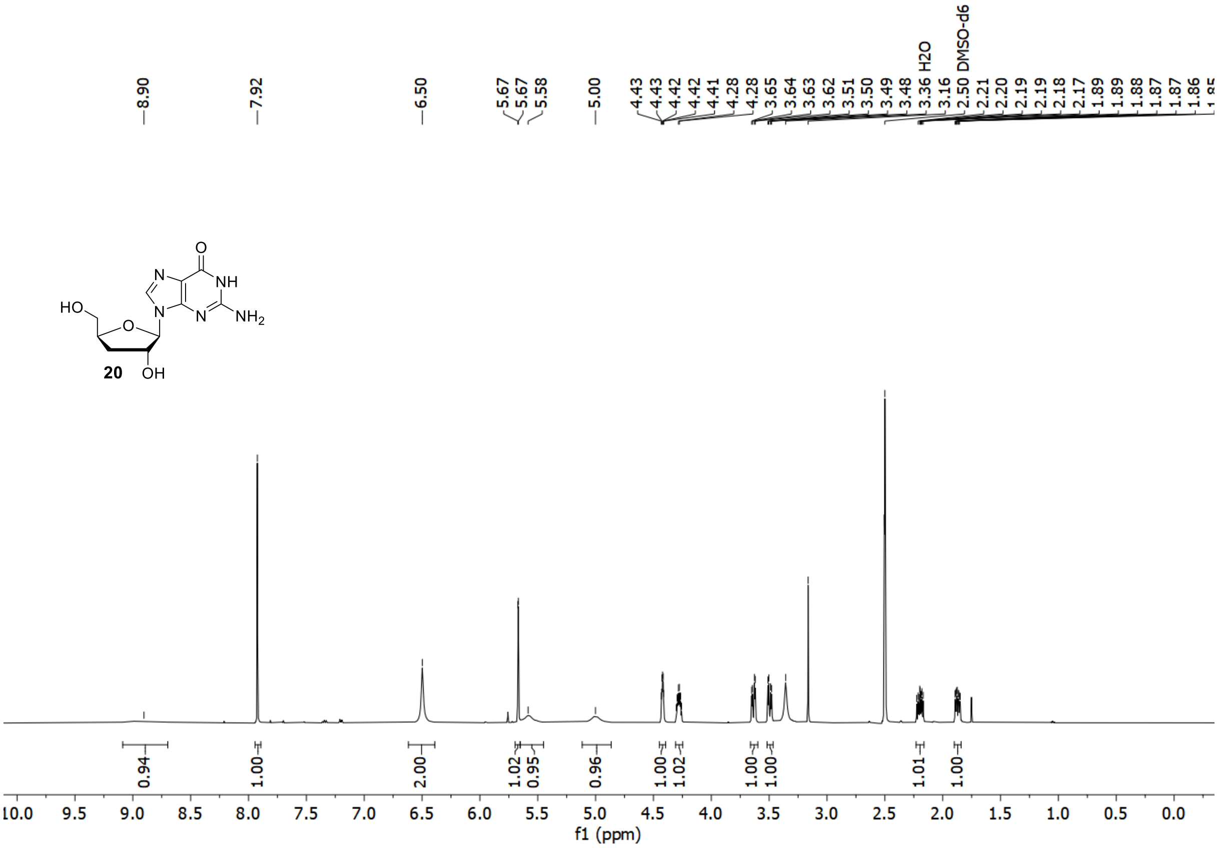


**Figure S6**. $^{1}$H NMR spectrum of **20**.

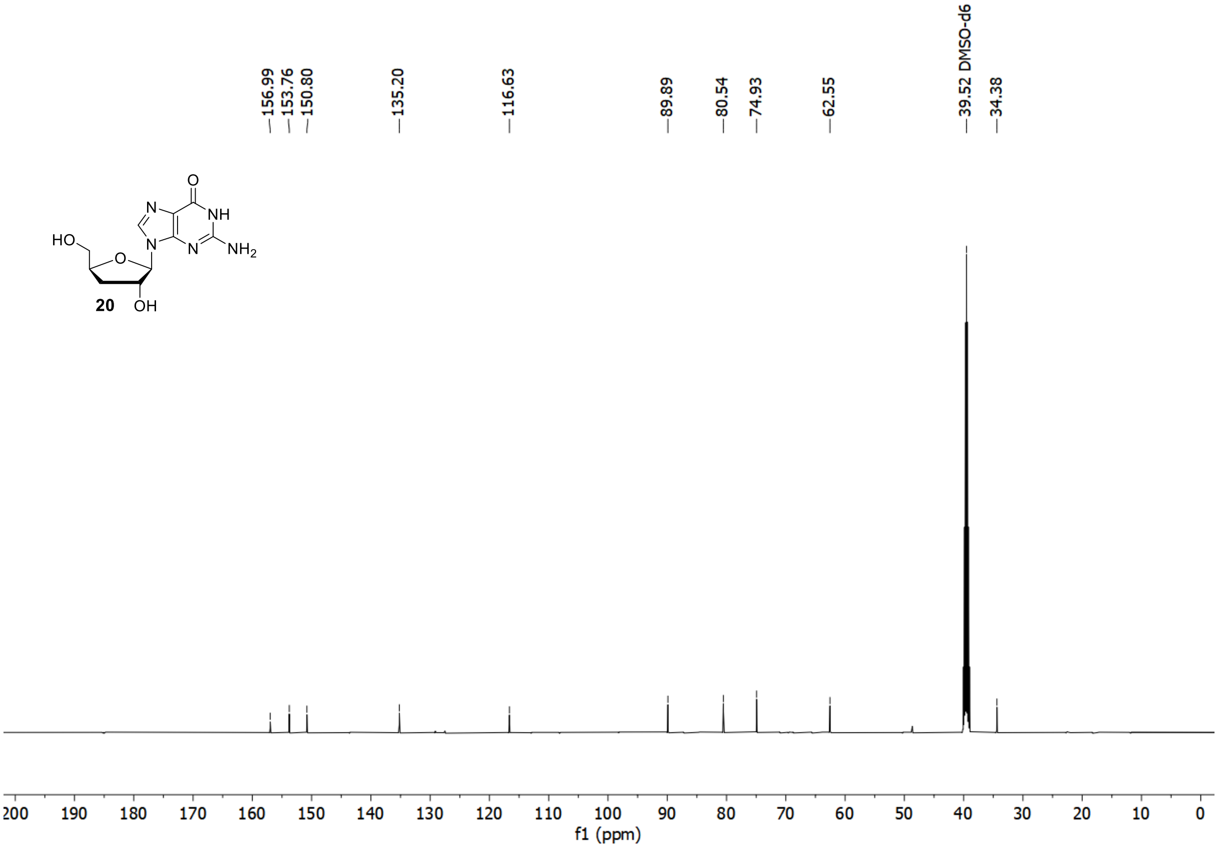


**Figure S7**. $^{13}$C{$^{1}$H} NMR spectrum of **20**.

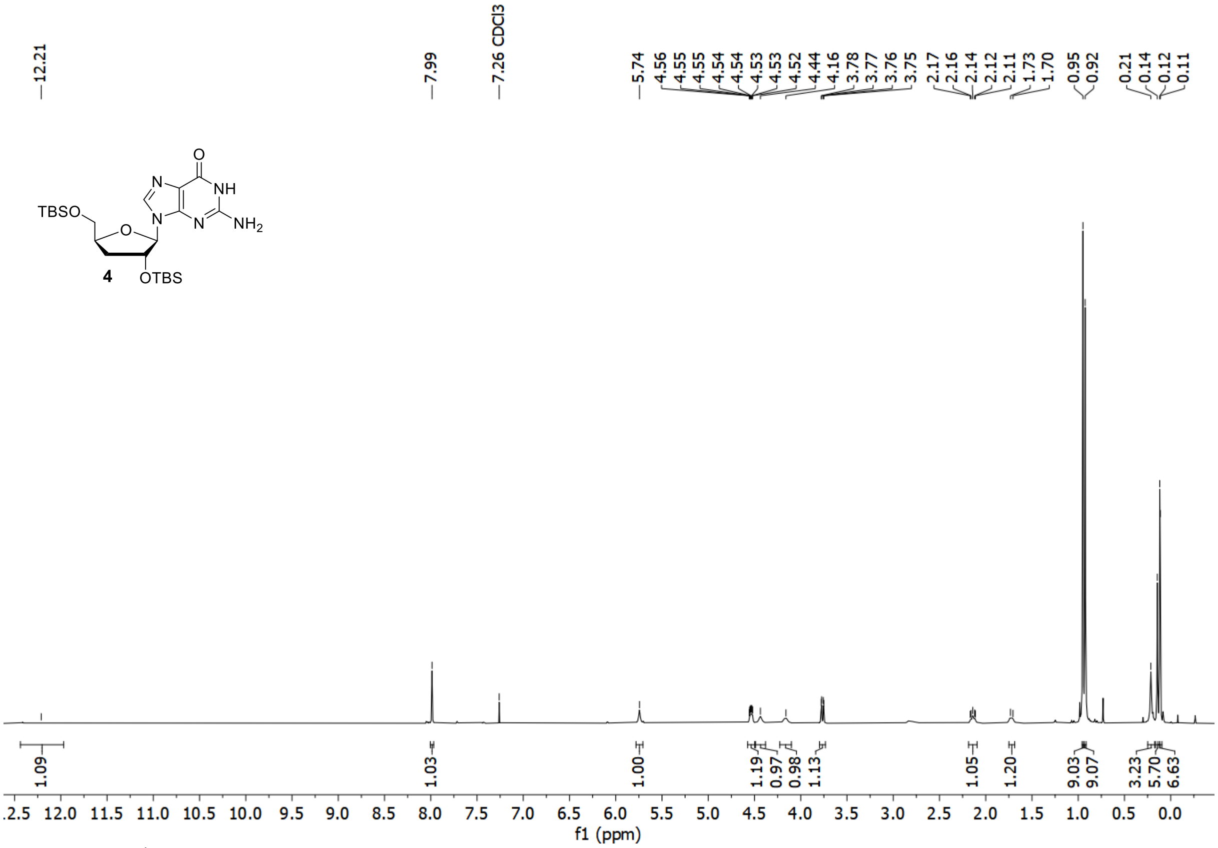


**Figure S8**. $^{1}H$ NMR spectrum of **4**.

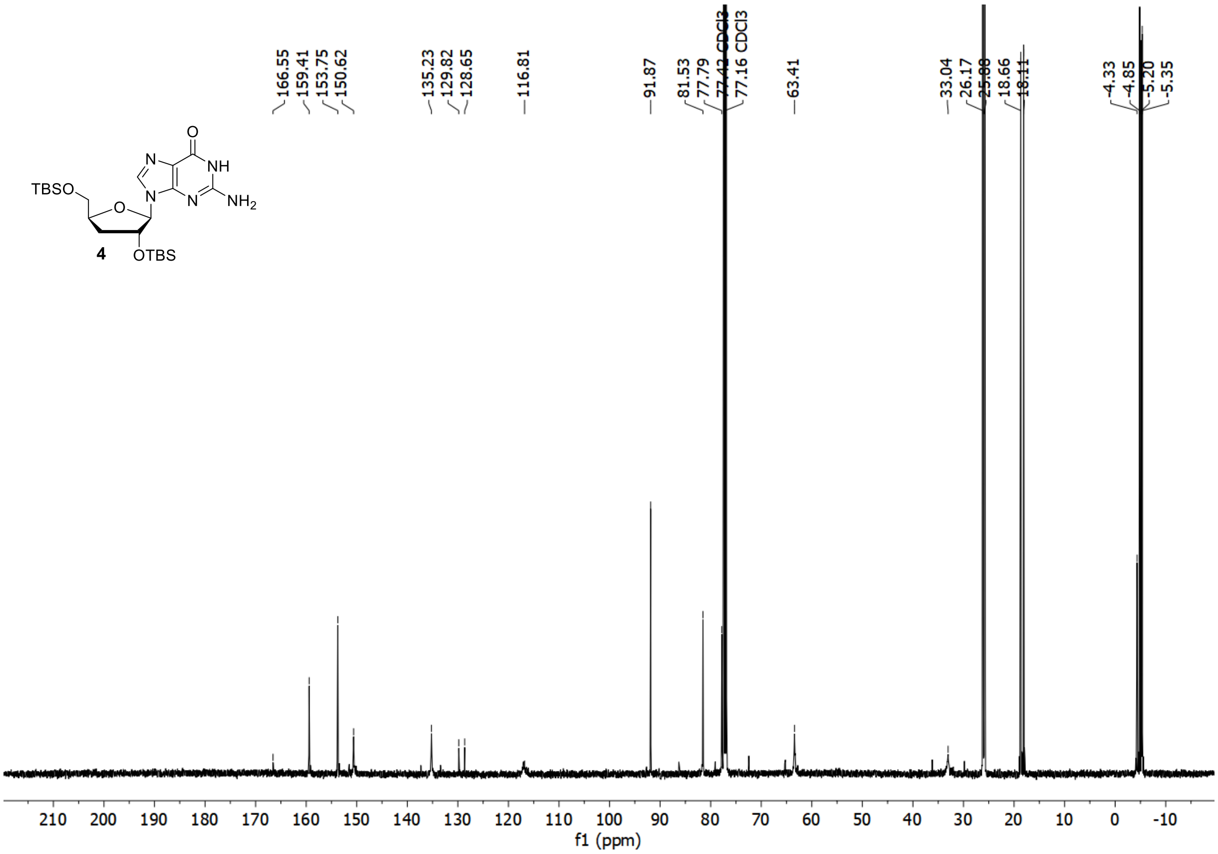


**Figure S9**. $^{13}C\{^{1}H\}$ NMR spectrum of **4**.

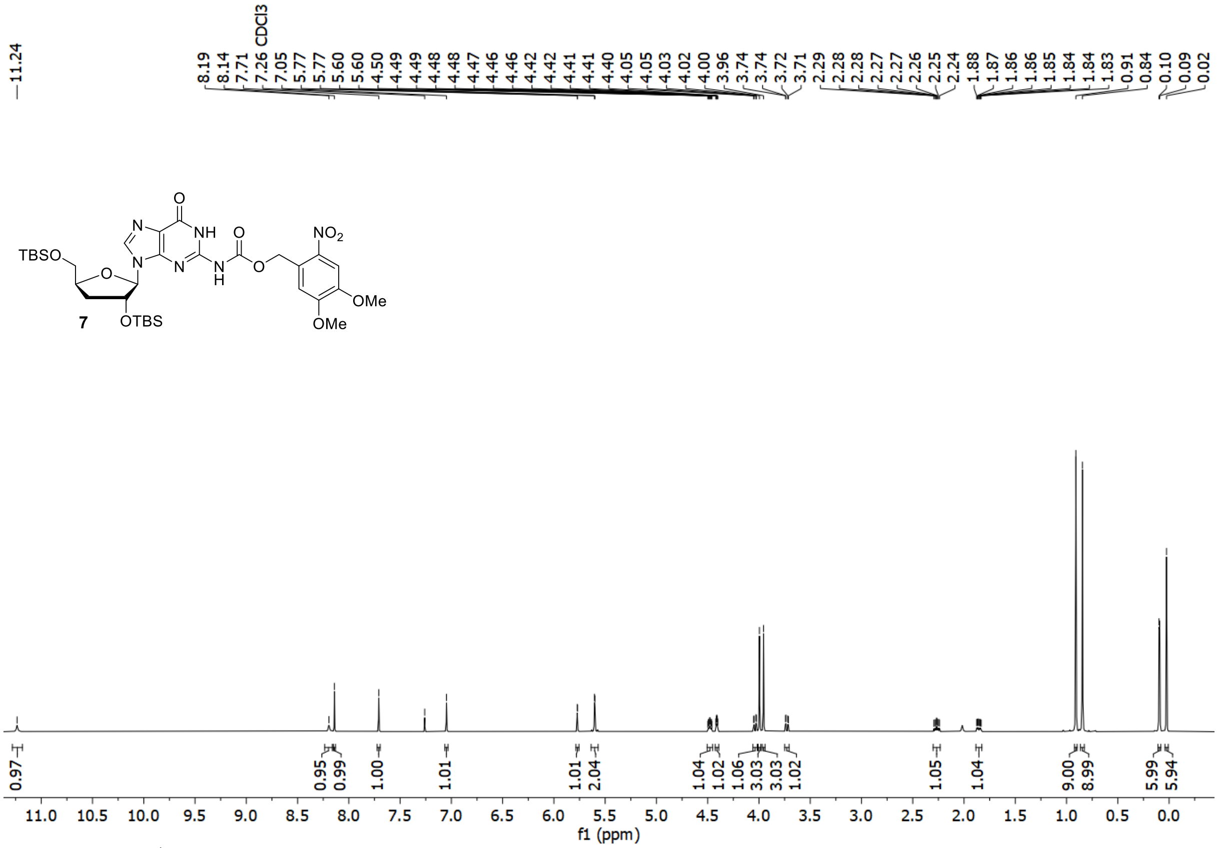

**Figure S10**. $^{1}$H NMR spectrum of **7**.

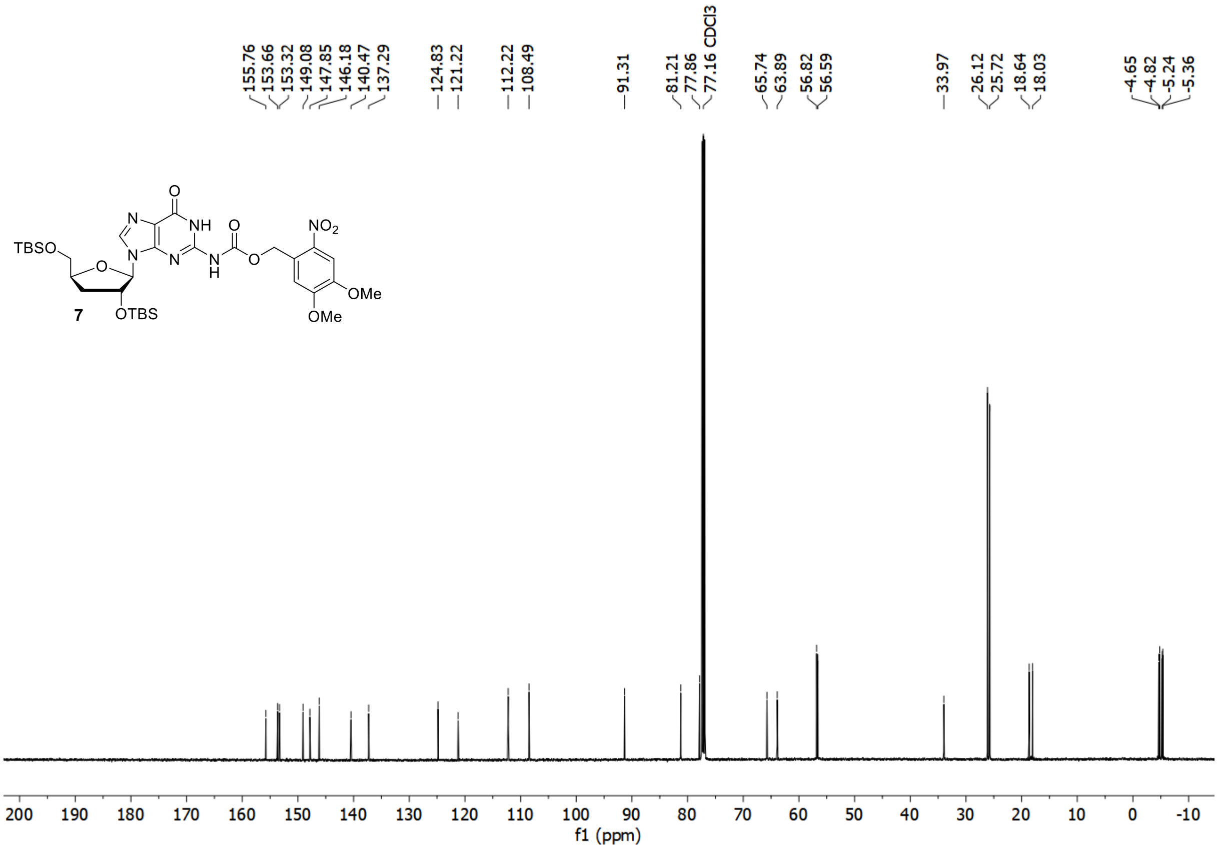

**Figure S11**. $^{13}$C{$^{1}$H} NMR spectrum of **7**.

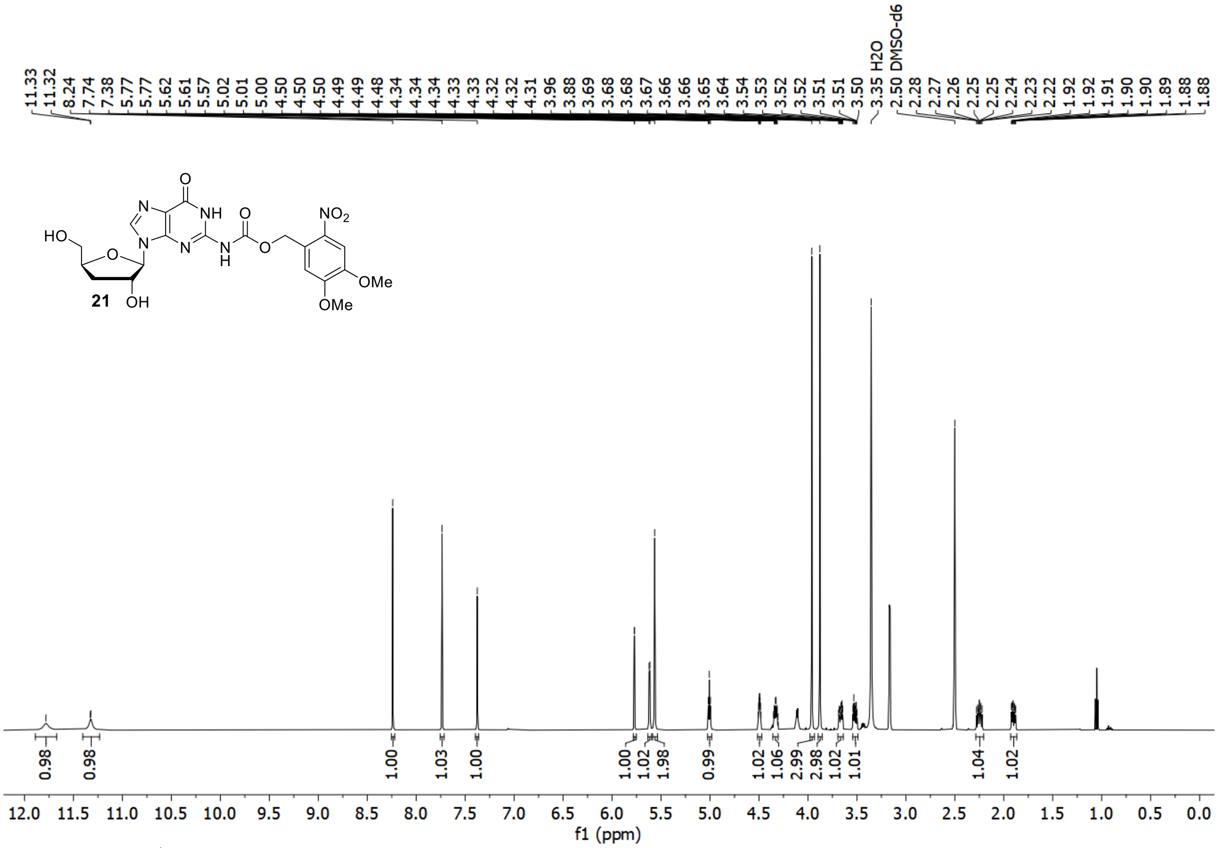


**Figure S12**. $^{1}$H NMR spectrum of **21**.

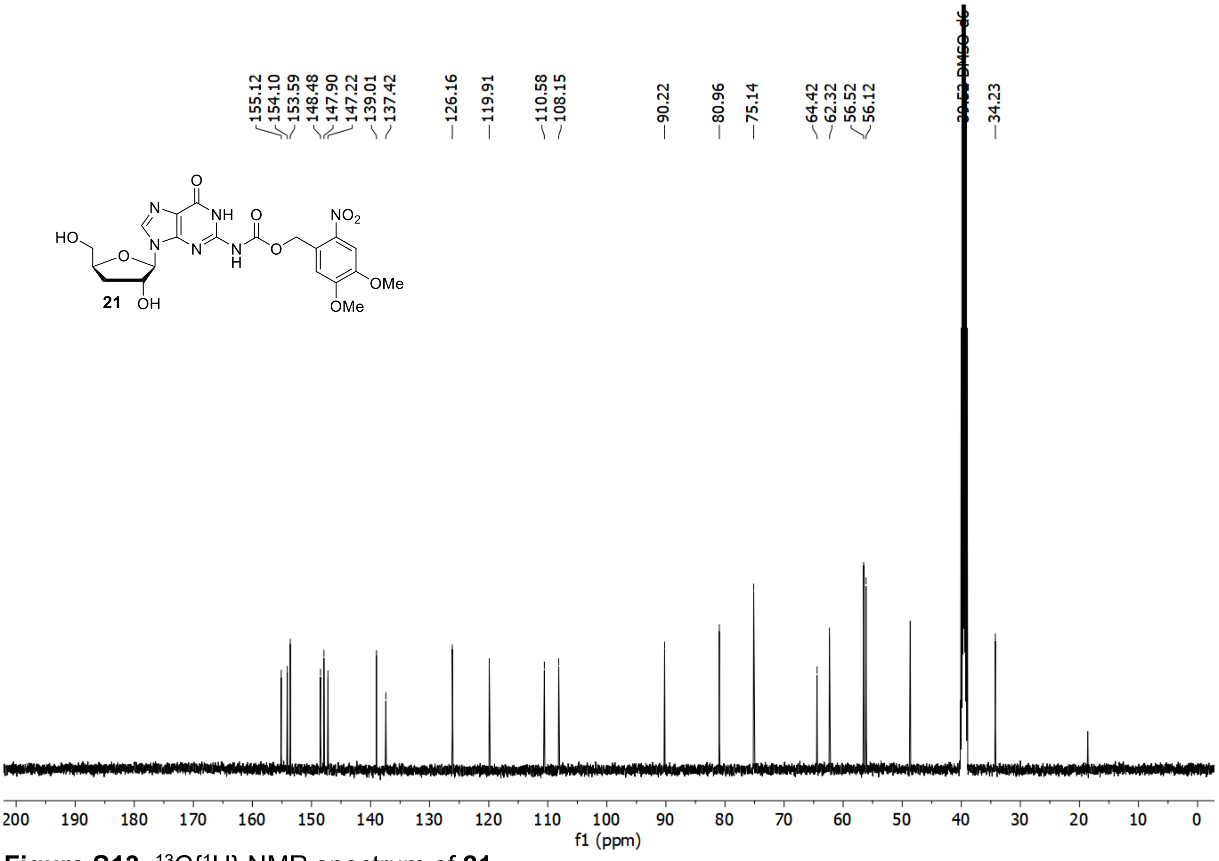


**Figure S13**. $^{13}$C{$^{1}$H} NMR spectrum of **21**.

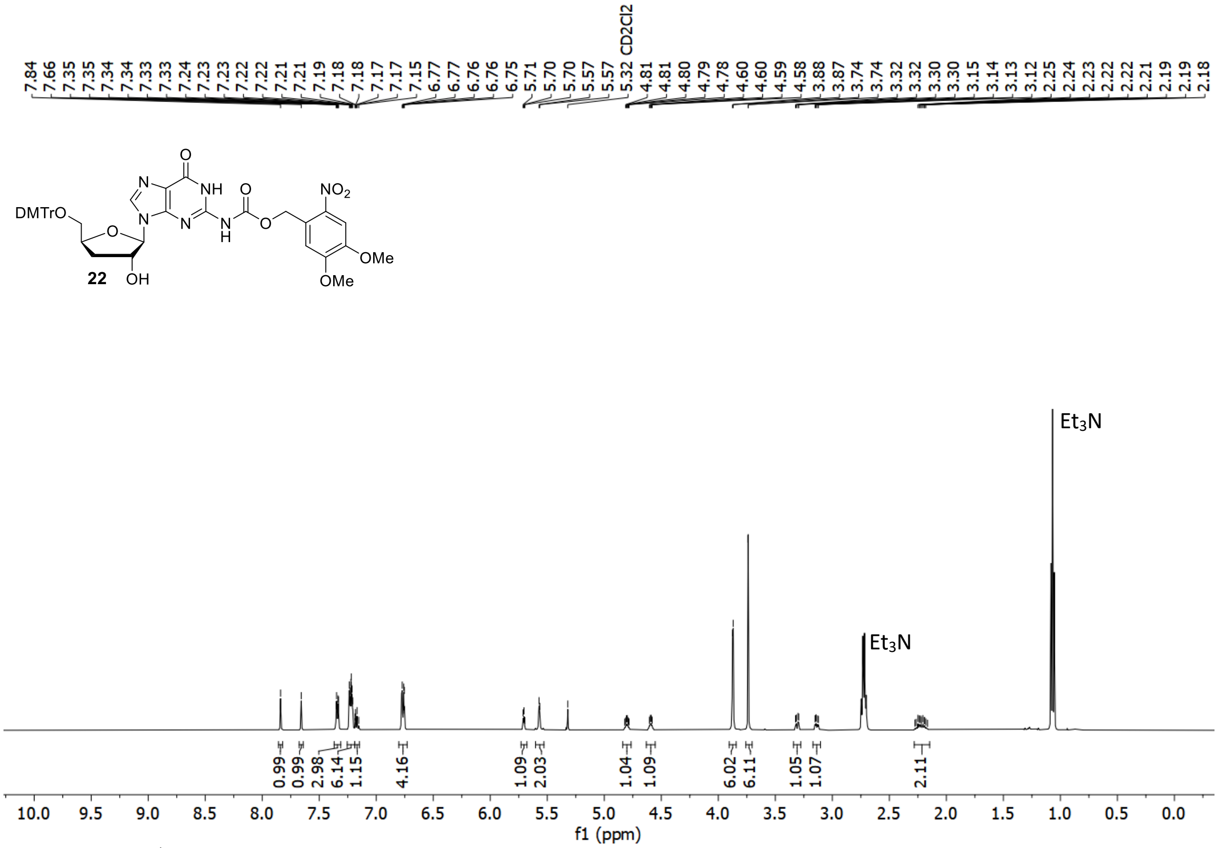

**Figure S14**. $^{1}H$ NMR spectrum of **22**.

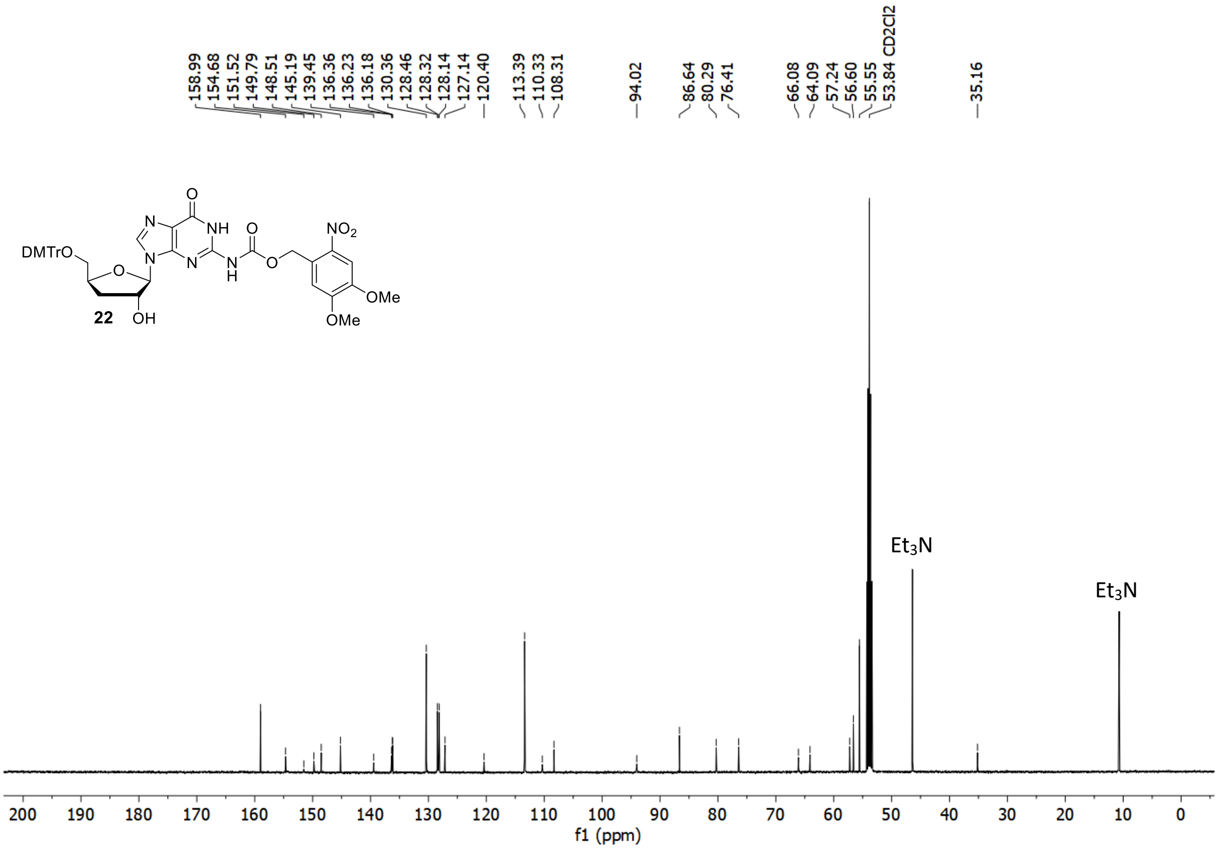

**Figure S15**. $^{13}C\{^{1}H\}$ NMR spectrum of **22**.

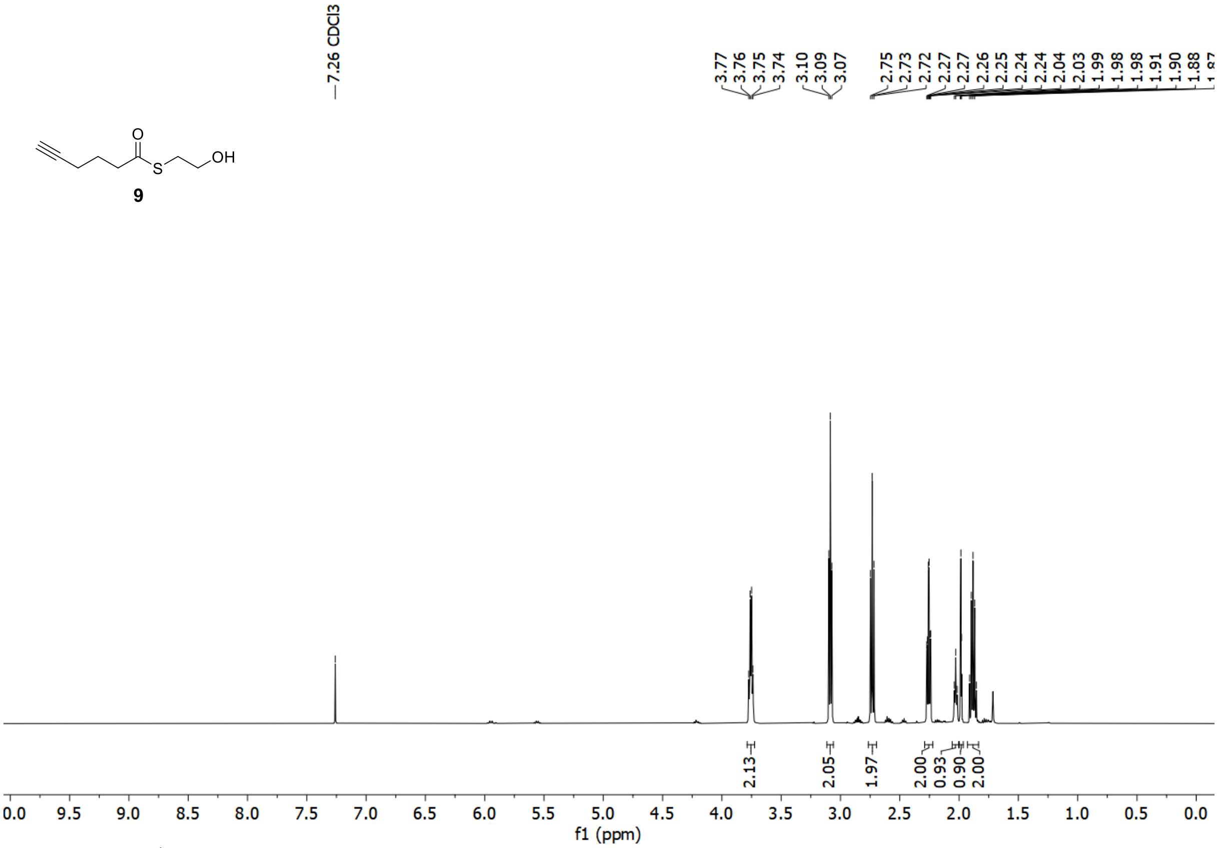


**Figure S16**. $^{1}H$ NMR spectrum of **9**.

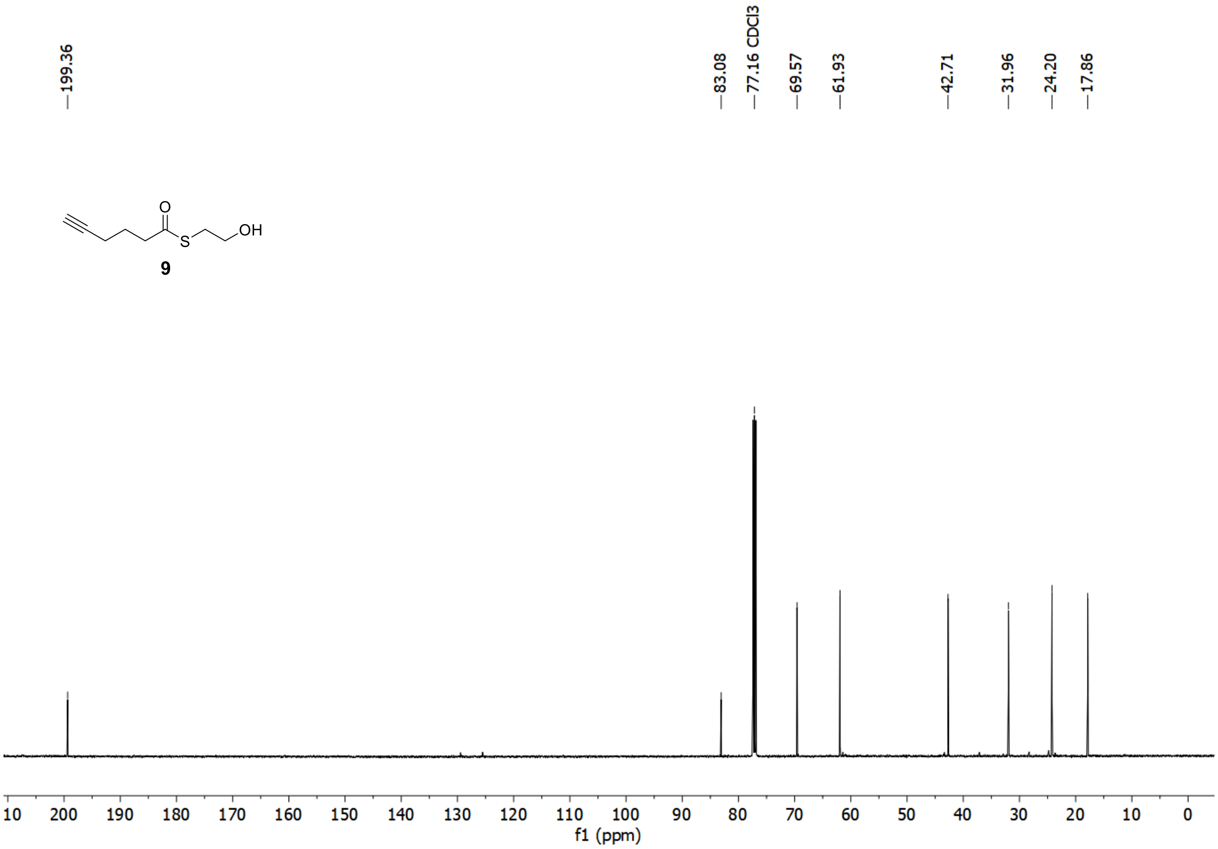


**Figure S17**. $^{13}C\{^{1}H\}$ NMR spectrum of **9**.

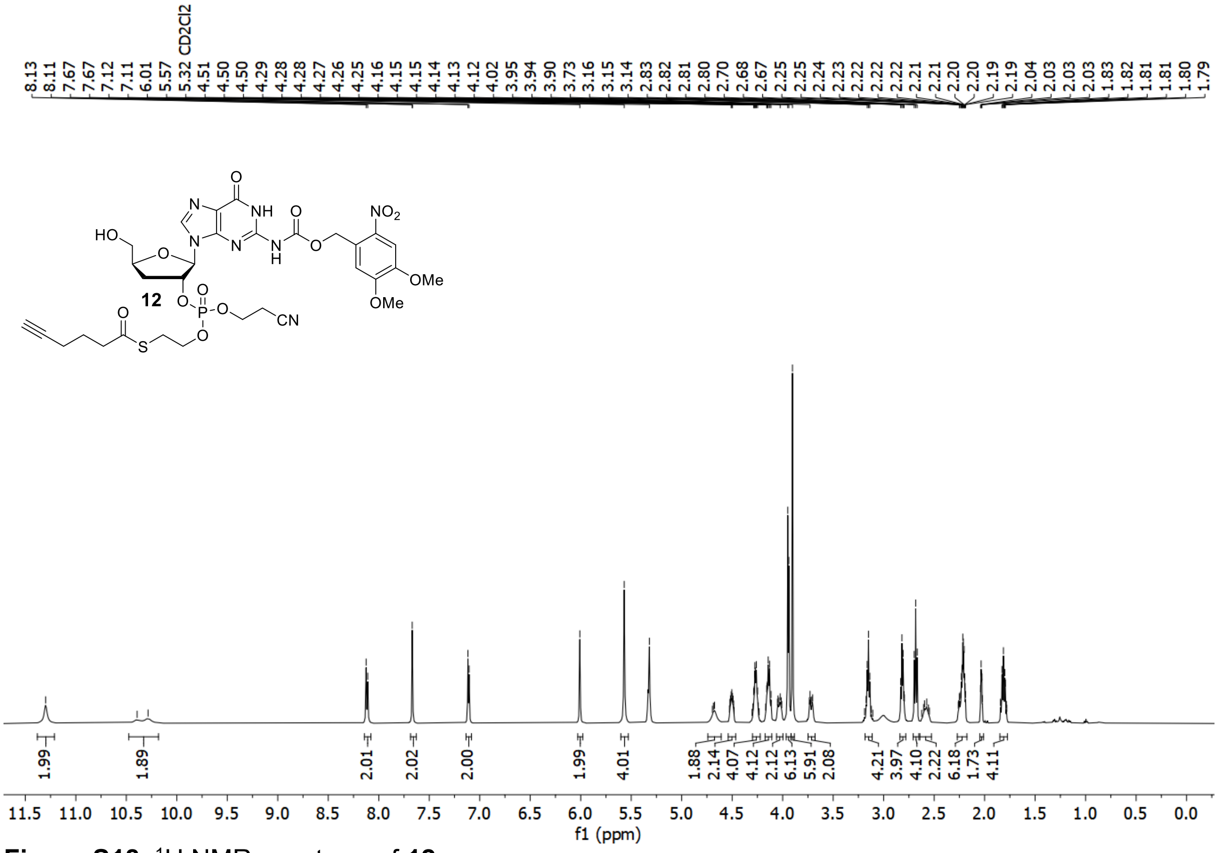


**Figure S18**. $^{1}H$ NMR spectrum of **12**.

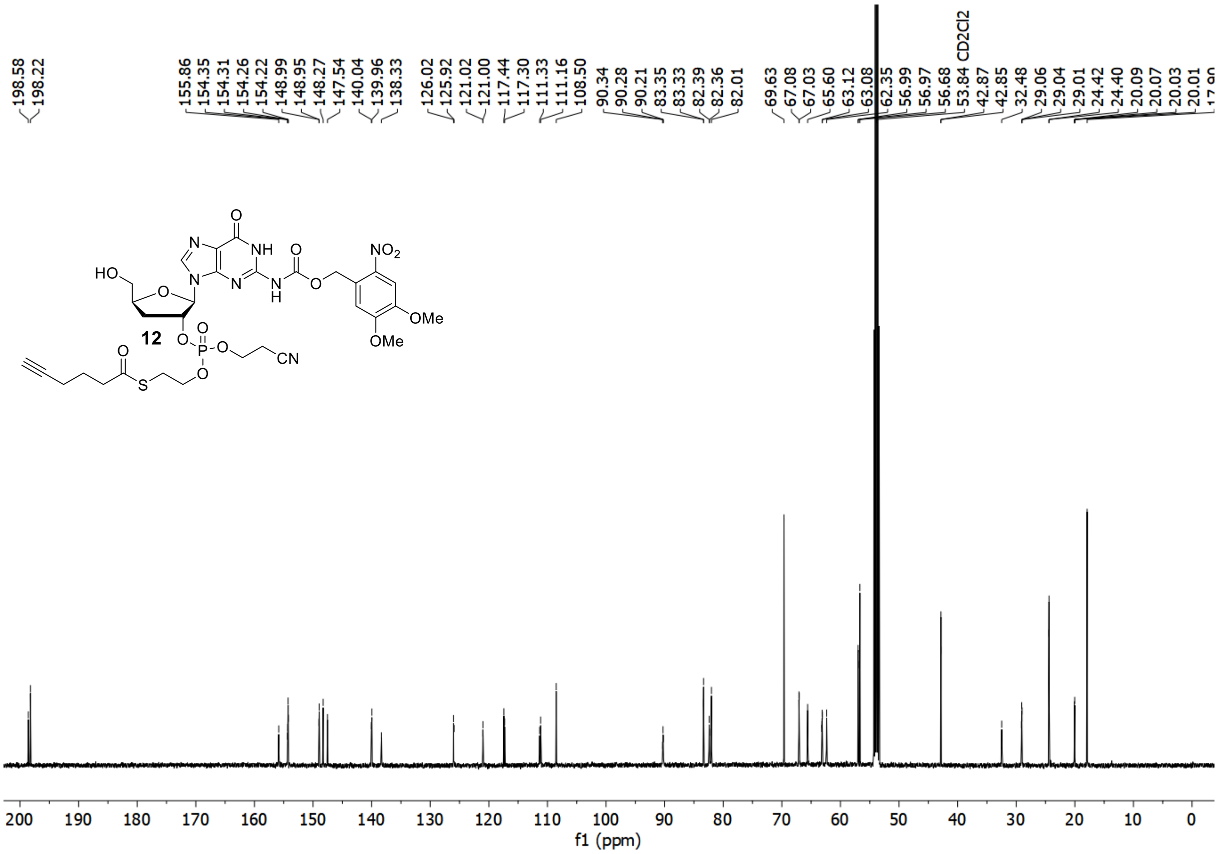


**Figure S19**. $^{13}C\{^{1}H\}$ NMR spectrum of **12**.

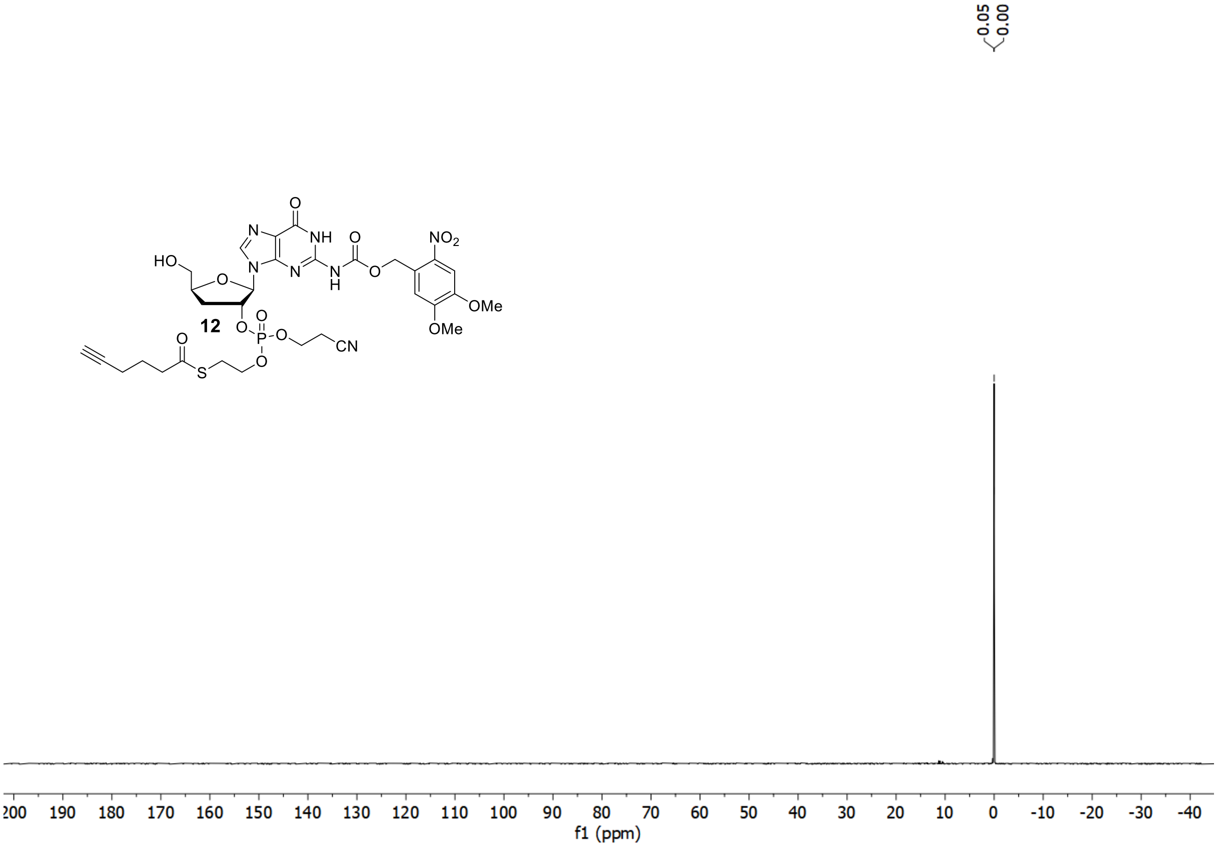


**Figure S20**. $^{31}P$ NMR spectrum of **12**.

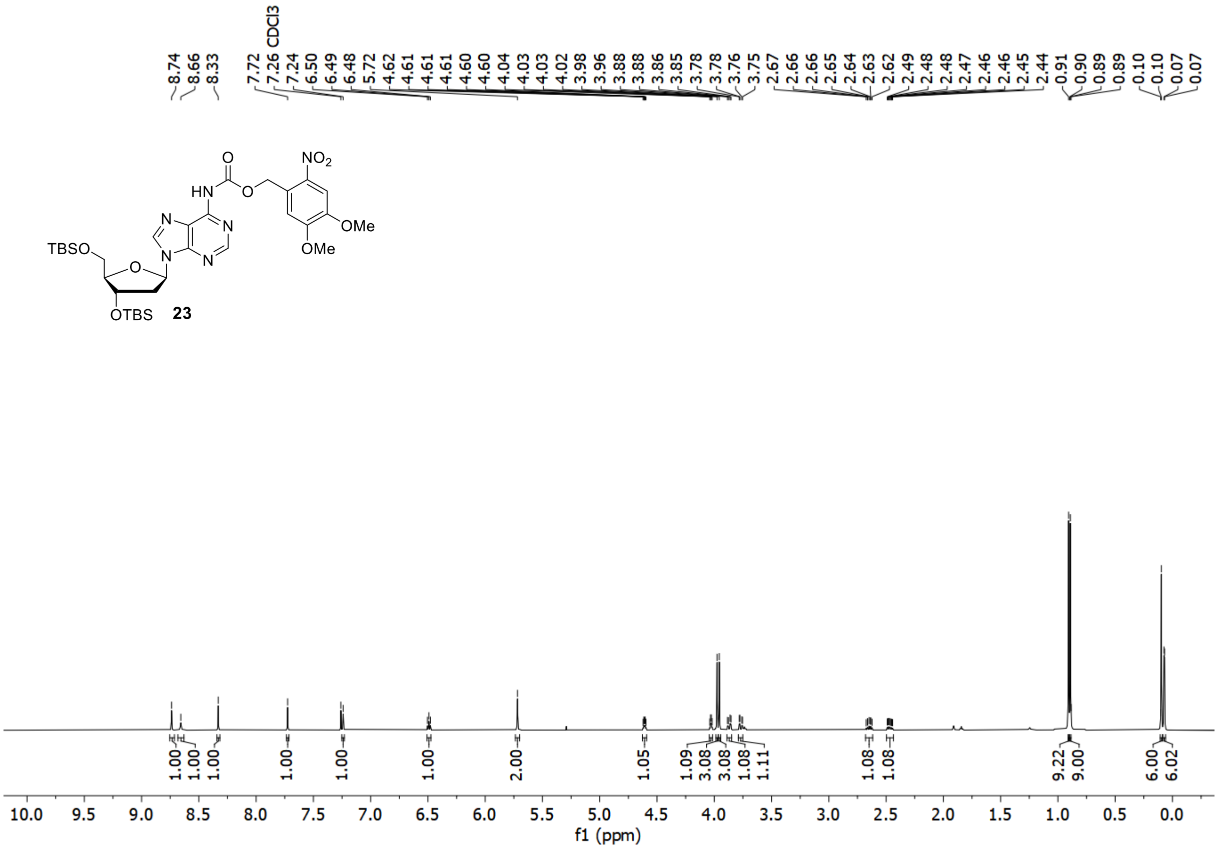


**Figure S21**. $^{1}H$ NMR spectrum of **23**.

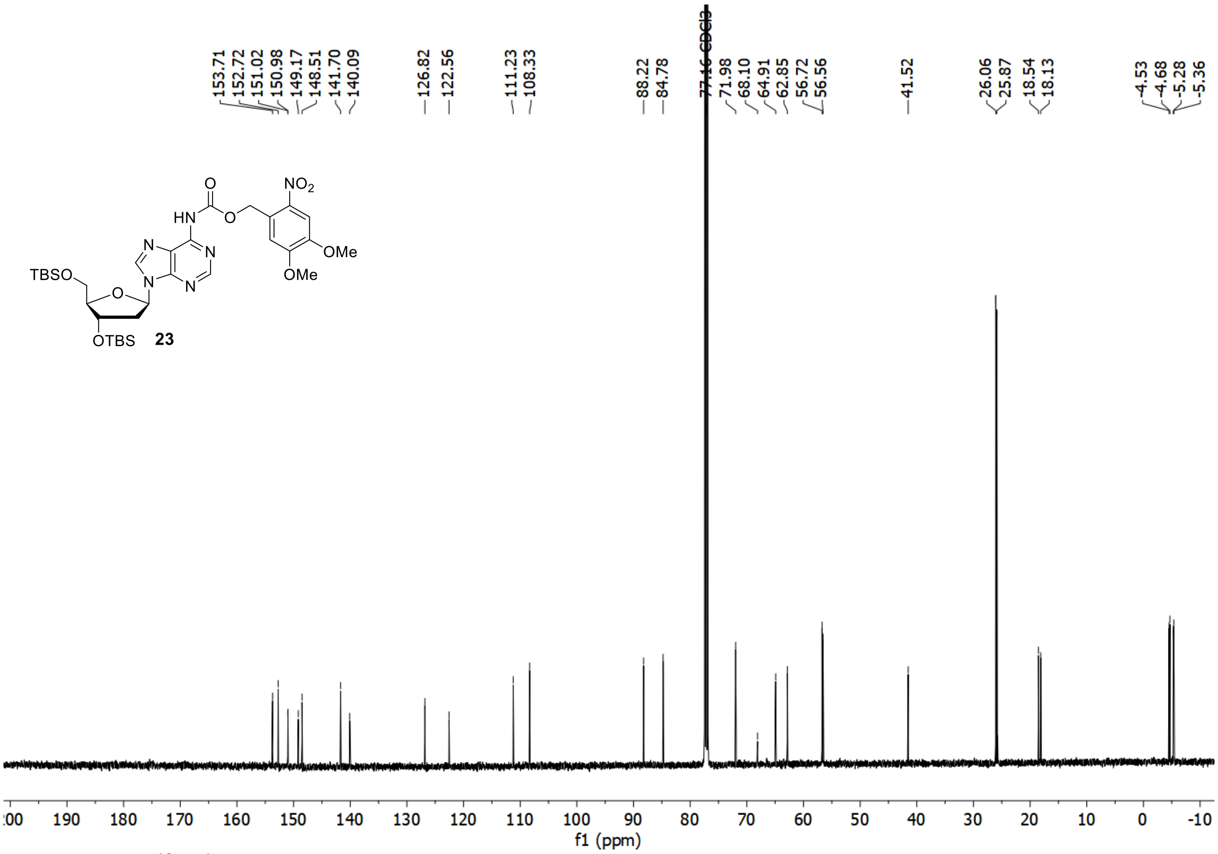


**Figure S22**. $^{13}C\{^1H\}$ NMR spectrum of **23**.

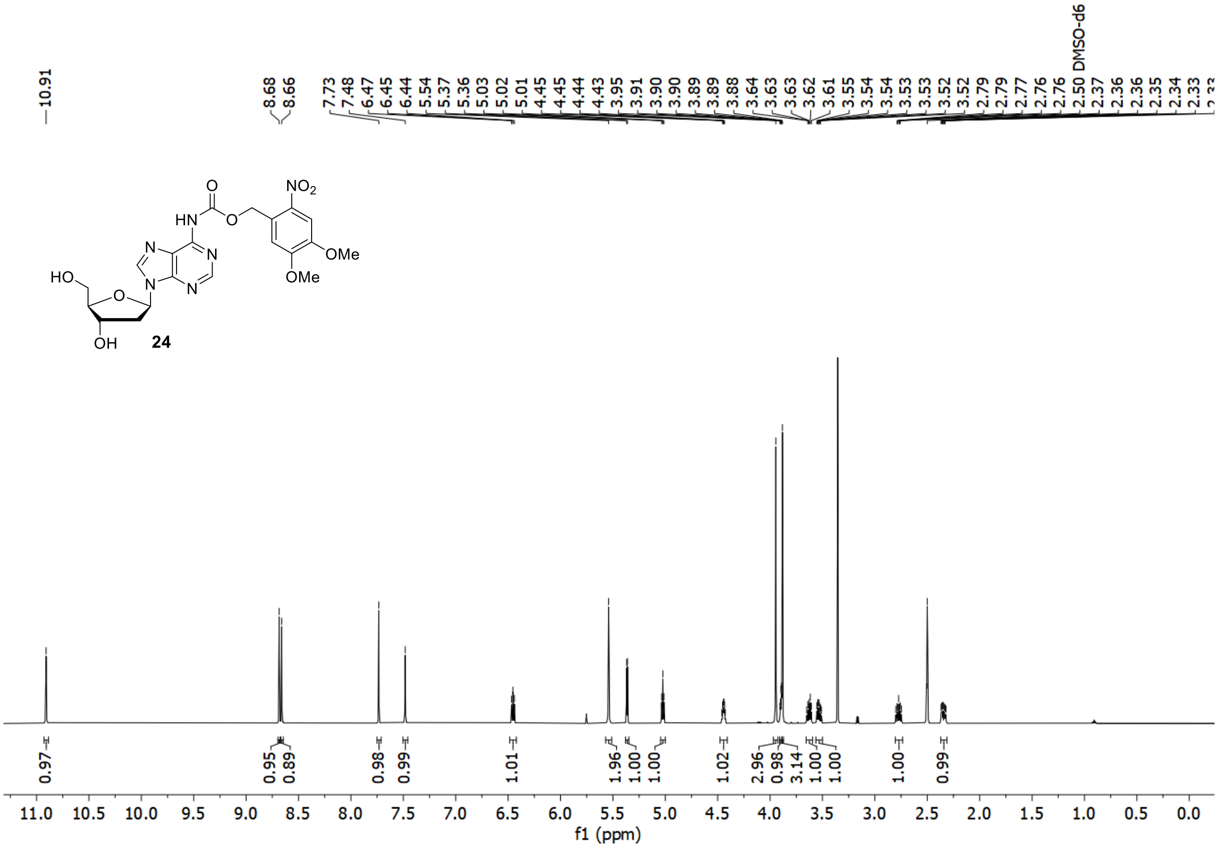


**Figure S23**. $^1H$ NMR spectrum of **24**.

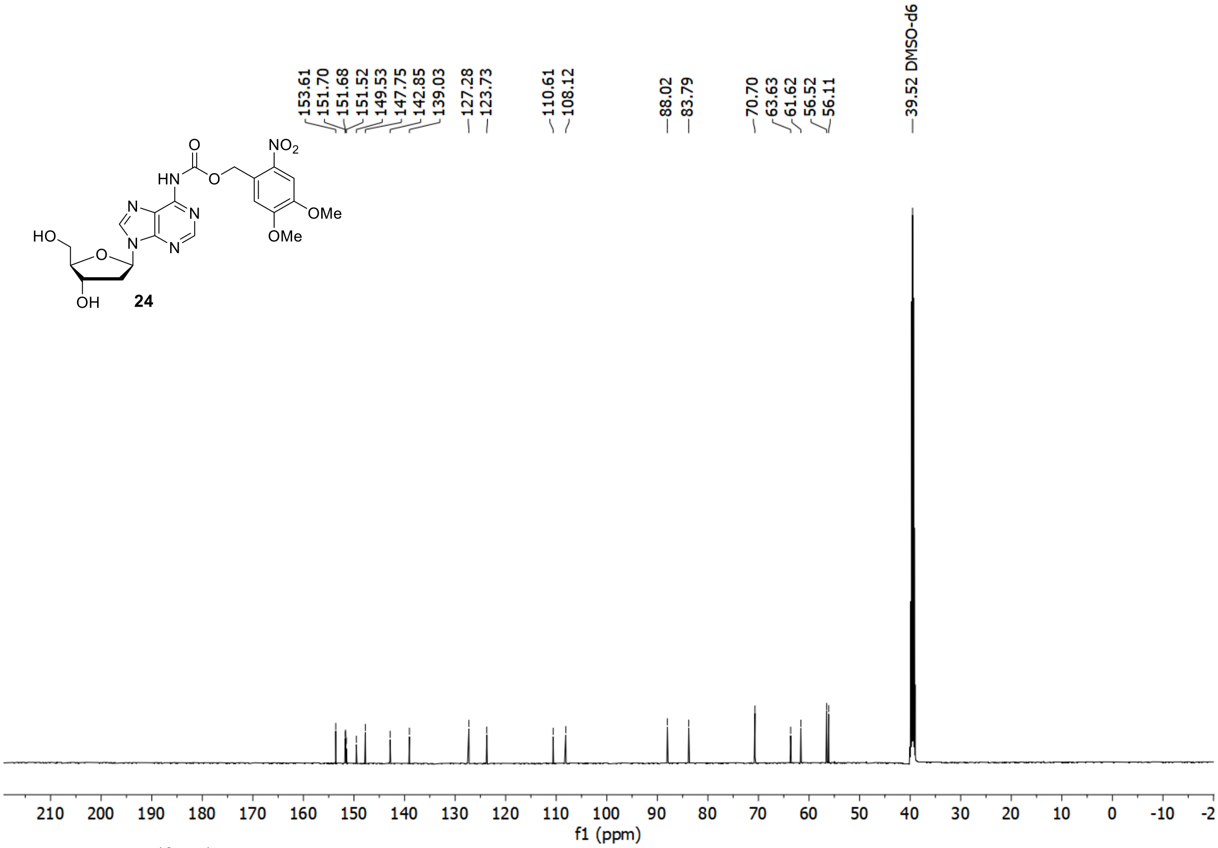


**Figure S24**. $^{13}C\{^{1}H\}$ NMR spectrum of **24**.

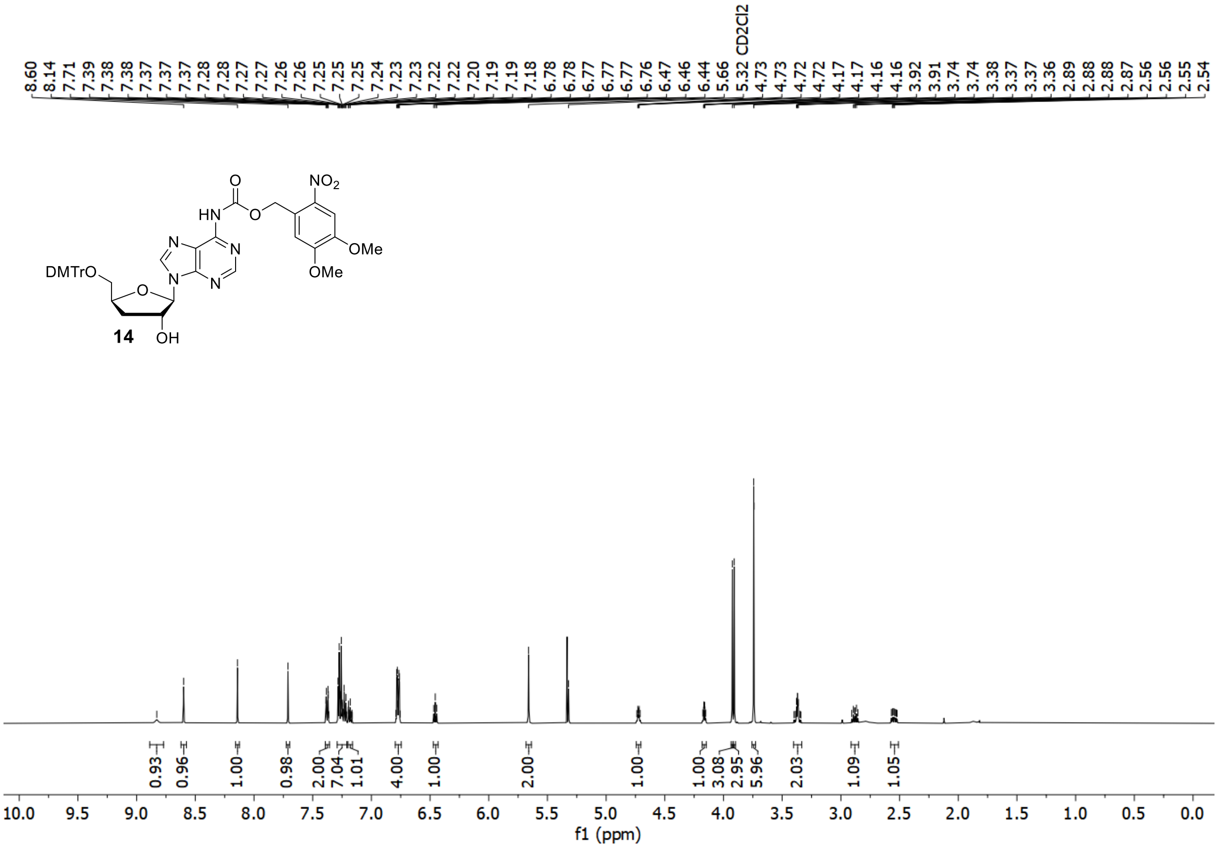


**Figure S25**. $^{1}H$ NMR spectrum of **14**.

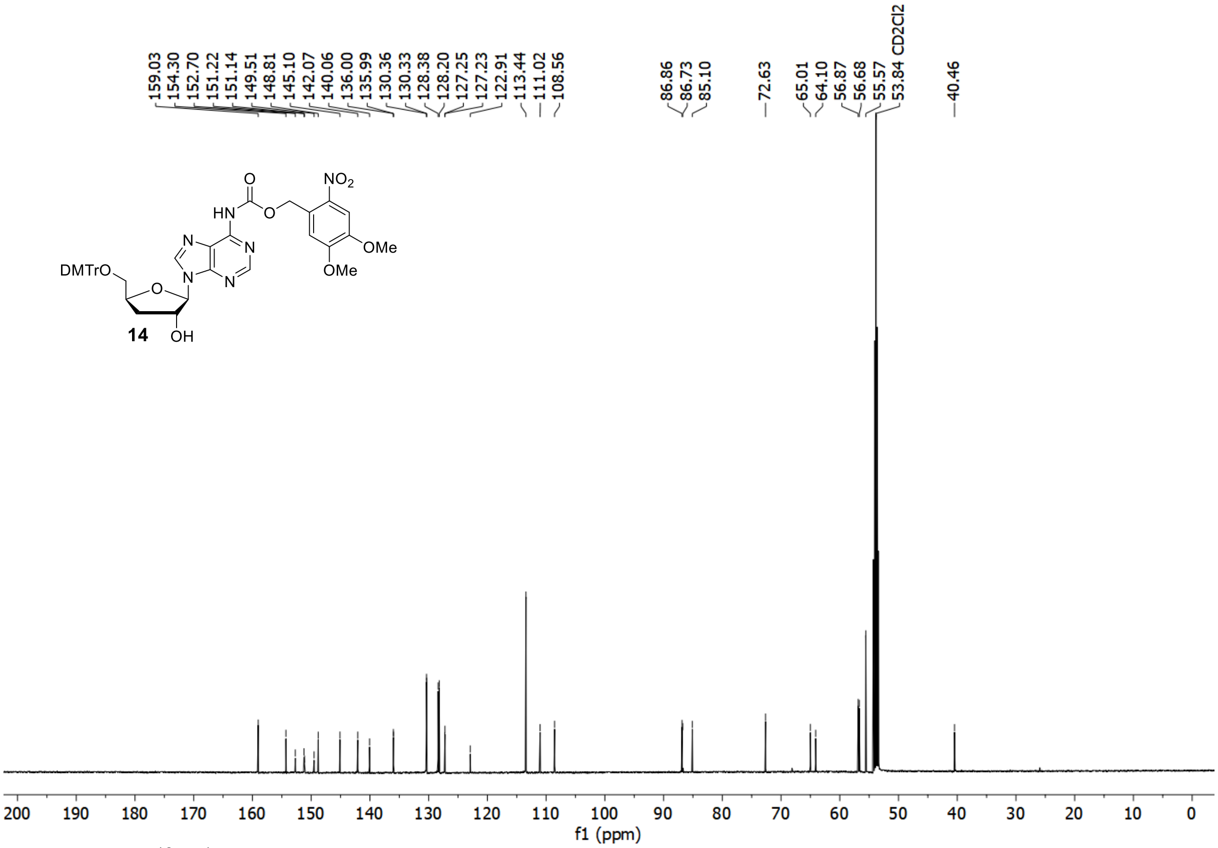


**Figure S26**. $^{13}C\{^{1}H\}$ NMR spectrum of **14**.

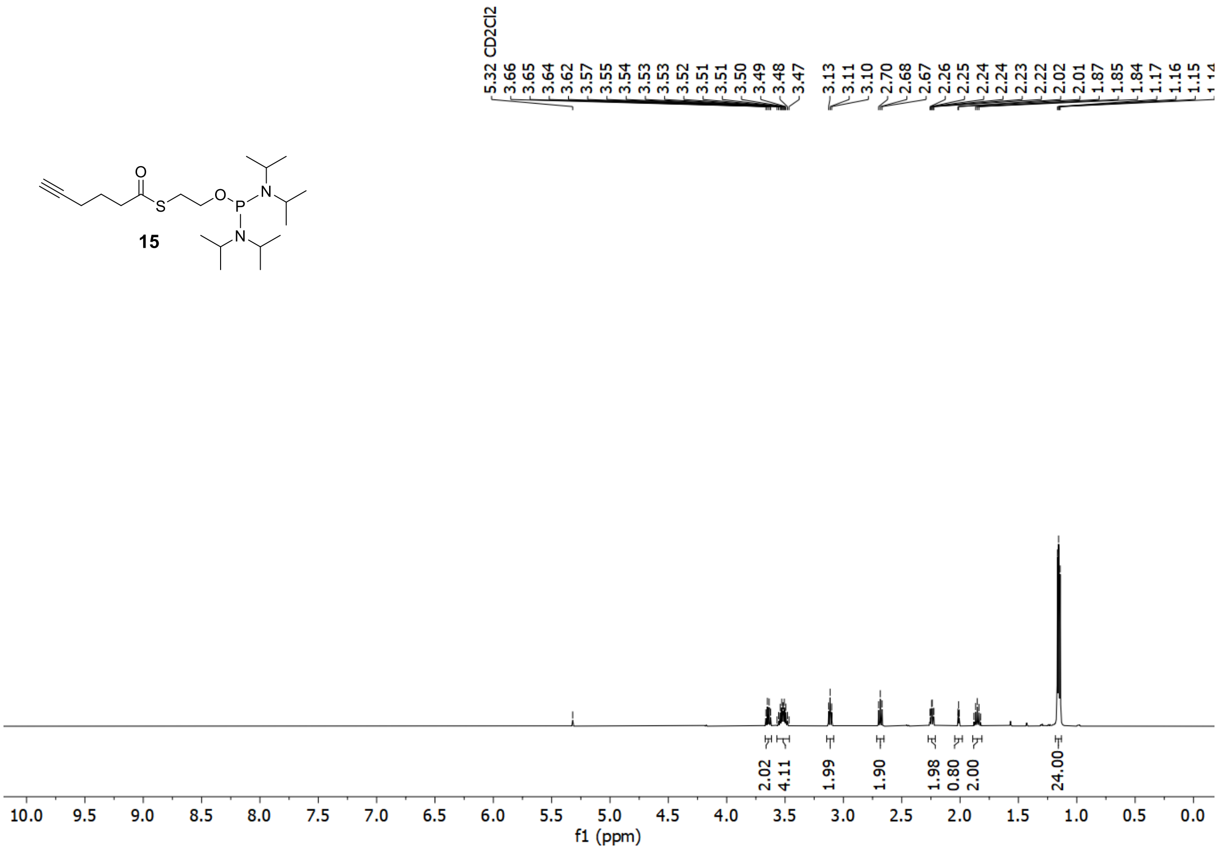


**Figure S27**. $^{1}H$ NMR spectrum of **15**.

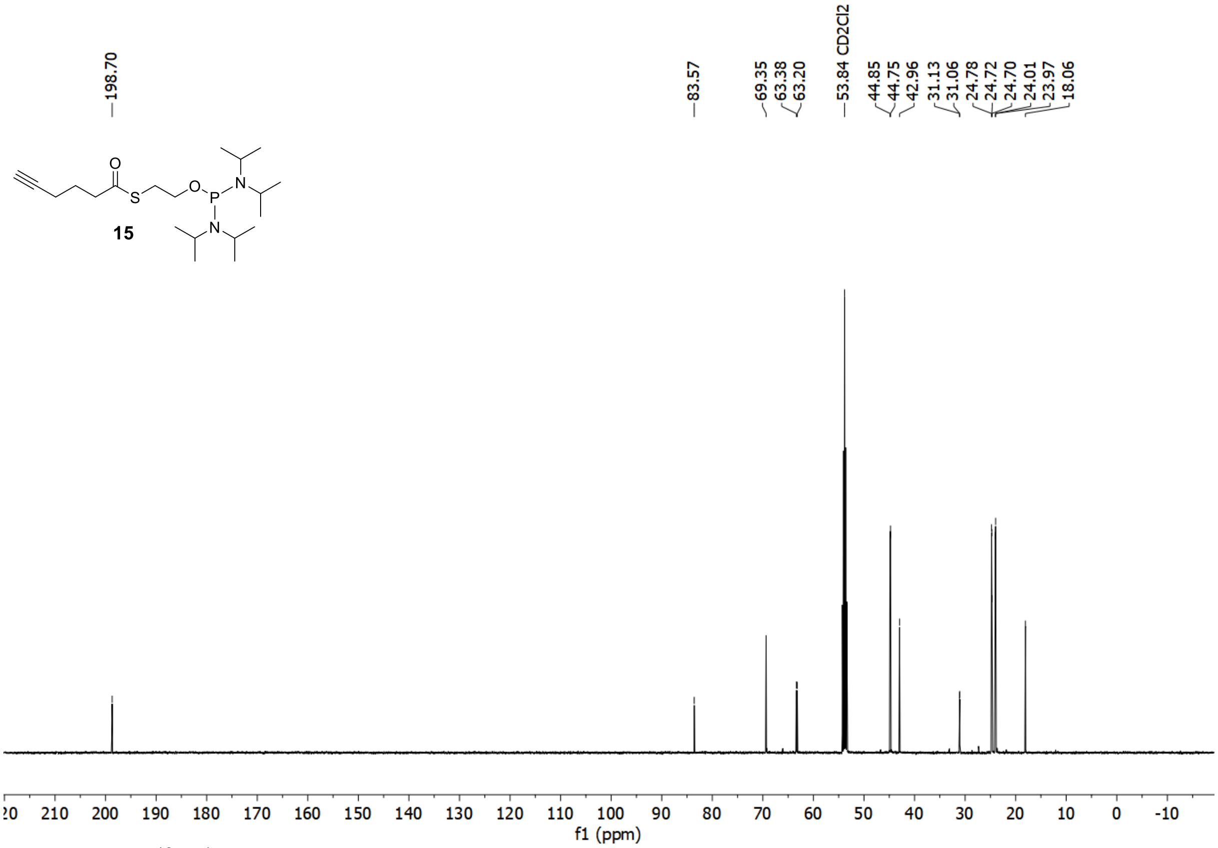


**Figure S28**. $^{13}C\{^{1}H\}$ NMR spectrum of **15**.

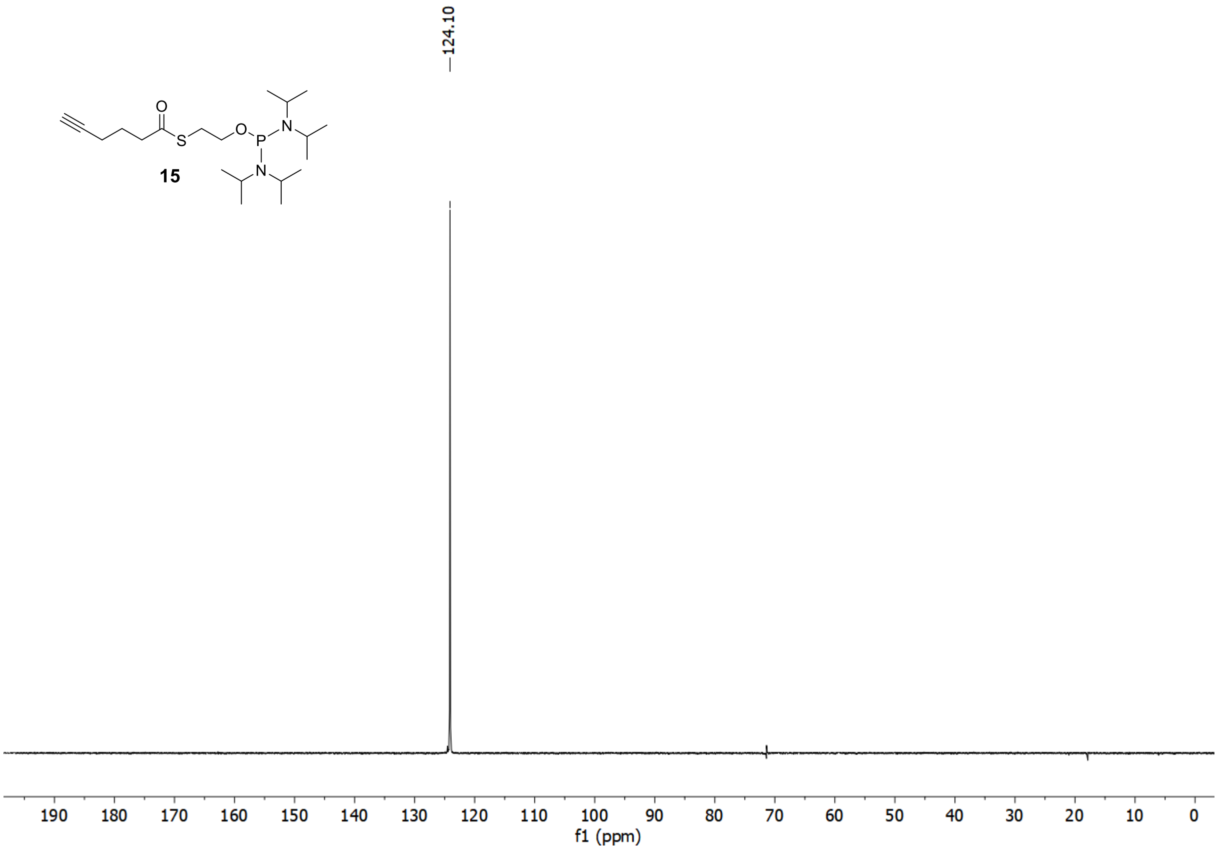


**Figure S29**. $^{31}P$ NMR spectrum of **15**.

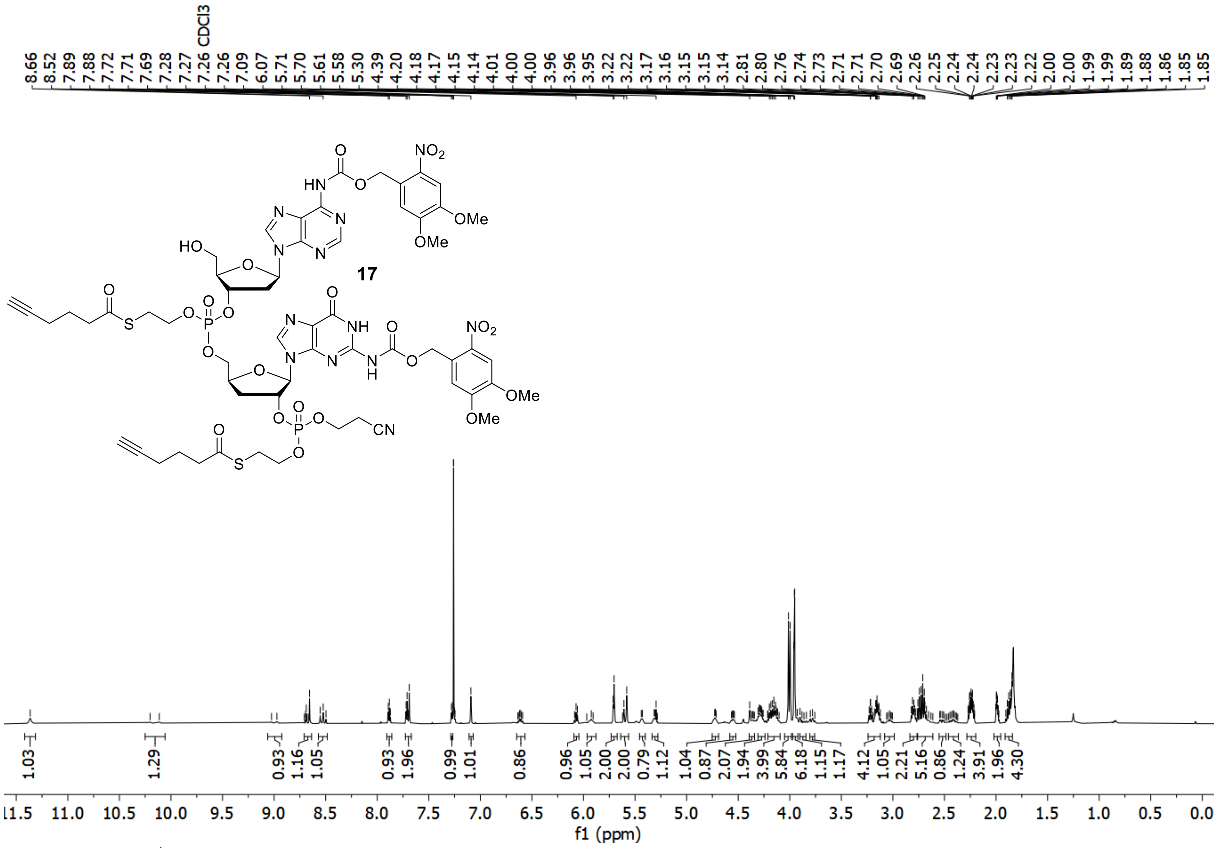

**Figure S30**. $^{1}H$ NMR spectrum of **17**.

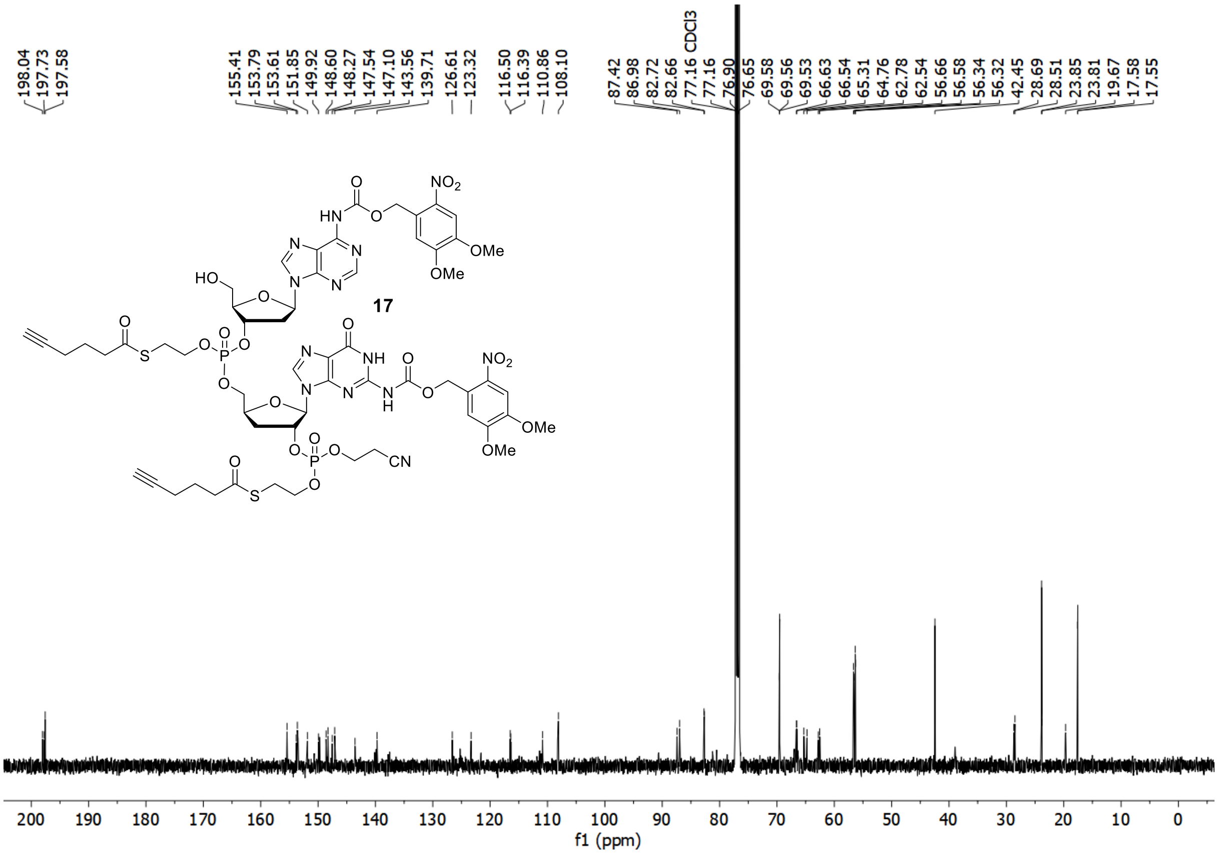

**Figure S31**. $^{13}C\{^{1}H\}$ NMR spectrum of **17**.

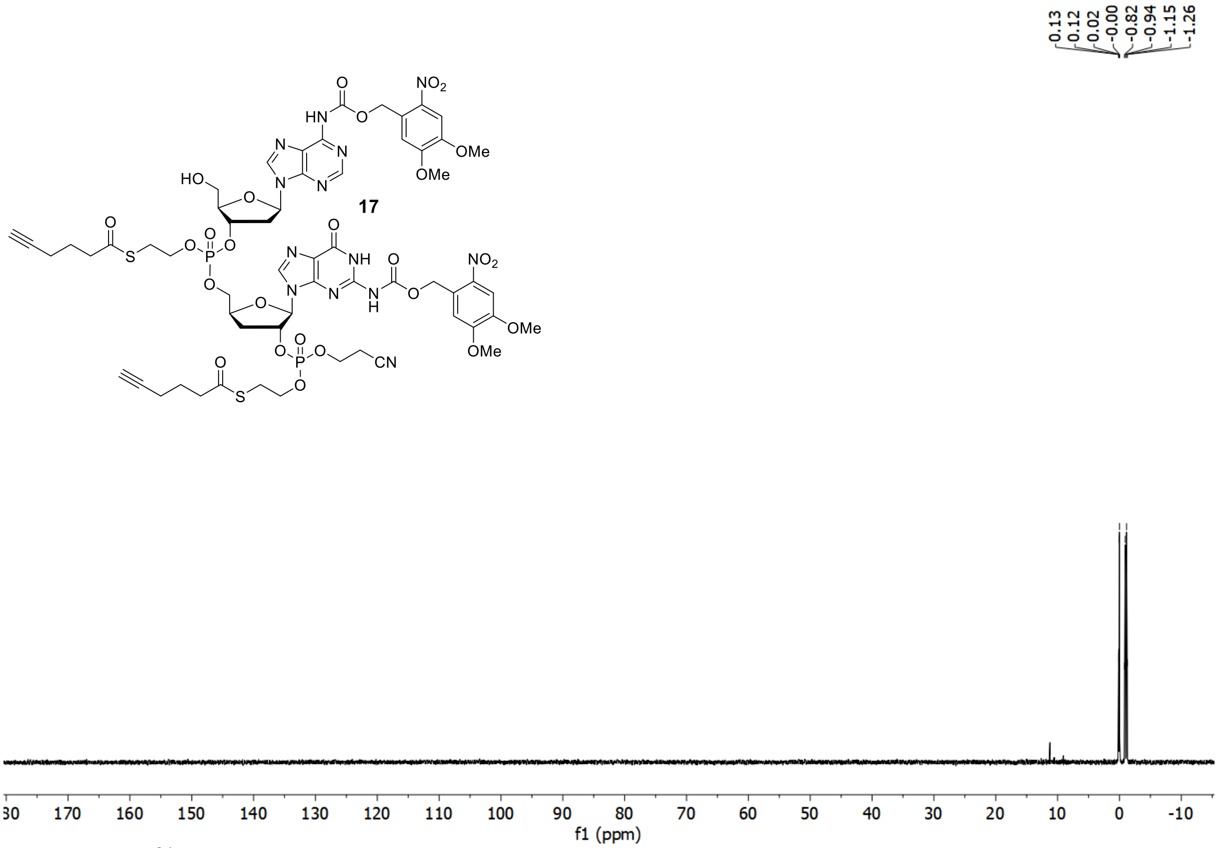


**Figure S32**. $^{31}$P NMR spectrum of **17**.

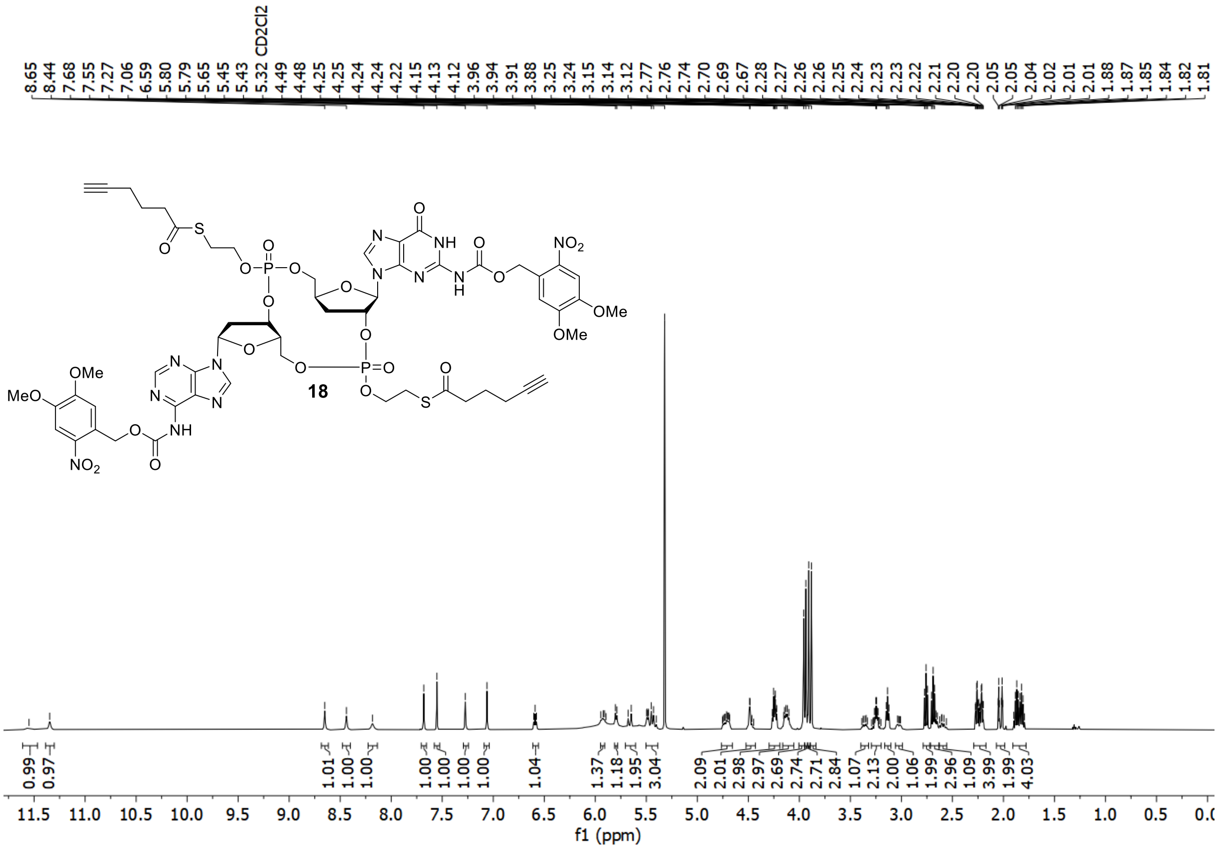


**Figure S33**. $^{1}$H-NMR spectrum of **18**.

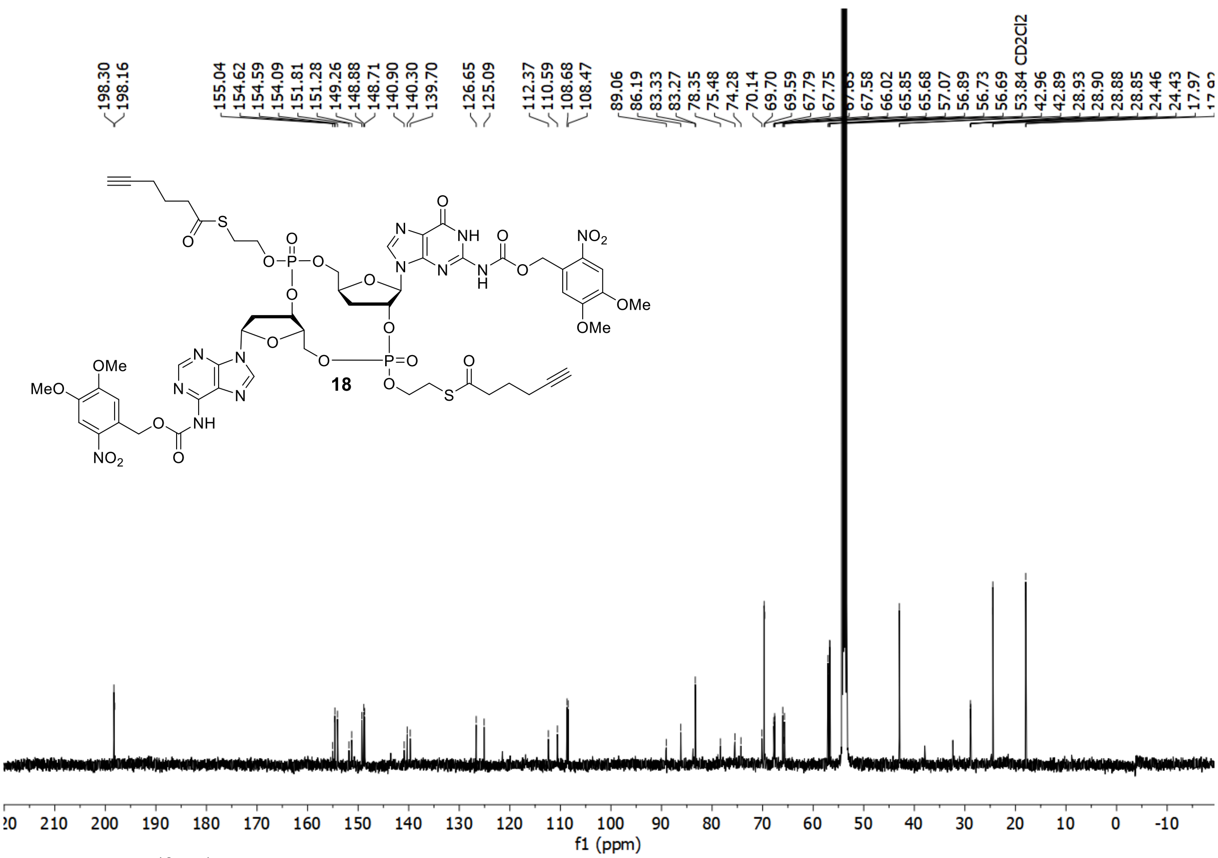


**Figure S34**. $^{13}C\{^1H\}$ NMR spectrum of **18**.

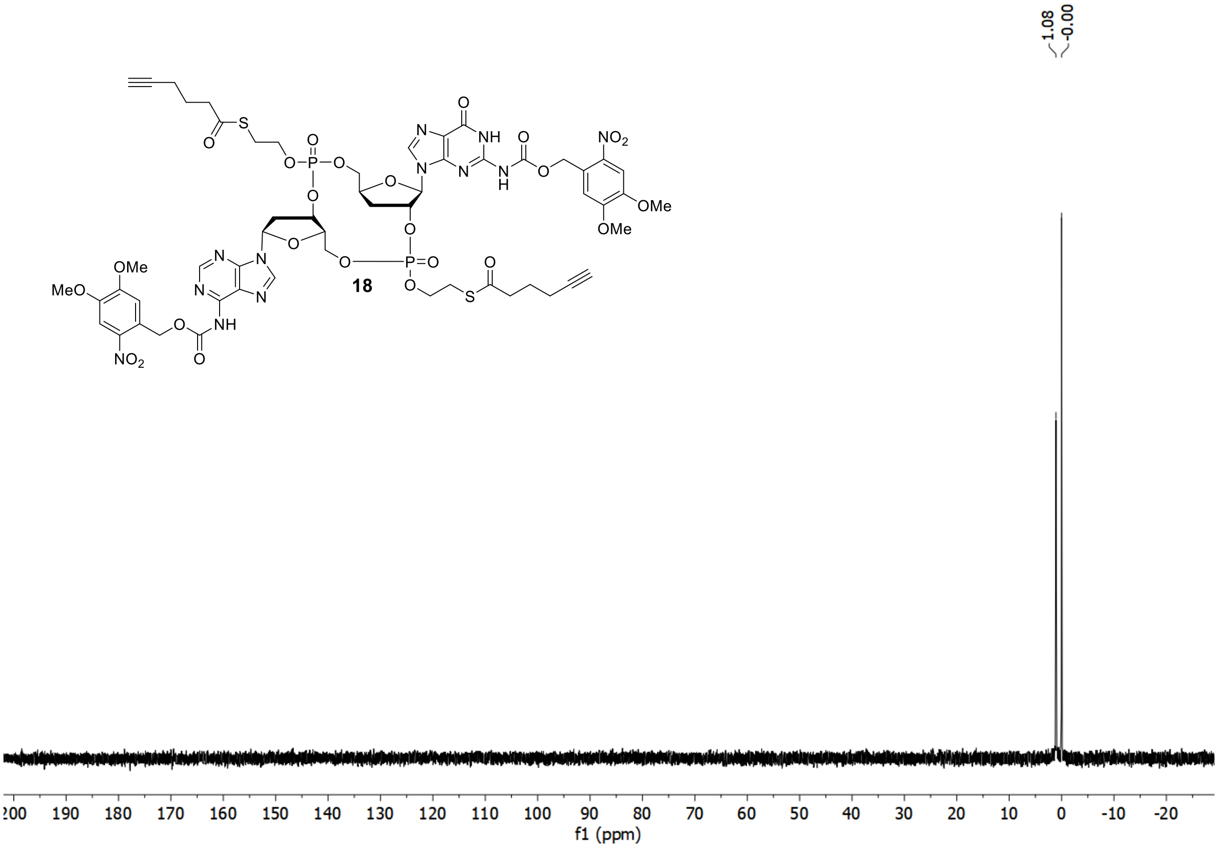


**Figure S35**. $^{31}P$ NMR spectrum of **18**.

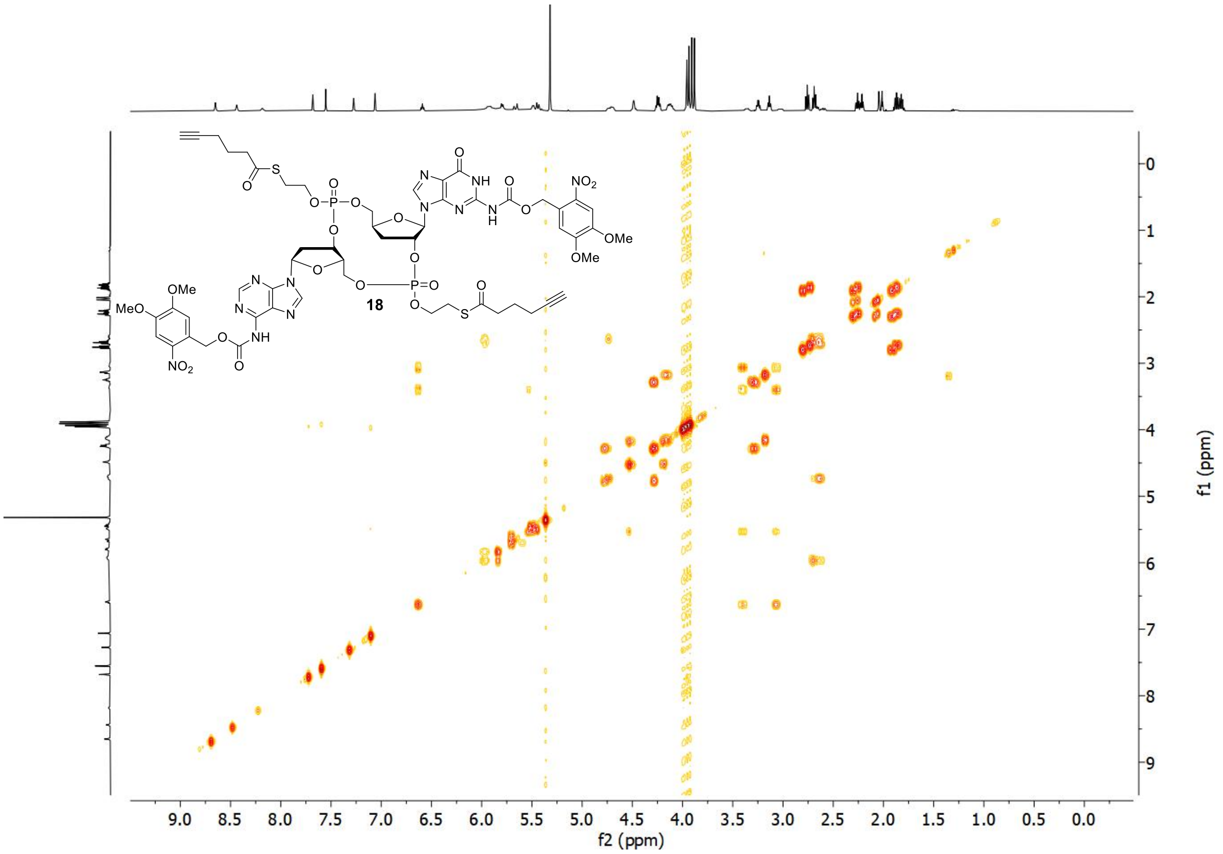


**Figure S36**. COSY-NMR spectrum of **18**.

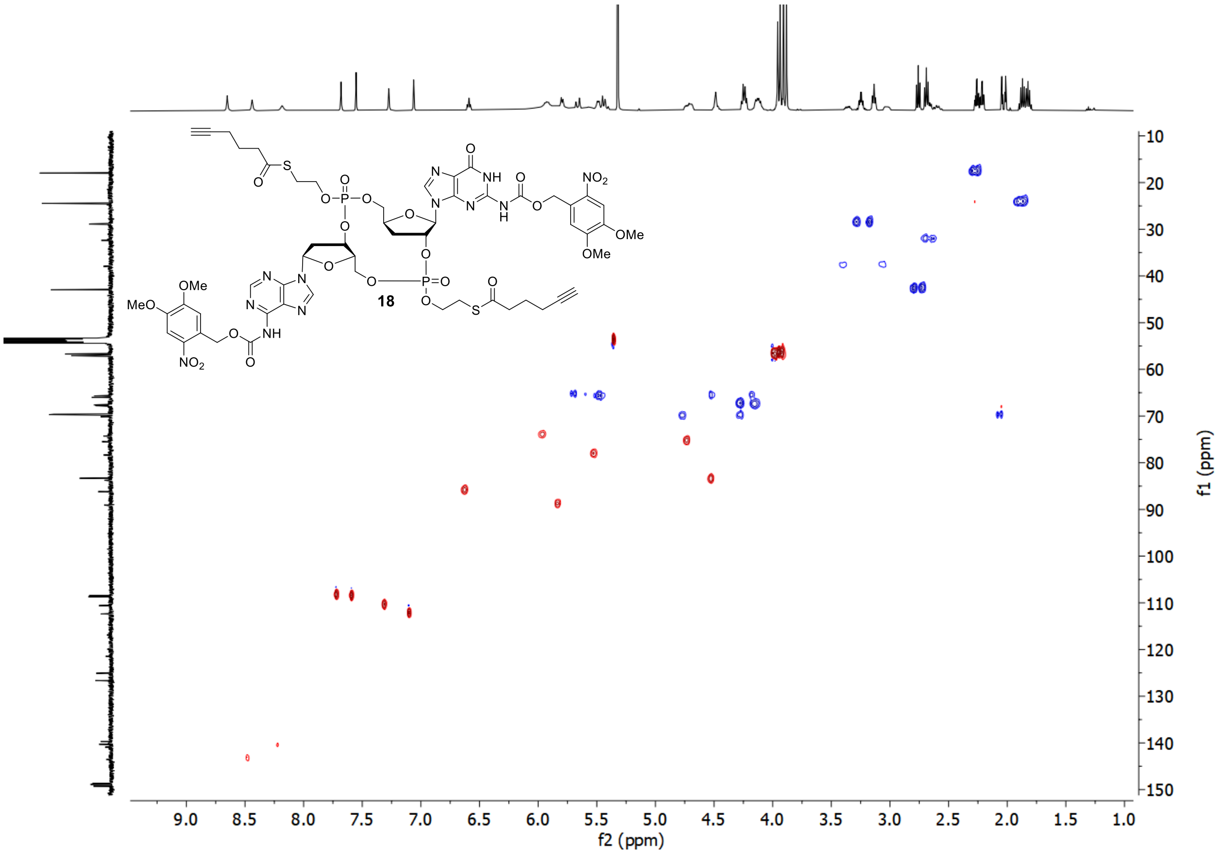


**Figure S37**. HSQC NMR spectrum of **18**.

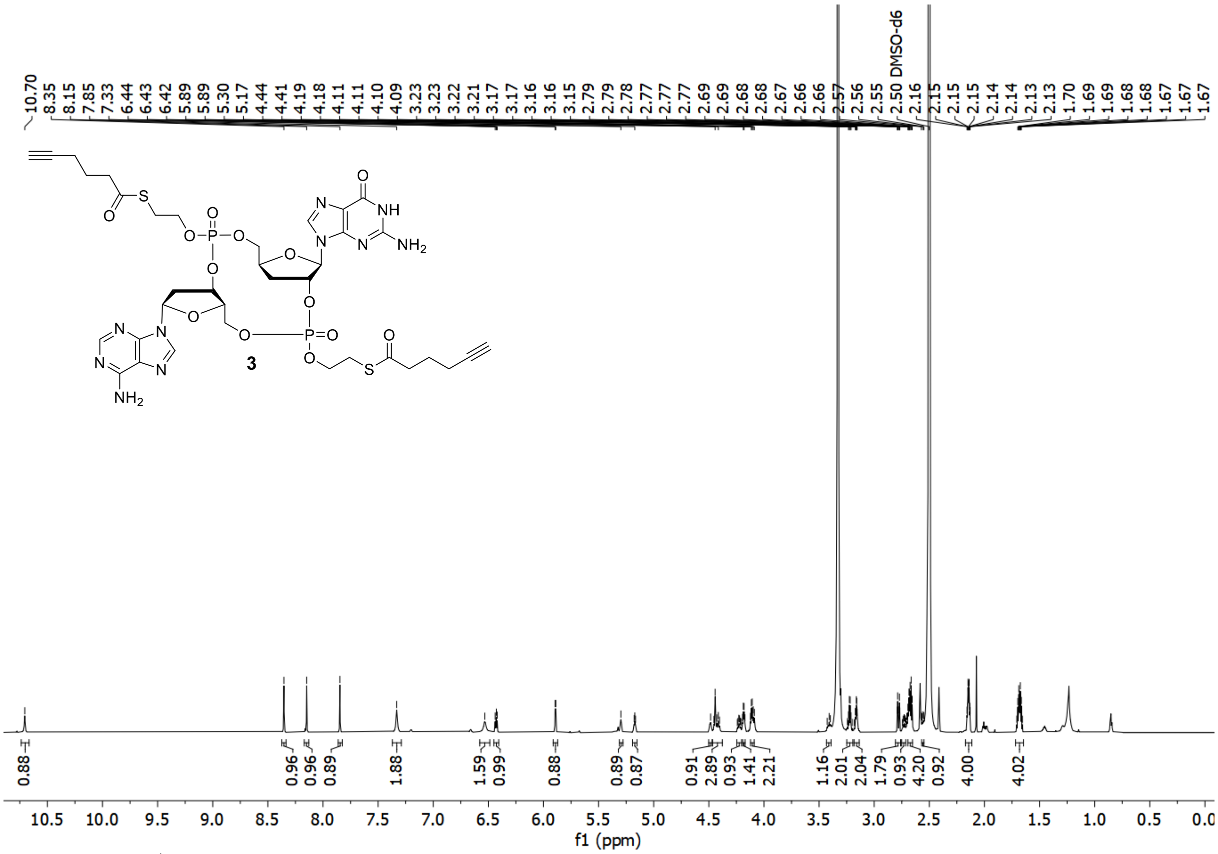


**Figure S38**. $^{1}$H NMR spectrum of **3**.

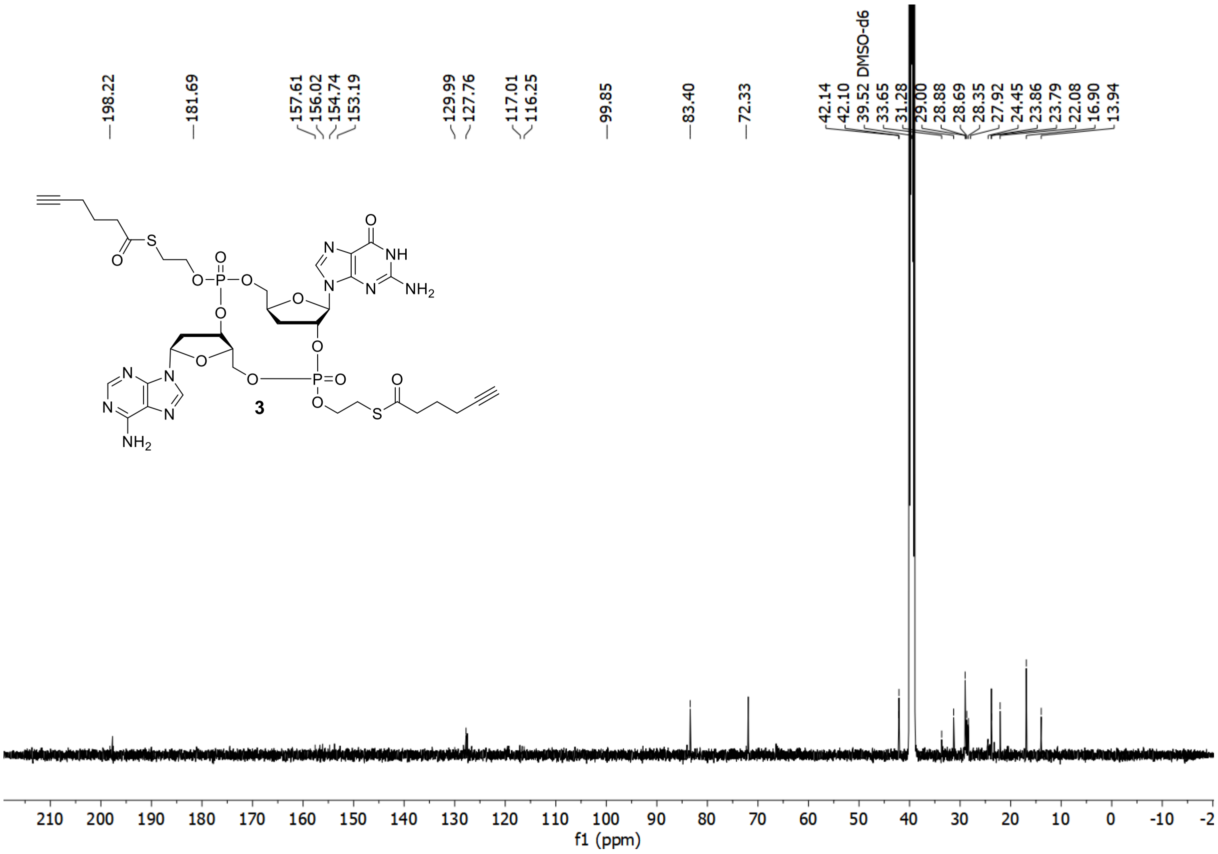


**Figure S39**. $^{13}$C{$^{1}$H} NMR spectrum of **3**.

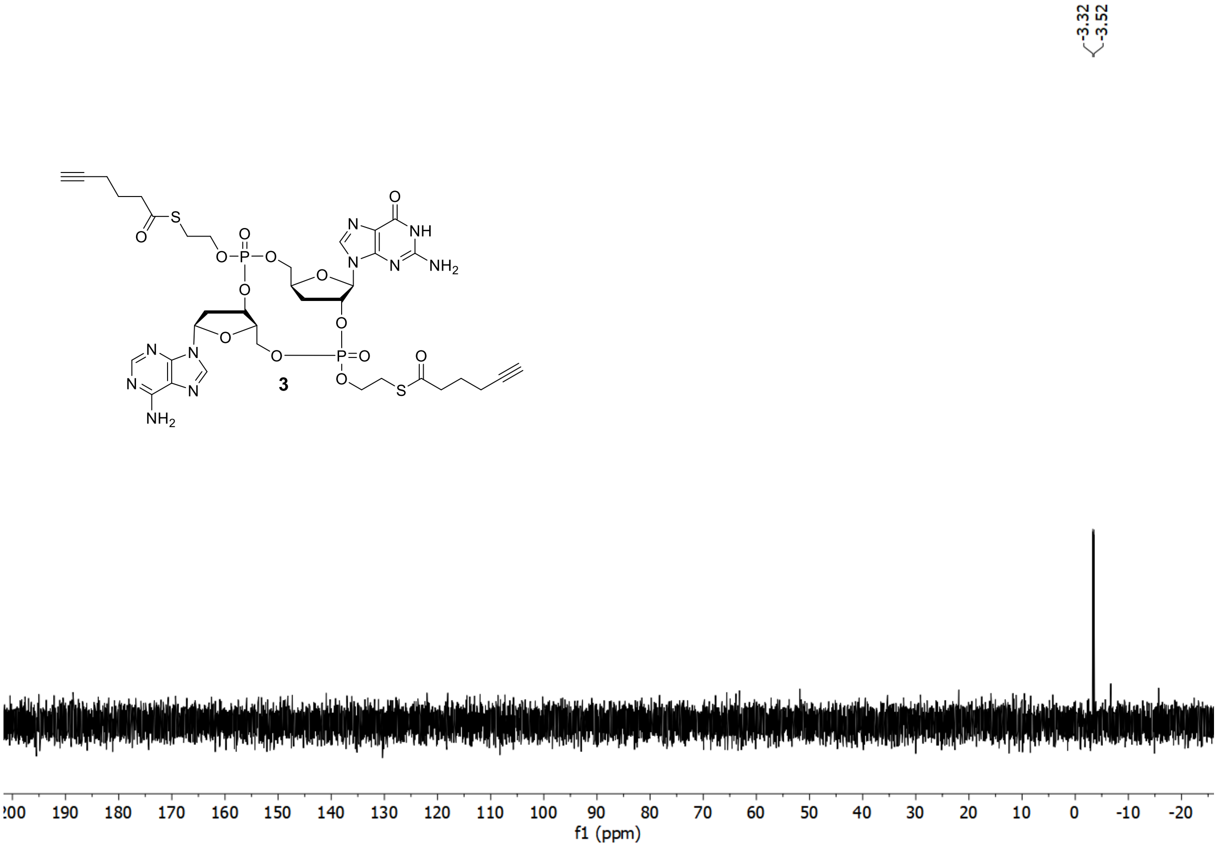


**Figure S40**. $^{31}P$ NMR spectrum of **3**.

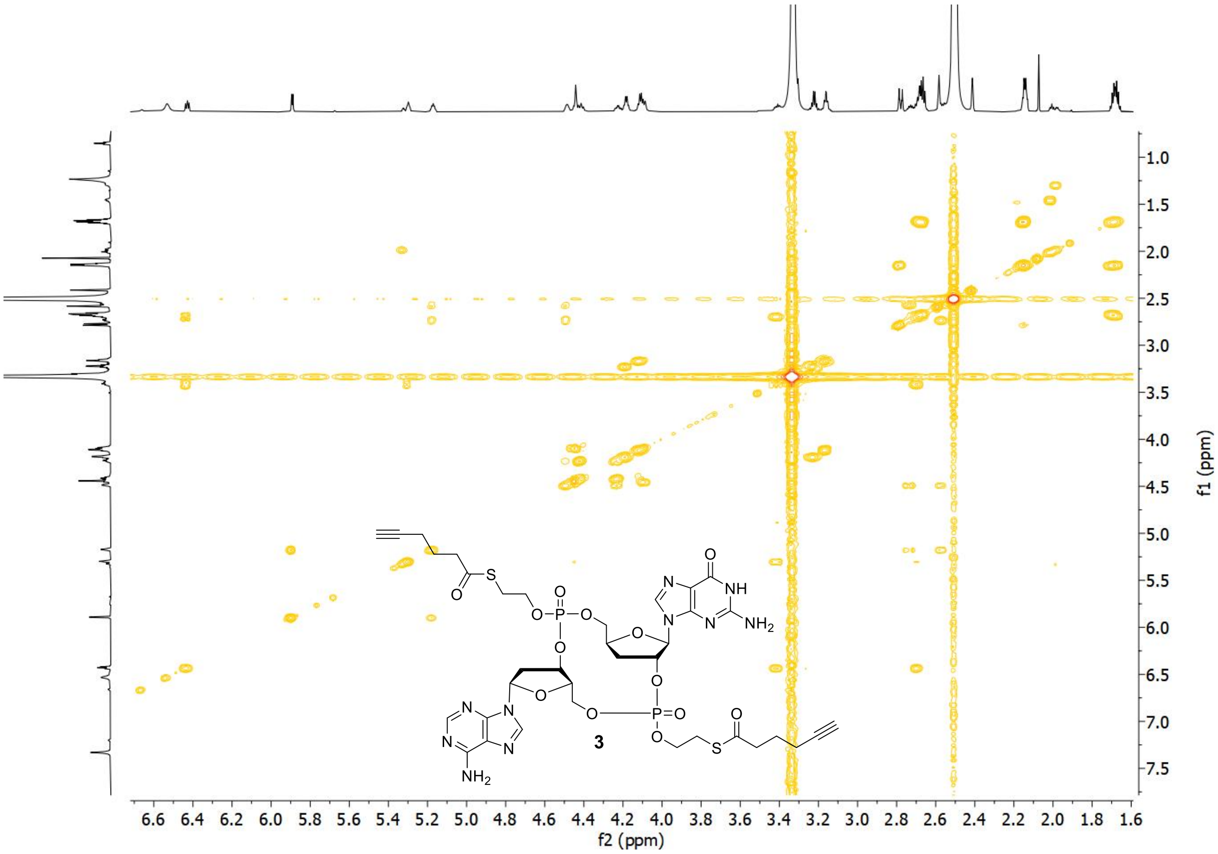


**Figure S41**. COSY NMR spectrum of **3**.

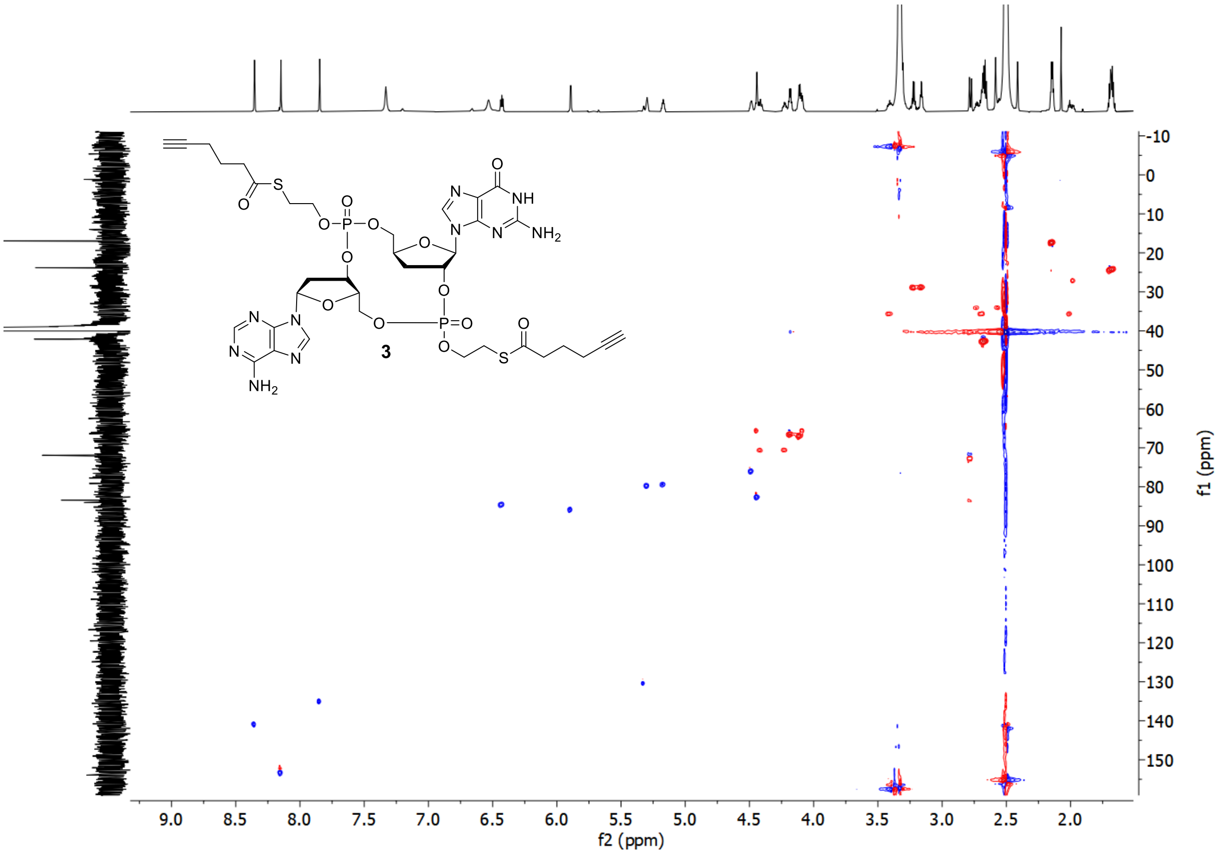

**Figure S42**. HSQC NMR spectrum of **3**.

## 6 Reference


[1] S. Stazzoni, D. F. R. Böhmer, F. Hernichel, D. Özdemir, A. Pappa, D. Drexler, S. Bauernfried, G. Witte, M. Wagner, S. Veth, K. P. Hopfner, V. Hornung, L. M. König, T. Carell, *Angew. Chem. Int. Ed.* **2022**, *61*, e202207175.

[2] F. Hulpia, S. V. Calenbergh, G. Caljon, L. Maes, WO2019076633 (Ed.: U. A. Universiteit Gent), **2019**.

[3] H. An, E. Gunic, Y.-H. Koh, H. Chen, D. Barawkar, W. Zhang, J.-L. Girardet, F. Rong, Z. Hong, *WO2003051881A1* (Ed.: R. Inc.), **2003**.

[4] M. J. Robins, R. Zou, Z. Guo, S. F. Wnuk, *J. Org. Chem.* **1996**, *61*, 9207-9212.

[5] A. Wiest, P. Kielkowski, *J. Am. Chem. Soc.* **2024**, *146,* 2151-2159.

[6] M. C. Chambers, B. Maclean, R. Burke, D. Amodei, D. L. Ruderman, S. Neumann, L. Gatto, B. Fischer, B. Pratt, J. Egertson, K. Hoff, D. Kessner, N. Tasman, N. Shulman, B. Frewen, T. A. Baker, M. Y. Brusniak, C. Paulse, D. Creasy, L. Flashner, K. Kani, C. Moulding, S. L. Seymour, L. M. Nuwaysir, B. Lefebvre, F. Kuhlmann, J. Roark, P. Rainer, S. Detlev, T. Hemenway, A. Huhmer, J. Langridge, B. Connolly, T. Chadick, K.

Holly, J. Eckels, E. W. Deutsch, R. L. Moritz, J. E. Katz, D. B. Agus, M. MacCoss, D. L. Tabb, P. Mallick, *Nat. Biotechnol.* **2012**, *30*, 918-920.

[7] V. Demichev, C. B. Messner, S. I. Vernardis, K. S. Lilley, M. Ralser, *Nat. Methods* **2020**, *17*, 41-44.

[8] S. Tyanova, T. Temu, P. Sinitcyn, A. Carlson, M. Y. Hein, T. Geiger, M. Mann, J. Cox, *Nat. Methods* **2016**, *13*, 731-740.

[9] B. T. Sherman, M. Hao, J. Qiu, X. Jiao, M. W. Baseler, H. C. Lane, T. Imamichi, W. Chang, *Nucleic Acids Res.* **2022**, *50*, W216-w221.
